# Purification of cone outer segment for proteomic analysis on its membrane proteins in carp retina

**DOI:** 10.1371/journal.pone.0173908

**Published:** 2017-03-14

**Authors:** Takashi Fukagawa, Kazuaki Takafuji, Shuji Tachibanaki, Satoru Kawamura

**Affiliations:** 1 Graduate School of Frontier Biosciences, Osaka University, Suita, Osaka, Japan; 2 Center of Medical Innovation and Translational Research, Graduate School of Medicine, Osaka University, Suita, Osaka, Japan; 3 Department of Biological Sciences, Graduate School of Science, Osaka University, Suita, Osaka, Japan; Carl von Ossietzky Universitat Oldenburg, GERMANY

## Abstract

Rods and cones are both photoreceptors in the retina, but they are different in many aspects including the light response characteristics and, for example, cell morphology and metabolism. These differences would be caused by differences in proteins expressed in rods and cones. To understand the molecular bases of these differences between rods and cones, one of the ways is to compare proteins expressed in rods and cones, and to find those expressed specifically or dominantly. In the present study, we are interested in proteins in the outer segment (OS), the site responsible for generation of rod- or cone-characteristic light responses and also the site showing different morphology between rods and cones. For this, we established a method to purify the OS and the inner segment (IS) of rods and also of cones from purified carp rods and cones, respectively, using sucrose density gradient. In particular, we were interested in proteins tightly bound to the membranes of cone OS. To identify these proteins, we analyzed proteins in some selected regions of an SDS-gel of washed membranes of the OS and the IS obtained from both rods and cones, with Liquid Chromatography-tandem Mass Spectrometry (LC-MS/MS) using a protein database constructed from carp retina. By comparing the lists of the proteins found in the OS and the IS of both rods and cones, we found some proteins present in cone OS membranes specifically or dominantly, in addition to the proteins already known to be present specifically in cone OS.

## Introduction

In the vertebrate retina, there are two types of photoreceptors, rods and cones. Both photoreceptors consist of four regions: outer segment (OS), inner segment (IS), cell body and synaptic terminal. The OS is the specialized region to convert light signals into electrical signals. For this, OS contains a molecular machinery to generate a light response. This machinery, phototransduction cascade, has been studied extensively in rods, and now rod phototransduction is one of the well-known trimeric G-protein-coupled signaling pathways (see [1–3] for reviews). For cones, it is known that proteins homologous or identical to those expressed in rods are expressed, and thus homologous signaling pathway is present in cones [2, 3].

Rods and cones show different characteristics in light responses [2, 3]. Light sensitivity is higher in rods than in cones. In addition, light responses are much briefer in cones than in rods. These differences underlie the difference of our vision in the dark and light. One possibility of these differences is that activities and/or expression levels of phototransduction proteins are different between rods and cones. Carp (*Cyprinus carpio*) is so far the only animal from which we can obtain purified cones in a quantity large enough to do biochemical studies [4]. Using these purified carp rods and cones, we have been testing the above possibility, and found that the signal amplification is lower, and termination of each reaction is much faster in cones than in rods [5–8]. These findings so far explain qualitatively the differences in light responses between rods and cones.

However, it is still possible that proteins other than known ones are present in rod OS (ROS) or cone OS (COS) and contribute to the differences in the response characteristics between rods and cones. In addition, there may be ROS- or COS-specific protein(s) that contribute to other differences between rods and cones, for example, morphological difference in the OS shape between rods and cones. To examine these possibilities, it would be most effective to compare proteins expressed in ROS and those in COS. The method to obtain purified ROS has been known for many years, but for COS, its purification method has not been known, mainly because of difficulties in obtaining purified cones in a quantity large enough to manipulate. In the present study, first using sucrose density gradient, we prepared purified COS from purified carp cones. We also prepared purified ROS from purified carp rods. In our purified rods and cones in carp, the IS consisting of ellipsoid plus myoid and the OS are both preserved, so that we can also obtain purified IS.

In the present study, as a first step of identification of proteins responsible for rod and cone differences, we focused on proteins tightly bound to COS membranes, so that we prepared COS membranes washed intensively (washed COS membranes). We also obtained ROS membranes, rod inner segment (RIS) membranes and cone inner segment (CIS) membranes similarly. To identify proteins tightly bound to COS membranes, we analyzed proteins in the regions outside of visual pigment bands in the SDS-PAGE gel of the washed ROS, RIS, COS and CIS membranes with Liquid Chromatography-tandem Mass Spectrometry (LC-MS/MS) using a protein database constructed from carp retina. By comparing the lists of the proteins found in the above four kinds of membranes, we tried to find proteins expressed specifically or dominantly in COS membranes (COS-specific/dominant proteins). Although our analysis was made not in the whole region of the molecular masses of proteins in a gel, we found that overall, protein expression profiles are similar between ROS and COS in carp under the conditions employed in this study.

## Materials and methods

### Ethics statement

All experiments with carp (*Cyprinus carpio*) and with mice in this study were performed in accordance with the Osaka University Guidelines for the Welfare and Use of Laboratory Animals, and approved by the Committee on Animal Care of the Graduate School of Frontier Biosciences of Osaka University (approval number FBS-15-002 for carp used to obtain purified rods and cones, and FBS-12-009 for mice used to raise the antiserum against carp calnexin). Mice were sacrificed by cervical dislocation, and carp first by stunning blow to the head followed by pithing.

### Chemicals and antibodies

All chemical reagents were purchased from Nacalai Tesque (Kyoto, Japan). Monoclonal anti-chicken Na^+^/K^+^ ATPase α subunit antibody (a5) was obtained from Developmental Studies Hybridoma Bank (Univ. Iowa, USA). Anti-human TOM20 antibody (sc-11415) was obtained from Santa Cruz Biotechnology (Texas, USA). Anti-human neurocalcin δ antibody (NBP2-15037) was obtained from Novus Biologicals (Colorado, USA). Polyclonal anti-carp mitochondrial aspartate aminotransferase 2 (mAAT; GenBank ID, AB793727.1) antiserum was raised against its peptide fragment (Ser28-Lys428) in mice by Mr. Komatsu in our lab.

Polyclonal anti-calnexin antiserum was prepared as follows. Partial nucleotide sequence corresponding to Ile506—Lys593 in carp calnexin (AB894402.1) was inserted into the BamHI/XhoI sites of an expression vector, pGEX-5X-1 (GE Healthcare, Chicago, USA), to obtain N-terminal Glutathione S-transferase (GST) fusion peptide of calnexin. It was expressed in E. coli BL21DE3 (Merck Millipore, Massachusetts, USA) and purified using a glutathione resin column according to the manufacturer's instruction. Then, mice were immunized with the purified fusion protein. The antiserum obtained from mice was confirmed to react specifically to the calnexin peptide, Ile506—Lys593, in its N-terminal maltose-binding protein (MBP) fusion form.

Peroxidase-conjugated anti-mouse IgG antibody and anti-rabbit IgG antibody were obtained from Kirkegaard & Perry Laboratories (Maryland, USA). The secondary antibodies, Alexa488-conjugated anti-rabbit IgG antibody and Alexa568-conjugated anti-mouse IgG antibody, were obtained from Thermo Fisher Scientific (Massachusetts, USA).

### Preparation of purified carp rods and cones

Rod and cone photoreceptors were purified from carp retina as described [4]. Briefly, rods and cones were mechanically dissociated from isolated carp retina by tapping the retina using a paintbrush in Ringer's solution (119.9 mM NaCl, 2.6 mM KCl, 0.5 mM CaCl_2_, 0.5 mM MgCl_2_, 0.5 mM MgSO_4_, 1mM NaHCO_3_, 16 mM glucose, 0.5 mM NaH_2_PO_4_, 4 mM HEPES, pH 7.5). Each of dissociated cells retained the OS and the IS consisting of ellipsoid plus myoid, but lacked cell body and synaptic terminal [2]. Then, the rods and cones were separated using Percoll stepwise density gradient. Purified rods and cones thus obtained were washed twice with a potassium gluconate buffer (K-gluc buffer; 115 mM potassium gluconate, 2.5 mM KCl, 2 mM MgCl_2_, 0.1 mM CaCl_2_, 0.2 mM EGTA, 1 mM dithiothreitol, 10 mM HEPES, pH7.5). With this wash, most of contaminated red blood cells, but not rods or cones, were lysed and their soluble proteins were mostly removed in the supernatant. The purified cells were resuspended in Ringer's solution. Our previous estimation of the contamination of cones in the purified rods and that of rods in the purified cones were both <1% [4].

### Separation of OS membranes from IS membranes in purified cells

Purified rods were suspended in Ringer’s solution and purified cones in 2 × Ringer’s solution. (For unknown reasons, separation of OS from IS in cones was more effective when 2 × Ringer’s solution was used). Then, the suspension (~800 μL) was passed through a 27-gauge needle 15 times using a 1 mL syringe to mechanically dissociate OS from IS for both rods and cones. During this procedure, cytoplasmic proteins were probably eluted out from the OS or the IS. The resultant suspension of a mixture of separated OS and IS membranes of rods or cones were then placed on top of a stepwise sucrose density gradient in Ringer's solution in a test tube, and centrifuged for an hour at 190,000 × g at 4°C. With the above procedure, we obtained a fraction rich of ROS membranes and that of RIS membranes from purified rods, and those rich of COS and CIS membranes from purified cones (see Results and discussion).

In the present study, we were interested in the proteins tightly bound to membranes. Therefore, when necessary, membranes were washed intensively by centrifugation (190,000 × g, 10 min at 4°C) with a low salt buffer (4 mM HEPES, 1 mM EDTA, pH 7.5) twice and subsequently with a high pH buffer (100 mM Na_2_CO_3_, pH 11.5) four times to remove soluble and membrane-associated peripheral proteins. These washed membranes were used for LC-MS/MS proteomic analysis (see Results and discussion).

### Quantitative evaluation of contamination of IS or OS membranes

To evaluate the purity of membranes separated with sucrose density gradient centrifugation described above, OS- or IS-specific marker proteins were quantified in each separated membrane fraction. A marker of OS membranes, visual pigments, was quantified spectrophotometrically as described [4, 9, 10]. As the markers of membranes in the IS, F1 ATPase β subunit was used for mitochondrial inner membranes, TOM20 for mitochondrial outer membranes, calnexin for endoplasmic reticulum (ER) membranes, and Na^+^/K^+^ ATPase α subunit for IS plasma membranes. The amount of F1 ATPase β subunit was quantified by fluorescent staining with Oriole (Bio-Rad Laboratories, California, USA) after SDS-PAGE using bovine serum albumin (BSA) as a molar standard. The amounts of Na^+^/K^+^ ATPase α subunit and those of calnexin and TOM20 were measured by quantitative immunoblot analysis as described [5].

### Construction of a database of proteins expressed in carp retina

For the comparative proteomic analysis of rod and cone proteins in the OS and the IS membranes, a database of proteins expressed in carp retina was constructed as described below. Retinal total RNA was extracted from carp retina using TRI reagent (Sigma-Aldrich, California, USA) according to the manufacturer’s protocol. Subsequent RNA quality check, RNA sequence library preparation and RNA sequencing were performed at Hokkaido System Science (Hokkaido, Japan), and raw sequence data were submitted to DDBJ Sequence Read Archive (DRA) under accession number, DRA004555 (Bioproject ID: PRJDB4664). Then, the acquired sequences were assembled as contigs by a de novo RNA-seq data assembly method at Hokkaido System Science using a software, Trinity (version r20130225, k-mer 25). The assembled 258,867 contigs were BLASTX-searched against NCBI vertebrate_other protein database (release 69, E-value 1e-5) using BLAST+ (version 2.2.29+). A total of 124,947 contigs hit with this search was each annotated with the top hit NCBI gene name and the species name. The contigs that were not identified by the BLAST search (133,920 out of 258,867 contigs) were almostly short ones and removed from the analysis hereafter. The annotated contigs were translated from DNA sequences to amino acid sequences using TransDecoder (version r20140704). As a result, 125,175 amino acid sequences were obtained. The increase in the number of amino acid sequences from that of contigs was caused by the presence of contigs that have multiple start codon candidates. The number of amino acid sequences was more than that of all carp genes, 52,610, reported previously [11], due to the presence of splicing variants plus polymorphism mainly in UTR regions and partially in coding regions. DNA sequences coding the same amino acid but having different DNA sequences in the UTR region were not merged at this stage. Then, using acquired data of the sequences, a sequence database of carp retinal proteins was constructed [12], and used for the following proteomic analysis.

### Liquid Chromatography-tandem Mass Spectrometry (LC-MS/MS) analysis

Washed membranes containing tightly membrane-bound proteins mainly were dissolved in SDS-PAGE sample buffer (62.5 mM Tris, 10% (w/v) glycerol, 100 mM dithiothreitol, 2.3% SDS, 0.01% (w/v) bromophenol blue), and kept on ice >1 hour. After solubilization, membranes were subjected to SDS-PAGE, and gels were silver-stained. Then, the proteins other than those in the visual pigment bands in the gel were digested by in-gel tryptic cleavage, and resultant peptides were extracted from the gel [13]. The peptides were subjected to shotgun proteomic analysis using QTRAP^®^5500 LC-MS/MS System (AB SCIEX) and Mascot software (version 2.4, Matrix Science, Boston, USA).

### Immunological analysis on the localization of neurocalcin δ B in cones

To investigate the subcellular localization of neurocalcin δ B (NCALD) in cones, immunocytochemical analysis was made in isolated cones as described [14] with some modifications. Briefly, mechanically isolated carp photoreceptors were suspended in Ringer’s solution supplemented with 4% (w/v) paraformaldehyde, and then incubated for 12 hours at 4°C in the dark. Then, they were mounted on a silane-coated slide glass and permeabilized with phosphate buffered saline (PBS; 137 mM NaCl, 2.7 mM KCl, 8.1 mM Na_2_HPO_4_, 1.5 mM NaH_2_PO_4_, pH 7.4) containing 1% (w/v) Triton X-100 for 5 min. Mounted samples were air-dried and blocked with PBS containing 0.2% (w/v) Triton X-100 and 1.5% (v/v) normal goat serum. Mounted cells were then washed with PBS for three times, and incubated for an hour at room temperature with a solution containing anti-human NCALD rabbit antibody (at 1:75 dilution) and anti-carp mAAT mouse antiserum (at 1:300 dilution). Then the cells were washed with PBS three times, and incubated at room temperature for 30 min with a mixture of two secondary labeled antibodies, Alexa488-conjugated anti-rabbit IgG (1:200 dilution) for NCALD and Alexa568-conjugated anti-mouse IgG (1:300 dilution) for mAAT.

## Results and discussion

### Purity of each of OS and IS membranes

**Fig 1:**
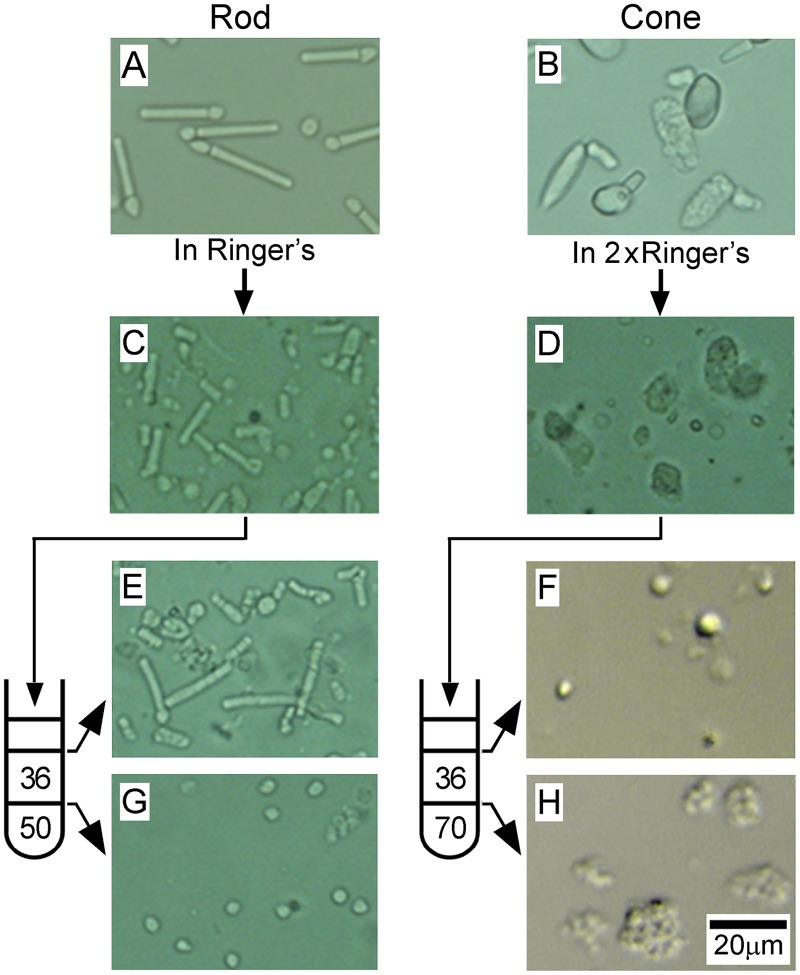
Purification of OS and IS membranes from purified rods and cones. Differential interference contrast microscopic (DIC) images of the cell fractions in each step of the purification are shown. Purified carp rods (A) and cones (B) were passed through a 27-gauge needle to dissociate the OS from the IS, and the resultant broken rods (C) and cones (D) were layered on a sucrose density gradient made in a test tube (drawings in the left of E/G and F/H) to centrifuge. The number in the drawings shows the density of sucrose (%, w/v). Separated membranes at upper (E, F) and lower (G, H) interfaces were collected. Scale bar, 20 μm throughout.

In this study, we originally intended to identify soluble and membrane-bound proteins expressed specifically or dominantly in cone OS. It would be ideal to detect proteins expressed in purified cone OS and compare them with proteins expressed in cone IS, rod OS and rod IS. For this purpose, we first tried to isolate intact OS containing soluble and membrane-bound proteins from purified carp rods with the mechanical method reported previously for frog rod OS showing typical rod-shape [15]. However, our attempt was not successful because we observed only deteriorated rod OS in carp (see Fig 1E, for example). Thus, in this study, we obtained OS membranes, instead of intact OS, from purified carp rods and cones. The procedure we took is shown in Fig 1. Purified rods (Fig 1A) and cones (Fig 1B) were mechanically disrupted to separate the OS and the IS (Fig 1C and 1D) (see Materials and methods). A mixture of deteriorated and separated OS and IS was placed on top of a stepwise sucrose density gradient, 36/50% (w/v) for rods and 36/70% (w/v) for cones, in a test tube, and then centrifuged to separate them (Fig 1E and 1G for rods, and Fig 1F and 1H for cones). At this stage, OS and IS were disrupted and they were practically in the form of membranes mainly containing membrane-bound proteins. These membranes in each interface were collected and suspended in Ringer's solution.

To evaluate how effectively the separation of OS and IS membranes was achieved with this procedure, membranes obtained at the upper interface (Fig 1E for rods and Fig 1F for cones) and those at the lower interface (Fig 1G for rods and Fig 1H for cones) were probed quantitatively with marker proteins.

**Fig 2:**
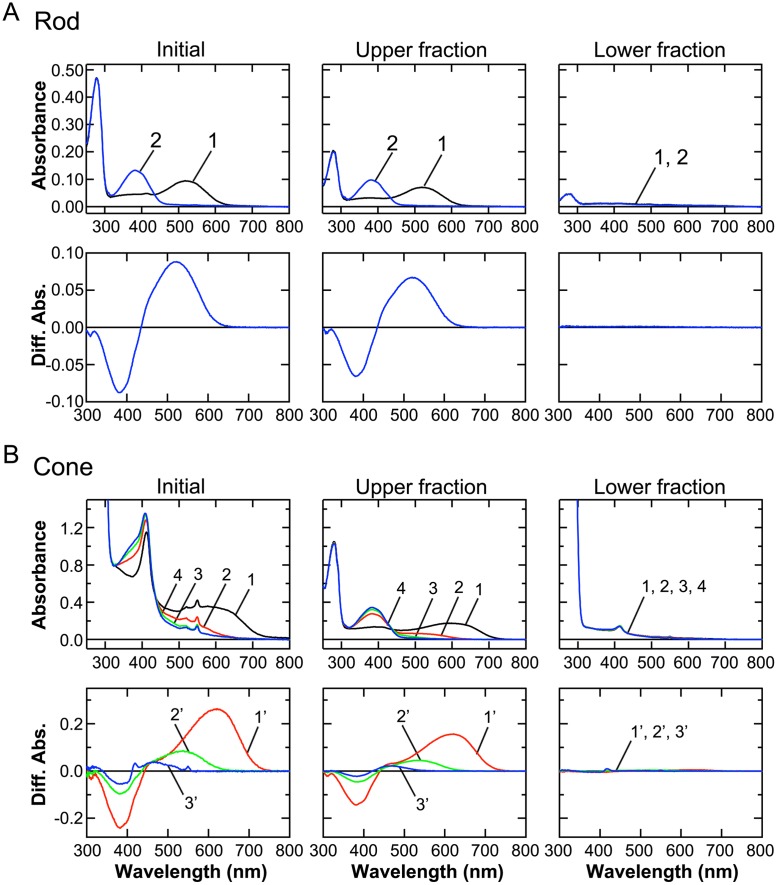
Quantification of visual pigments. Quantity of visual pigments was measured spectrophotometically in three types of rod (A) and cone (B) preparations: membranes from purified cells as initial materials (Initial), membranes in the upper (Upper fraction) and lower (Lower fraction) fraction. (A) Rhodopsin content was measured in the initial rod membranes (left panels), in the upper and lower fraction (middle and right panels, respectively), all obtained from the same number of cells and suspended in the same volume of Ringer's solution. In each of upper panels, curve 1 (black) shows the absorption spectrum before bleach, and curve 2 (blue) shows the spectrum after complete bleach of rhodopsin with illumination of >440 nm light. Curve 2 was subtracted from curve 1 in each of the upper panel to obtain a difference spectrum, which is shown in the corresponding lower panel. From positive absorption by rhodopsin (λ_max_ = 522 nm), relative rhodopsin content was determined. (B) Contents of red-, green-, and blue-sensitive pigments were measured in the initial purified cone membranes (left panels), in the upper and lower faction ((middle and right panels, respectively). In each of upper panels, curve 1 (black) shows absorption spectrum before bleach. Red-sensitive pigment was first bleached with >675 nm light (curve 2), and then green-sensitive pigment with >600 nm light (curve 3) and finally blue-sensitive pigment with >440 nm light (curve 4). Curve 2 was subtracted from curve 1 to obtain a difference spectrum of red-sensitive pigment, which is shown in the corresponding lower panel (red curve 1', λ_max_ = 622 nm). Similarly, difference spectra were obtained for green-sensitive pigment (green curve 2', i.e., curve 2 –curve 3; λ_max_ = 535 nm) and for blue-sensitive pigment (blue curve 3', i.e., curve 3 –curve 4; λ_max_ = 460 nm) to determine the relative contents of these pigments.

**Fig 3:**
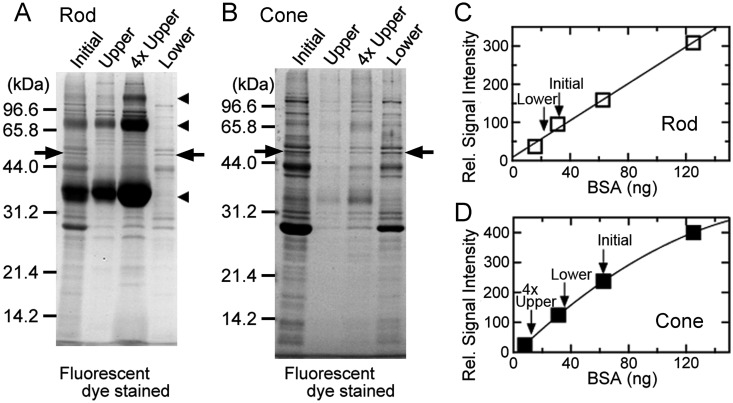
Quantification of F1 ATPase β subunit. Quantities of F1 ATPase β subunit were determined by SDS-PAGE in four rod (A) and four cone (B) membrane preparations. (A) Rod membranes as initial materials (Initial), and rod upper and rod lower fractions (Upper and Lower, respectively) were subjected to SDS-PAGE. The gels were stained with a fluorescent dye, Oriole. In the lane labeled as 4×Upper, 4 times volume of the upper fraction was applied to quantify the amount of F1 ATPase β subunit precisely. Arrowheads indicate the monomer, dimer and tetramer bands of rhodopsin, and arrows indicate the band of F1 ATPase β subunit. (B) Similar SDS-PAGE pattern using four cone membrane preparations. (C) An example of quantification of F1 ATPase β subunit with Oriole staining in four rod membrane preparations. A calibration curve was obtained with Oriole staining using known amounts of BSA (open rectangles and filled line), which was performed in parallel with SDS-PAGE of the rod membrane preparations. From the signal intensity of F1 ATPase β subunit in (A), the amount of F1 ATPase β subunit was quantified in four rod membrane preparations using the calibration curve (downward arrows). Note that F1 ATPase β subunit was not detected in upper fractions in (A). (D) Similar quantification in four cone membrane preparations.

Visual pigments were used as the OS marker protein. Their contents in the membranes of starting purified rods and cones, and those in the four fractions obtained from upper (arrows in Fig 1E and 1F) and lower interfaces (arrows in Fig 1G and 1H), were quantified spectrophotometrically (Fig 2). Membranes obtained at each interface were suspended in Ringer's solution of the volume same as that of the initial total membranes obtained from purified rods or cones. In the followings, we describe total membranes obtained from starting purified rods or cones as just initial rod or cone membranes, and indicate the content of a protein in each upper or lower fraction as the % of the content in the initial membranes. In the case of rods, rod visual pigment, rhodopsin, was recovered almost exclusively in the upper fraction (Fig 2A and Table 1). When the initial rod membranes and membranes in the upper fraction of rods were subjected to SDS-PAGE (Fig 3A), thick rhodopsin bands (arrowheads) were detected (Initial and Upper in Fig 3A, respectively). In contrast, in the lower fraction (Lower fraction in Fig 2A and Lower in Fig 3A), rhodopsin was practically not detected. All of the results shown in Figs 2A and 3A showed that ROS membranes were collected in the upper fraction almost exclusively.

**Table 1:** Recovery of membranes after separation of OS and IS.

Localization	Outer segment membrane	Inner segment, Mitochondrial membranes		Inner segment, Plasma membranes	Inner segment, ER membranes
		Inner membranes	Outer membranes		
Protein	Visual pigment	F1 ATPase β subunit	TOM20	Na^+^/K^+^ ATPase α subunit	Calnexin
**Rod upper fraction**	86.2 ± 13.4%	N.D.	9.1 ± 0.4%.	65.7 ± 9.5%	26.5 ± 4.3%
**Rod lower fraction**	0.9 ± 0.2%	84.8 ± 4.3%	42.5 ± 7.0%	40.2 ± 1.2%	9.9 ± 3.1%
**Cone upper fraction**	54.5 ± 3.2%	3.5 ± 0.9%	6.86 ± 1.5%	49.0 ± 8.0%	35.5 ± 3.1%
**Cone lower fraction**	3.5 ± 0.2%	54.6 ± 4.4%	58.2 ± 12.3%	10.9 ± 1.6%	38.4 ± 5.3%

Each value is shown as mean ±S.E in 3 independent measurements. N.D., signals not detected.

In the case of cones, red-, green-, and blue-sensitive cone visual pigments were dominantly detected in the membranes in the upper fraction (Fig 2B, left and middle panels) as in the case of rods. The amount of cone visual pigments in the lower fraction was almost negligible similarly as in rods (Fig 2B, right panels). These results clearly show that both rod and cone OS membranes were collected effectively at the upper interface (Ringer's solution/36% sucrose interface) and therefore in the upper fraction, and there were little contamination of OS membranes in the lower fractions.

We next examined separation of IS membranes. First, separation of inner mitochondrial membranes was examined by quantifying its marker protein, F1 ATPase β subunit [16], in the upper and the lower fractions of both rods and cones. When the initial rod membranes and membranes in the upper and lower fractions of rods (Fig 3A), or those of cones (Fig 3B) were subjected to SDS-PAGE, a band was detected at 55 kDa (arrows) in the lane of initial rod membranes (Fig 3A, Initial) and that of initial cone membranes (Fig 3B, Initial). This band was identified as carp F1 ATPase β subunit (AB023582.1) by our mass spectrometry, and was also clearly detected in the lower fractions of both rods and cones (Lower in Fig 3A and 3B, respectively). In contrast, this band was faint in the lanes of upper fractions of both rods and cones (Upper in Fig 3A and 3B, respectively). The amount of F1 ATPase β subunit in each of the fractions (Initial, Upper, 4×Upper and Lower) was quantified by measuring the Oriole fluorescent signals of the band in each lane using BSA as a molar standard (Fig 3C and 3D), and the results showed that the mitochondrial inner membrane marker was detected minimally in the upper fraction (not detected in rods and 3.5% in cones) and mainly in the lower fraction (84.8% in rods and 54.6% in cones) (Table 1, F1 ATPase β subunit).

**Fig 4:**
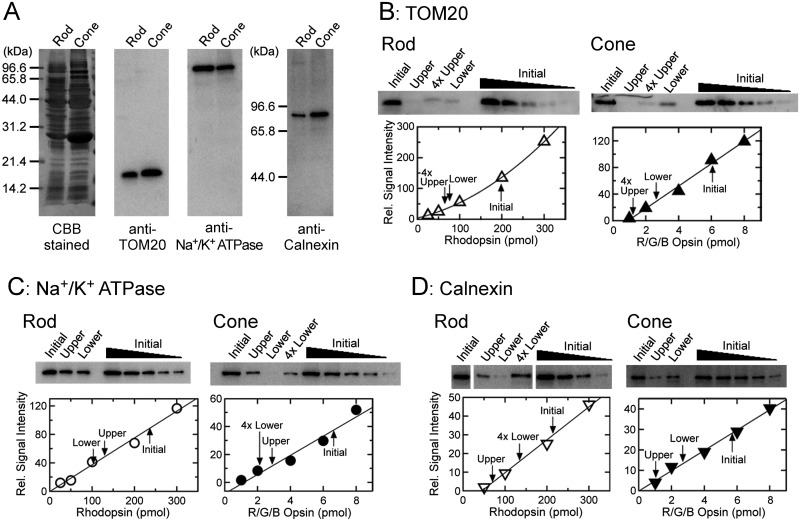
Estimation of separation of OS and IS membranes using TOM20, Na^+^/K^+^ ATPase α subunit and calnexin as marker proteins. (A) Specificity of antibodies used to detect TOM20, Na^+^/K^+^ ATPase α subunit and that of anti-calnexin antiserum. Purified rod membranes containing 200 pmol of rhodopsin and cone membranes containing 6 pmol of cone total pigments were subjected to SDS-PAGE and were stained with Coomassie Brilliant Blue (left panel) or probed with antibodies or antiserum against each protein (right three panels). (B-D) Quantitative immunoblot analyses of TOM20 (B), Na^+^/K^+^ ATPase α subunit (C) and calnexin (D). In the upper panels in each of (B)—(D), purified rod membranes containing 200 pmol of rhodopsin or purified cone membranes containing 6 pmol of total cone pigments (Initial), upper and lower membrane fractions obtained from the same number of the purified cells (Upper and Lower, respectively), and a diluted series of initial rod and cone membranes were subjected to SDS-PAGE. These membranes were probed with antibodies or antiserum against each marker protein. To detect the amounts of target proteins precisely, 4 times volume of samples were applied when necessary (4×). The amount of a target protein in each of the membranes was determined with a calibration line obtained from immunoblot signals obtained in a diluted series of initial rod or cone membranes. In the lower panels in each of (B)—(D), examples of quantification are shown. The quantity of a target protein in each fraction is indicated by an arrow in lower panels. With this estimation, one can determine how much % of the target protein is present in each of the membranes as compared with the amount in the initial rod or cone membranes of the same cell number.

We then identified in which fraction, upper or lower, the membranes of other cellular organelles in the IS were present. Thus, those membranes in these fractions were probed with quantitative immunoblot analyses using three marker proteins: TOM20 for mitochondrial outer membranes [17, 18], Na^+^/K^+^ ATPase α subunit for IS plasma membranes [19, 20] and calnexin for ER membranes [21, 22] (Fig 4). As shown in Fig 4A, each of antibodies or antiserum used in the analysis detected a single band in purified rod and cone membranes, and the molecular mass of each of the detected band corresponded to the known value of the target protein. These results indicated that used antibodies and antiserum reacted to target proteins selectively.

Then, the three marker proteins were quantified in initial rod and cone membranes and in the upper and the lower fractions (Fig 4B–4D and Table 1). Fig 4B shows an example of the quantification of TOM20, a marker of mitochondrial outer membranes. This marker was detected in the upper fraction to some extent (9.1% in rods and 6.9% in cones), but much more in the lower fraction (42.5% in rods and 58.2% in cones) similarly as the mitochondrial inner membrane marker, F1 ATPase β subunit.

The IS plasma membrane marker, Na^+^/K^+^ ATPase α subunit, was detected in both the upper and the lower fractions, but the marker was present slightly more in the upper fraction: the percentage of the marker present in the upper fractions was 65.7% in rods and 49.0% in cones, and in the lower fractions, it was 40.2% in rods and 10.9% in cones (Fig 4C and Table 1). Calnexin, an ER membrane marker, was also present in both the upper and the lower fraction as IS plasma membranes. This marker was slightly enriched in the upper fraction in rods (26.5% in the upper fraction and 9.9% in the lower fraction) but detected almost equally in both fractions in cones (35.5% in the upper fraction and 38.4% in the lower fraction) (Fig 4D and Table 1).

From these results, it is concluded that, with our separation method, upper fraction exclusively contains OS membranes together with some of IS membranes (mitochondrial inner and outer membranes, IS plasma membranes plus ER membranes) and lower fraction contains mainly mitochondrial membranes and some of the IS plasma membranes and ER membranes. For convenience, we call upper fraction as OS-rich fraction and lower fraction as IS-rich fraction. To distinguish each fraction obtained from rods or cones, we call rod OS-rich fraction as ROS-rich fraction. Similarly we will use the terms of RIS-rich fraction (rod IS-rich fraction), COS-rich fraction and CIS-rich fraction in the followings.

Although the OS-rich fraction contains OS membranes exclusively, it also contains some of the IS membranes: IS plasma membranes, ER membranes and less abundantly mitochondrial inner and outer membranes. The membranes we used are all from our purified rods or cones. These purified cells contain OS and also IS consisting of the ellipsoid and the myoid but lacking the nucleus and the terminal (see Fig 1 in [2]). From consideration of the structural basis of our purified rods and cones, IS plasma membranes and ER membranes are probably present much less than mitochondrial membranes and also much less than membranes of highly membranous OS. For this reason, contamination of IS plasma membranes and that of ER membranes would be limited in our OS-rich fraction of both rods and cones.

In the present study, we focused on the COS-specific/dominant proteins. Our estimation of the amount of a marker protein shown above was based on the percentage of the proteins found in each of the membrane fractions comparing with that in the initial rod or cone membranes. In the COS-rich fraction, 3.5–6.9% contamination of CIS proteins was observed (Table 1). Note that this contamination is the percentage of the total CIS proteins, and therefore, the actual contamination of CIS proteins is dependent on the total CIS proteins and total COS proteins. Our previous estimation of the volumes of COS and CIS in carp showed that the IS volume is ~6 times higher than the OS volume [10]. Based on this volume ratio, we estimated the actual contamination of CIS proteins in COS-rich fraction. We assume an extreme case that the membrane density and protein density in the membranes are the same in COS and CIS. In this case, based on the distribution of visual pigment (~55% of pigments in the initial COS membranes) and those of CIS proteins (TOM20 and F1 ATPase β subunit; 3.5–6.9% in the initial CIS membranes) in COS-rich fraction shown in Table 1, our calculation showed that the amount of the contaminated CIS proteins, mainly those in the mitochondrial inner and outer membranes, could be equivalent to the amount of the COS proteins in the COS-rich fraction (COS proteins: CIS proteins = ~55% × 1: 3.5–6.9% × 6). On the contrary, COS protein contamination in the CIS-rich fraction could be negligible (COS proteins: CIS proteins = 3.5% × 1: 54.6–58.2% × 6). Because contamination of CIS proteins in the COS-rich fraction cannot be ignored, we identified COS-specific/dominant proteins by excluding the proteins found in CIS-rich fraction which was contaminated with COS membranes minimally. We also excluded the proteins found in ROS-rich fraction (see below).

### Identification of COS-specific/dominant proteins

To identify COS-specific/dominant proteins, we made a list of proteins detected in each of the ROS-rich, RIS-rich, COS-rich, and CIS-rich fractions using shotgun proteomic analysis by LC-MS/MS. COS-rich fraction contains contaminated CIS membranes significantly. It is also contaminated with ROS and RIS membranes and red blood cell membranes, which are all present potentially in our purified cones in a small amount [4]. Then, to find COS-specific/dominant proteins, the proteins found in CIS-rich and ROS-rich fractions significantly were excluded from the list of the proteins detected in the COS-rich fraction. With this comparison, not only the common proteins found in both ROS-rich and COS-rich fractions but also the CIS proteins contaminated or commonly present in COS-rich fraction can be excluded.

**Fig 5:**
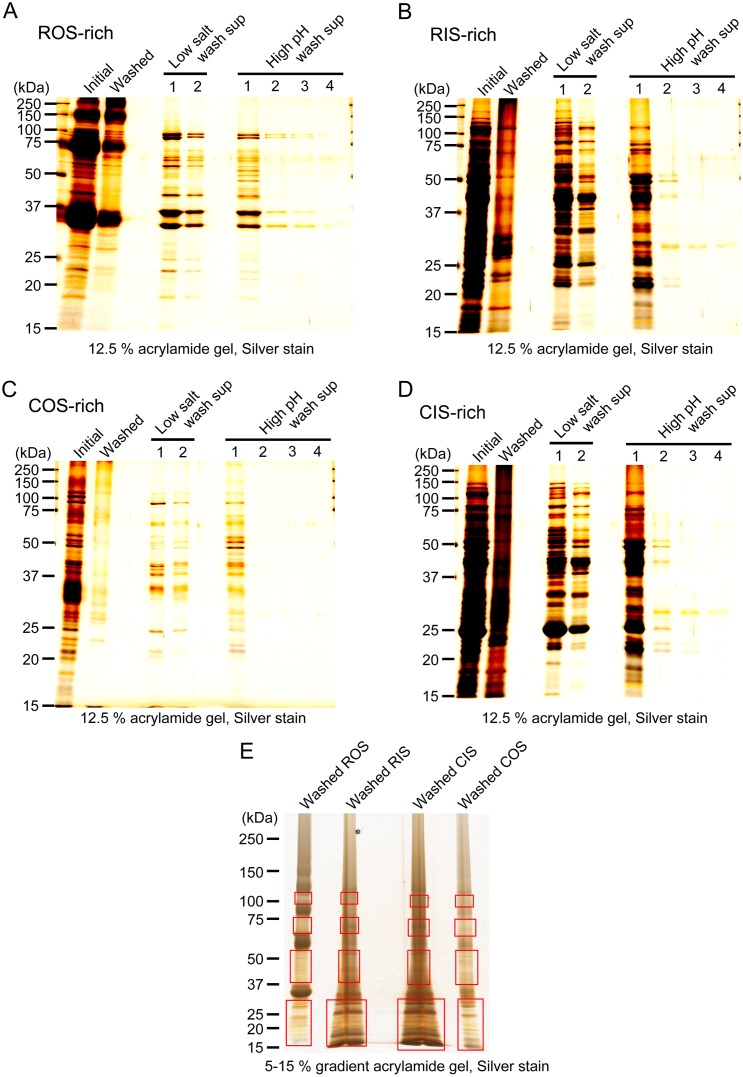
Preparation of washed membranes for LC-MS/MS. Membranes in ROS-rich (A), RIS-rich (B), COS-rich (C) and CIS-rich (D) fractions were intensively washed with a low salt buffer and a high pH buffer to eliminate soluble and peripheral membrane proteins as much as possible. In (A)—(D), SDS-PAGE patterns of the membranes prepared from initial membranes (Initial), the membranes finally obtained after intensive washes (Washed), and supernatants obtained during washes (Low salt wash sup 1–2 and High pH wash sup 1–4) are shown for membranes prepared from each fraction. In (E), an example of a gel subjected to LC-MS/MS analysis is shown. Washed membranes obtained from ROS-, RIS-, COS-, and CIS-rich fractions were subjected to SDS-PAGE, and boxed areas of each lane were cut out of the gel and subjected to in gel digestion for LC-MS/MS analysis. Membranes used for SDS-PAGE in each of the lane in (E) were obtained from 10^6^ rods (Washed ROS), 4.7 × 10^5^ rods (Washed RIS), 1.2 × 10^5^ cones (Washed CIS) and 2.5 × 10^4^ cones (Washed COS).

In this study, we focused on the proteins tightly bound to membranes as the first step to identify COS-specific/dominant proteins. For this purpose, membranes in each of the ROS-rich, RIS-rich, COS-rich and CIS-rich fractions were washed to remove soluble and membrane-associated peripheral proteins as much as possible (see Materials and methods). SDS-PAGE patterns of the membranes in each of the fractions before wash (Initial, in Fig 5A for example) and the final washed membranes (Washed) are shown in Fig 5A–5D for ROS-rich, RIS-rich, COS-rich and CIS-rich fractions, respectively. SDS-PAGE patterns of the supernatants after washes with low salt buffer twice and with high pH buffer four times (Low salt wash sup and High pH wash sup, respectively) showed that most of the removable proteins were washed away from the membranes.

These washed membranes in each of the fractions shown in Fig 5A–5D were then subjected to SDS-PAGE to separate proteins for the analysis with LC-MS/MS. In the ROS-rich fraction, visual pigment, rhodopsin, is the most abundant protein (approx. 95%). Proteins separated in the gel were digested for the LC-MA/MS analysis, but massive amount of rhodopsin probably impedes the LC-MS/MS analysis by masking other proteolytic products when it is included. For this reason, rhodopsin bands, monomer at 30~40 kDa, dimer at 55~65 kDa and trimer at 80–90 kDa, were excluded in our present LC-MS/MS analysis. In the analyses of proteins in other fractions, the same regions were omitted in order to compare the proteins equally. Therefore, proteins present in the excluded regions were not analyzed and proteins present close to these excluded regions could not be analyzed quantitatively. The regions indicated by four rectangles in each lane in Fig 5E were subjected to proteolysis and analyzed with LC-MS/MS. As a result, we identified 648, 971, 1,064 and 629 proteins from washed ROS-rich, RIS-rich, CIS-rich and COS-rich fraction, respectively, as total proteins obtained from two independent experiments (S1–S4 Tables).

Then, we identified COS-specific/dominant proteins in the COS-rich fraction (Table 2). For this purpose, we used the emPAI (exponentially modified protein abundance index) value, which has been reported to be proportional to the amount of a protein present in a sample used for LC-MS/MS analysis [23]. Briefly, we used emPAI values obtained from the same number of the cells as an index of the amount of a protein present in the washed membranes. We calculated the portion of a protein in the COS-rich fraction in a total amount of that protein in COS-rich and CIS-rich fractions (shown as COS/(COS+CIS) in Table 2). With this procedure, we excluded proteins present more in CIS-rich fraction (i. e., those proteins showing COS/(COS+CIS)<0.5). We also excluded proteins present in ROS-rich fraction in a quantity similar to that in COS-rich fraction (see Methods in the legend to Table 2). After this manipulation, 48 proteins remained in the list. Note that ideally, but not actually, visual pigments are not present in Table 2 or in S1–S4 Tables, because the bands containing these pigments were not analyzed (see above). Similarly, proteins present in the rhodopsin bands were not analyzed, and those present at the region close to these bands were not analyzed correctly. Among the 48 proteins, 8 proteins are already known to be present only in COS (shown in bold in Table 2), and this fact ensures that our strategy to identify COS-specific/dominant proteins is valid. However, 16 proteins that are known to be localized outside of COS also remained in the list (proteins with grey background in Table 2). The remaining 24 proteins without special indication in Table 2 could be specifically or dominantly separated into the COS-rich fraction during our membrane separation.

**Table 2:** List of probable proteins present almost exclusively in COS-rich fraction.

Protein*	Molecular Mass	emPAI		COS
		COS-rich	CIS-rich	(COS+CIS)
^a^creatine kinase b-type [24]	16 kDa	1.92	0	1
ADP-ribosylation factor 1	21 kDa	1.34	0	1
^b1^protein RD3 [25]	15 kDa	1.04	0	1
^a^retinoschisin precursor [26]	22 kDa	0.62	0	1
PREDICTED: guanine nucleotide-binding protein G(i) subunit alpha-1	40 kDa	0.56	0	1
PREDICTED: guanine nucleotide-binding protein subunit alpha-13-like	34 kDa	0.52	0	1
PREDICTED: gamma-glutamyltransferase 5 isoform X1	62 kDa	0.5	0	1
**PREDICTED: cyclic nucleotide-gated cation channel beta-3-like isoform X2 [**27**]**	**28 kDa**	**0.47**	**0**	**1**
^c^carbonic anhydrase [28]	29 kDa	0.45	0	1
PREDICTED: cadherin-related family member 5-like isoform X2	20 kDa	0.41	0	1
neurocalcin-delta B	22 kDa	0.38	0	1
**PREDICTED: cyclic nucleotide-gated channel cone photoreceptor subunit alpha isoform X2 [**27**]**	**81 kDa**	**0.36**	**0**	**1**
PREDICTED: flotillin 1a isoform X1 [29]	47 kDa	0.35	0	1
ras-related protein Rab-43	23 kDa	0.35	0	1
^d^vitamin K epoxide reductase complex subunit 1-like protein 1 [30]	12 kDa	0.34	0	1
PREDICTED: ras-related protein Rab-35-like	24 kDa	0.34	0	1
PREDICTED: Fc receptor-like protein 5 isoform X2	12 kDa	0.33	0	1
PREDICTED: SH3-containing GRB2-like protein 3-interacting protein 1 isoform X2	12 kDa	0.32	0	1
synaptic vesicle glycoprotein 2B	12 kDa	0.32	0	1
PREDICTED: uncharacterized protein LOC100334801 isoform X2	26 kDa	0.31	0	1
guanine nucleotide-binding protein G(i) subunit alpha-2	41 kDa	0.3	0	1
ubiquitin-like 3b	13 kDa	0.3	0	1
ras-related GTP-binding protein C	13 kDa	0.29	0	1
^e^band 3 anion transport protein (211V-519E) [31]	35 kDa	3.68	0.08	0.98
^f^PREDICTED: cyclic nucleotide-gated channel rod photoreceptor subunit alpha-like [32]	82 kDa	0.56	0.02	0.974
^e^PREDICTED: ammonium transporter Rh type A isoform X1 [33]	12 kDa	3.2	0.11	0.967
flotillin-1 [29]	41 kDa	2.14	0.1	0.955
^e^band 3 anion transport protein (92M-191C) [31]	11 kDa	5.06	0.27	0.949
flotillin-2a [29]	47 kDa	2.12	0.12	0.947
**guanine nucleotide-binding protein G(t) subunit alpha-2 [**34**]**	**40 kDa**	**28.3**	**2.02**	**0.933**
**opsin-1, short-wave-sensitive 1**	**39 kDa**	**0.44**	**0.03**	**0.933**
^e^band 3 anion transport protein (562G-810W) [31]	27 kDa	1.17	0.1	0.923
^b2^PREDICTED: regulator of G-protein signaling 9-binding protein-like [7, 35]	27 kDa	6.33	0.63	0.909
ignal peptidase complex subunit 3 [36]	20 kDa	1.39	0.14	0.909
^a^PREDICTED: metal transporter CNNM4 [37, 38]	35 kDa	0.66	0.08	0.898
^a^brain creatine kinase b [24]	43 kDa	0.52	0.06	0.895
adipocyte plasma membrane-associated protein	47 kDa	1	0.12	0.893
**green-sensitive opsin-4**	**39 kDa**	**2.04**	**0.25**	**0.892**
^b2^PREDICTED: regulator of G-protein signaling 9 isoform X2 [7]	62 kDa	0.9	0.11	0.889
guanine nucleotide-binding protein G(o) subunit alpha	40 kDa	1.44	0.19	0.885
protein kinase, cAMP-dependent, regulatory, type II, alpha A	45 kDa	0.62	0.09	0.872
synaptotagmin II	47 kDa	0.36	0.06	0.867
**G-protein-coupled receptor kinase 7A [**39**]**	**62 kDa**	**3.49**	**0.56**	**0.863**
^d^dolichyl-diphosphooligosaccharide--protein glycosyltransferase 48 kDa subunit precursor [40]	51 kDa	0.63	0.11	0.854
solute carrier family 2, facilitated glucose transporter member 1	53 kDa	0.61	0.1	0.854
peroxiredoxin-2	22 kDa	0.62	0.13	0.831
**PREDICTED: sodium/potassium/calcium exchanger 2-like isoform X2 [**41**]**	**68 kDa**	**0.45**	**0.1**	**0.816**
**cone cGMP-specific 3',5'-cyclic phosphodiesterase subunit alpha' [**42**]**	**98 kDa**	**0.39**	**0.1**	**0.8**

*Proteins already known to be expressed specifically in COS are indicated in bold, and those known to be expressed in other portions of a cone or in other cells are indicated with grey background:

^a^IS protein,

^b^protein expressed in both ROS and COS^b1^ [24] or abundantly expressed in COS and less abundantly in ROS^b2^ [7, 25],

^c^cone specific protein present both in OS and IS,

^d^ER specific protein,

^e^red blood cell specific protein,

^f^protein identified as a rod protein in zebrafish (100% match in the amino acid sequence in 6 identified peptides), but in carp, identified as cone cGMP gated-channel subunit alpha-like (XP_018956113.1, 99% match) rather than its rod-type (XP_018928039.1, 72% match). The values of emPAI shown are those obtained in the washed membranes of 5 × 10^4^ cones. **Methods**: The amount of a protein analyzed with LC-MS/MS can be quantified with the value of emPAI [23], which is calculated from the number of the observed peptides divided by the number of the observable peptides per protein in the MS analysis and is an index of the amount of each protein in a sample analyzed with LC-MS/MS. Because OS- and IS-rich fractions were contaminated with IS and OS membranes, respectively, we calculated the portion of a protein detected in the COS-rich fraction by dividing the emPAI value of a protein in the OS-rich fraction with the sum of its value in the OS-rich and the CIS-rich fractions (COS/(COS+CIS) in Table 2). Proteins with COS/(COS+CIS) < 0.5, which indicates that the protein is detected more in the CIS-rich fraction, are not listed in Table 2. In addition, proteins also found in ROS-rich fraction are eliminated under the criteria that their emPAI values in ROS-rich fraction, obtained from the same number of rods, i. e., 5 × 10^4^ rods, were more than 1/10 of those in COS-rich fraction. In Table 2, proteins showing COS/(COS+CIS) > 0.8 are shown, based on the fact that many of the known COS-specific proteins are present with COS/(COS+CIS) > 0.8 (see proteins indicated in bold). In addition, those proteins showing emPAI values lower than 1/100 of that of a protein most abundantly detected, i. e., guanine nucleotide-binding protein G(t) subunit alpha-2 or cone transducin α-subunit showing 28.3 emPAI value in the COS-rich fraction, are eliminated in Table 2. It should be mentioned that cone transducin α-subunit is a ~40 kDa protein. Proteins of this molecular mass may have been quantified only partially in our analysis on the proteins in the limited regions (Fig 5E). In other words, the emPAI value of cone transducin α-subunit could be much higher when all of them were analyzed. In the above process, ROS and COS volume differences [10] were taken into account to compare the emPAI values in the equal volume. Proteins in Table 2 are listed in descending order of COS/(COS+CIS).

Our result that proteins known to be localized outside of COS are listed in Table 2 may suggest that there is a local density difference in membranes to induce membrane contamination in our membrane separation using density gradient. In any event, the result indicates that our list (Table 2) contains COS-specific/dominant proteins but that their exact localization should be confirmed with alternative ways such as immunocytochemistry. It should be mentioned that the proteins only found in COS-rich fraction and not in CIS-rich fraction (proteins with COS/(COS+CIS) value of 1 in Table 2) show low emPAI values (Table 2), which indicates that the expression levels of these proteins are low even in COS. In addition, emPAI values of most of the proteins in Table 2 are relatively low comparing with the value of 28.3 of cone transducin α-subunit. In the list in Table 2, we excluded the COS proteins present in ROS. This result indicates that overall, protein expression profiles are similar between ROS and COS in carp in the range of the molecular mass examined in this study.

### Localization of NCALD in cones

Validation of our separation of membranes was tested by using one of the candidate proteins, neurocalcin δ B (NCALD), which was detected specifically in the COS fraction in this study. NCALD has been reported to be expressed in amacrine cells and ganglion cells [43], and its localization is also recognized in CIS [44] but not in COS. As shown in Table 2 and also in S3 and S4 Tables, we detected NCALD in COS but not in CIS, and not in ROS or RIS (S1 and S2 Tables).

**Fig 6:**
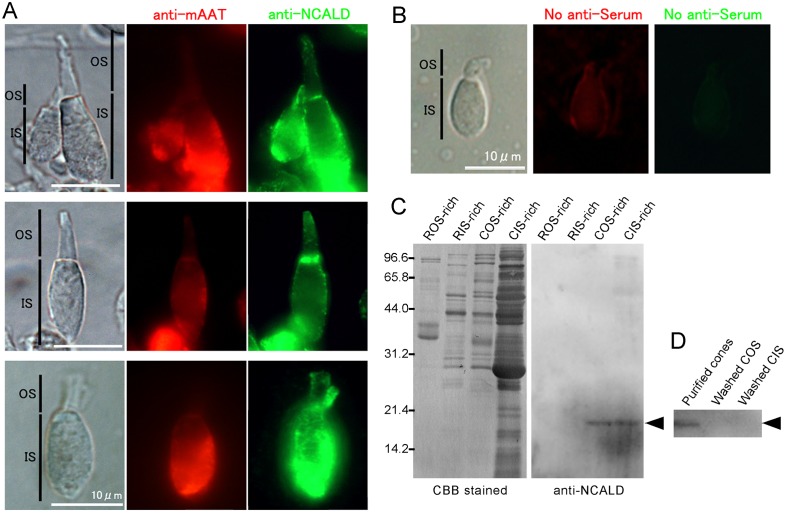
Subcellular localization of neurocalcin δ B (NCALD) in rods and cones. (A) DIC images (left panels) of double (top), single (middle) and twin (bottom) cones, and immunofluorescent images of antiserum against anti-mAAT (middle panels) and those of NCALD (right panels). Scale bars, 10 μm. OS, outer segment. IS, inner segment. (B) Negative control of (A) with use of control mouse serum (middle for mAAT) and rabbit serum (right for NCALD). A cone was only faintly labeled with these control sera. (C) Immunoblot analysis of non-washed membranes in ROS-, RIS-, COS- and CIS-rich fractions (ROS-rich, RIS-rich, COS-rich and CIS-rich, respectively). Membranes in these fractions were subjected to SDS-PAGE and were stained with CBB or probed with antibody against NCALD. NCALD signals were observed in membranes of COS-rich and CIS-rich fractions (arrowhead). The membranes of ROS- and RIS-rich fraction were obtained from 2.0 × 10^5^ rods. The membranes of COS- and CIS-rich fraction were obtained from 2.0 × 10^5^ and 5.0 × 10^4^ cones, respectively, to observe similar intensity of immunoblot signals of NCALD. (D) Immunoblot signals of NCALD (arrowhead) on membranes obtained from 2.0 × 10^5^ purified cone membranes (Purified cones), washed COS membranes obtained from 2.5 × 10^6^ purified cones (Washed COS) and washed CIS membrane obtained from 2.5 × 10^6^ purified cones (Washed CIS).

We examined localization of NCALD in isolated cones. As has been reported previously [44], NCALD was present in the IS (anti-NCALD in Fig 6A) similarly as mitochondrial aspartate aminotransferase 2 (anti-mAAT in Fig 6A), a marker protein of mitochondrial inner membranes in IS [45]. In addition, some punctate NCALD signals were detected also in the COS (Fig 6A, anti-NCALD) in the apical region of a COS as well as at the base of a COS, possibly the calycal process. The apparent discrepancy between this immunocytochemistry (mainly present in the CIS and slightly in COS) and our LC-MS/MS analysis shown in Table 2 (only present in COS membranes) could be due to the difference in the membranes probed in these studies: in immunocytochemistry, native membranes in living cells were probed while in the LC-MS/MS analysis, membranes washed intensively were used. This possibility was examined with immunoblot.

In our immunoblot analysis in non-washed membranes of ROS-rich and RIS-rich fractions (ROS-rich and RIS-rich in Fig 6C), we did not observe NCALD signals (arrowhead), while we detected positive signals in non-washed COS-rich and CIS rich fractions (COS-rich and CIS-rich in Fig 6C). The intensity of the signal was higher in the CIS-rich fraction (the number of purified cones used in Fig 6C was smaller for CIS-rich fraction, see legend to Fig 6C), which is in agreement with the immunocytochemical study shown in Fig 6A. However, in membranes intensively washed (washed membranes), which were used for LC-MS/MS analysis, we did not see any NCALD signals (Fig 6D, arrowhead) even when the membranes applied for SDS-PAGE increased by 12.5 times for washed COS membranes and 50 times for washed CIS membranes (Washed COS and Washed CIS, respectively in Fig 6D) than those used in Fig 6C.

Neurocalcin is a member of the neuronal calcium sensor family [46, 47], and is thought to be soluble under low Ca^2+^ conditions. Therefore, it is possible that most of NCALD were removed from membranes during the wash of membranes used for LC-MS/MS analysis, because Ca^2+^ concentrations in washing buffers were low. This would be the reason why NCALD was not detected in washed CIS-rich membranes. Previous studies showed that NCALD is able to interact directly with retinal guanylyl cyclase 1 (ROS-GC1) [44], which is known to be localized to the rod OS membranes. In carp cones, cone guanylate cyclase (GC-C [6] or guanylyl cyclase 3) is expressed as the homolog of bovine ROS-GC1. GC-C was detected in COS-rich membranes in our LC-MS/MS analysis (S3 Table). It is possible that a few amount of NCALD, which is bound to GC-C in washed COS-rich membranes, is enough to be detected in the LC-MS/MS analysis (Table 2 and S4 Table) but not in immunoblot analysis (Fig 6D). GC-C was actually detected in our LC-MS/MS analysis in the washed COS membranes but not in the washed CIS membranes (S3 and S4 Tables, respectively). However, GC-C was also detected in ROS-rich fraction (S1 Table) probably because of contamination of cones in purified rod preparation (see Materials and methods). It is the reason why GC-C is not listed in Table 2.

### Proteomic analysis on proteins expressed in OS and IS in rods and cones

It has been known that rods and cones are different in many aspects. For example, phototransduction in the OS is different [1–3], OS morphology is different [1–3], energy metabolism is different [48], etc. Transcriptomic analyses of purified rods and cones have been performed previously [49–51], and in these studies, proteins or genes that are specifically or dominantly expressed in either rods or cones were found. These proteins or genes are potentially the candidates participating in the rod-cone differences. However, it is not easy to identify their functional roles generally. On this point, it is very probable that those proteins are expressed at the site where the difference is observed. In this study, we established a method to obtain OS and IS membranes effectively from purified rods and cones in a quantity large enough to perform proteomic analysis. Based on our analysis, we actually succeeded in identifying a protein that localized to COS membranes but not in CIS, ROS or RIS membranes (Fig 6), although these membranes were intensively washed. Our results therefore, could contribute to the study aiming at finding proteins specifically expressed at the appropriate site, OS or IS in rods or cones, to exert their specific functions.

In this study, as a first attempt, we limited our analysis on proteins tightly bound to membranes to evaluate how our separation, rods vs cones and OS vs IS, is effective. We can potentially extend our studies to weakly membrane-associated peripheral proteins to study the molecular bases on the differences between rods and cones.

## Supporting information

S1 Table Identified proteins in washed ROS-rich fraction. Proteins in washed ROS-rich fraction were identified with LC-MS/MS analysis and are listed in descending order of emPAI values for 5 × 10^5^ rods.
(PDF)

S2 Table Identified proteins in washed RIS-rich fraction. Proteins in washed RIS-rich fraction were identified with LC-MS/MS analysis and are listed in descending order of emPAI values for 5 × 10^5^ rods.
(PDF)

S3 Table Identified proteins in washed COS-rich fraction. Proteins in washed COS-rich fraction were identified with LC-MS/MS analysis and are listed in descending order of emPAI values for 5 × 10^5^ cones.
(PDF)

S4 Table Identified proteins in washed CIS-rich fraction. Proteins in washed CIS-rich fraction were identified with LC-MS/MS analysis and are listed in descending order of emPAI values for 5 × 10^5^ cones.
(PDF)
