# Supplementary material for: Purification of cone outer segment for proteomic analysis on its membrane proteins in carp retina
Source: PLoS One. 2017 Mar 14;12(3):e0173908. doi: 10.1371/journal.pone.0173908 (PMC5349680; doi:10.1371/journal.pone.0173908)
Supplement: S1 Table — Proteins in washed ROS-rich fraction were identified with LC-MS/MS analysis and are listed in descending order of emPAI values for 5 × 10^5^ rods. (PDF) [file pone.0173908.s001.pdf]

S1 Table. Identified proteins in washed ROS-rich fraction. Proteins in washed ROS-rich fraction were identified with LC-MS/MS analysis and are listed in descending order of emPAI values for 5×10^5 rods.

	Identified proteins in washed ROS-rich fraction	Molecular mass	emPAI
1	uncharacterized protein LOC100145214	33 kDa	7.7948
2	peripherin-2	21 kDa	6.1222
3	rhodopsin	40 kDa	5.7215
4	guanine nucleotide-binding protein G(I)/G(S)/G(T) subunit beta-1	37 kDa	2.6851
5	ras-related protein Rab-2A	24 kDa	2.6161
6	peripherin-2	18 kDa	2.1304
7	rod outer segment membrane protein 1	25 kDa	1.9179
8	retinal guanylyl cyclase 2 (guanylate cyclase retinal rod2 [Cyprinus carpio])	124 kDa	1.8202
9	guanine nucleotide-binding protein G(I)/G(S)/G(T) subunit beta-1	37 kDa	1.7943
10	PREDICTED: pyruvate kinase PKM isoform X1	60 kDa	1.5077
11	rod outer segment membrane protein 1	40 kDa	1.4684
12	PREDICTED: olfactory guanylyl cyclase GC-D isoform X3 (guanylate cyclase retinal rod1 [Cyprinus carpio])	88 kDa	1.4548
13	ATP synthase F(0) complex subunit B1, mitochondrial	31 kDa	1.3896
14	PREDICTED: uncharacterized protein LOC571872 isoform X1	32 kDa	1.2876
15	retinal guanylyl cyclase 2 (guanylate cyclase retinal rod2 [Cyprinus carpio])	125 kDa	1.2573
16	voltage-dependent anion-selective channel protein 2	30 kDa	1.0218
17	peripherin-2	20 kDa	0.9849
18	PREDICTED: ras-related protein Rab-1A-like isoform X1	22 kDa	0.9094
19	ras-related protein Rab-1B	22 kDa	0.8998
20	PREDICTED: ras-related protein Rab-11B	25 kDa	0.8862
21	uncharacterized protein LOC100005305	11 kDa	0.8181
22	PREDICTED: solute carrier family 25 (mitochondrial carrier; phosphate carrier), member 3a isoform X2	40 kDa	0.7296
23	PREDICTED: sema domain, immunoglobulin domain (Ig), short basic domain, secreted, (semaphorin) 3Fa isoform X2	40 kDa	0.7255
24	PREDICTED: pleckstrin homology domain-containing family B member 1 isoform X1	21 kDa	0.6706
25	ADP-ribosylation factor-like protein 8B-A	21 kDa	0.6706
26	voltage-dependent anion-selective channel protein 1	31 kDa	0.6616
27	PREDICTED: ATP synthase subunit gamma, mitochondrial isoform X1	33 kDa	0.6449
28	PREDICTED: tubulin beta-4B chain-like	31 kDa	0.643
29	PREDICTED: ras-related protein Rab-10	23 kDa	0.6025
30	PREDICTED: uncharacterized protein LOC571872 isoform X1	23 kDa	0.5861
31	mitochondrial 2-oxoglutarate/malate carrier protein	38 kDa	0.5717
32	ras-related protein Rap-1b-like	21 kDa	0.5664
33	ATP synthase subunit alpha, mitochondrial	33 kDa	0.5559
34	PREDICTED: prohibitin isoform X2	22 kDa	0.5396
35	PREDICTED: prohibitin	22 kDa	0.5346
36	ADP/ATP translocase 3	27 kDa	0.5263
37	olfactory guanylyl cyclase GC-D (guanylate cyclase retinal rod1 [Cyprinus carpio])	34 kDa	0.5262
38	protein NDRG1 isoform 1	42 kDa	0.5211
39	putative tubulin beta chain variant 1	20 kDa	0.5103
40	prohibitin 2a	35 kDa	0.4993
41	neurotrimin isoform 1 precursor	38 kDa	0.4975
42	ras-related protein Rab-18	23 kDa	0.4926
43	ADP-ribosylation factor-like protein 8A	25 kDa	0.4906
44	PREDICTED: olfactory guanylyl cyclase GC-D isoform X2 (guanylate cyclase retinal rod1 [Cyprinus carpio])	26 kDa	0.4683
45	ras-related protein Rap-1b precursor	21 kDa	0.4628
46	PREDICTED: ADP-ribosylation factor-like protein 13B-like isoform X5	27 kDa	0.432
47	ras-related protein Rab-11A	22 kDa	0.4156
48	dnaJ homolog subfamily C member 5G	22 kDa	0.4086
49	uncharacterized protein LOC492355	23 kDa	0.3888
50	PREDICTED: voltage-dependent anion-selective channel protein 2	11 kDa	0.3817
51	ras-related protein Rab-35	20 kDa	0.379

52	PREDICTED: cGMP-gated cation channel alpha-1	63 kDa	0.374
53	RAB5A, member RAS oncogene family, a	24 kDa	0.3736
54	voltage-dependent anion-selective channel protein 2-like	30 kDa	0.3663
55	PREDICTED: protein RD3-like	24 kDa	0.3623
56	PREDICTED: ATP synthase subunit alpha, mitochondrial	27 kDa	0.3572
57	PREDICTED: cGMP-gated cation channel alpha-1	63 kDa	0.3443
58	PREDICTED: uncharacterized protein LOC571872 isoform X1	12 kDa	0.3431
59	protein disulfide-isomerase TMX3 precursor	47 kDa	0.3406
60	long-chain fatty acid transport protein 4	25 kDa	0.3365
61	ras-related protein Rab-1B	19 kDa	0.3323
62	ATP synthase subunit O, mitochondrial	26 kDa	0.3271
63	synaptosomal-associated protein 25-B	23 kDa	0.3179
64	peripherin-2	20 kDa	0.3062
65	PREDICTED: prohibitin-2	27 kDa	0.3038
66	ras-related protein Rab-5C	37 kDa	0.3011
67	RAB11a, member RAS oncogene family, like	20 kDa	0.3009
68	PREDICTED: tubulin alpha chain	45 kDa	0.2956
69	ras-related protein Rab-14	24 kDa	0.2927
70	PREDICTED: ADP-ribosylation factor-like protein 13B-like isoform X5	42 kDa	0.2853
71	ADP-ribosylation factor-like protein 6	21 kDa	0.2792
72	creatine kinase S-type, mitochondrial	47 kDa	0.2761
73	PREDICTED: creatine kinase S-type, mitochondrial isoform X2	47 kDa	0.2751
74	putative Ras-related protein Rab-42	18 kDa	0.2746
75	cytochrome c-1	36 kDa	0.2686
76	small GTPase RhoA	22 kDa	0.2642
77	synaptobrevin homolog YKT6	22 kDa	0.2544
78	ATPase, Na+/K+ transporting, beta 2b polypeptide	34 kDa	0.2521
79	PREDICTED: phospholipid scramblase 2	26 kDa	0.2508
80	PREDICTED: phosphate carrier protein, mitochondrial-like isoform X1	34 kDa	0.2484
81	PREDICTED: tubulin beta-2B chain-like isoform 1	55 kDa	0.2433
82	PREDICTED: voltage-dependent anion-selective channel protein 2	11 kDa	0.243
83	ras-related protein Rab-18-B	23 kDa	0.2418
84	uncharacterized protein LOC449549	23 kDa	0.2401
85	ATP synthase subunit g, mitochondrial	11 kDa	0.2396
86	ras-related protein Ral-A	23 kDa	0.2384
87	PREDICTED: ATPase, Na+/K+ transporting, beta 2b polypeptide isoform X1	24 kDa	0.2351
88	RAB1A, member RAS oncogene family	28 kDa	0.2331
89	PREDICTED: tubulin beta-4B chain-like, partial	16 kDa	0.2301
90	PREDICTED: LOW QUALITY PROTEIN: actin, gamma 1	40 kDa	0.2296
91	voltage-dependent anion-selective channel protein 2	20 kDa	0.2266
92	ubiquitin-60S ribosomal protein L40	12 kDa	0.2209
93	NAD(P) transhydrogenase, mitochondrial	114 kDa	0.2185
94	PREDICTED: ras-related protein Rab-28 isoform X2	25 kDa	0.2185
95	immunity-related GTPase family, q2	42 kDa	0.2181
96	PREDICTED: vesicle-associated membrane protein 2-like	12 kDa	0.2153
97	catechol-O-methyltransferase a	30 kDa	0.2119
98	ER membrane protein complex subunit 3	30 kDa	0.2107
99	peripherin 2b (retinal degeneration, slow)	39 kDa	0.2078
100	60S ribosomal protein L18	22 kDa	0.2022
101	PREDICTED: tubulin alpha-1C chain	45 kDa	0.1988
102	guanylyl cyclase 3 (guanylate cyclase retinal cone [Cyprinus carpio])	123 kDa	0.1967
103	PREDICTED: GTP-binding protein SAR1b-like	13 kDa	0.193
104	PREDICTED: regulator of G-protein signaling 9-binding protein B	28 kDa	0.1869
105	guanylyl cyclase 3 (guanylate cyclase retinal cone [Cyprinus carpio])	128 kDa	0.1853
106	PREDICTED: alpha/beta hydrolase domain-containing protein 17A	28 kDa	0.1808
107	PREDICTED: ras-related protein Rab-7a isoform X1	24 kDa	0.18
108	PREDICTED: protein RD3-like	24 kDa	0.1765
109	epidermal retinol dehydrogenase 2	34 kDa	0.1765
110	PREDICTED: cadherin-related family member 5-like isoform X2	44 kDa	0.1752

111	vesicle-trafficking protein SEC22b-A	24 kDa	0.1722
112	PREDICTED: L-lactate dehydrogenase B-B chain isoform X3	19 kDa	0.1705
113	PREDICTED: NADH dehydrogenase	20 kDa	0.1692
114	PREDICTED: tubulin alpha chain-like	50 kDa	0.1692
115	retinol dehydrogenase 8a	35 kDa	0.1652
116	mitochondrial inner membrane protein	83 kDa	0.1614
117	PREDICTED: josephin-2 isoform X1	21 kDa	0.1549
118	PREDICTED: ADP-ribosylation factor 2	16 kDa	0.1509
119	uncharacterized protein LOC393228	21 kDa	0.1507
120	S-arrestin	44 kDa	0.1503
121	PREDICTED: uncharacterized protein si:dkey-182g1.3	16 kDa	0.1496
122	rhodopsin kinase	27 kDa	0.1481
123	PREDICTED: transmembrane emp24 domain-containing protein 7 isoform X2	27 kDa	0.1465
124	reticulon-4	22 kDa	0.1447
125	PREDICTED: peripherin-2	39 kDa	0.1447
126	NADH-cytochrome b5 reductase 1	28 kDa	0.1442
127	reticulon-4	22 kDa	0.1438
128	ras-related protein Rab-3A	28 kDa	0.1405
129	erlin-2 precursor	40 kDa	0.14
130	uncharacterized protein LOC100127828	34 kDa	0.1396
131	guanine nucleotide-binding protein G(t) subunit alpha-2	40 kDa	0.1395
132	PREDICTED: mitochondrial glutamate carrier 1	34 kDa	0.139
133	PREDICTED: red-sensitive opsin-1 isoform X1	41 kDa	0.138
134	PREDICTED: cadherin-related family member 5-like isoform X2	17 kDa	0.1338
135	vesicle-associated membrane protein-associated protein A	30 kDa	0.133
136	PREDICTED: ras-related protein Rab-6A isoform X2	24 kDa	0.1325
137	ras-related protein Rab-5B	24 kDa	0.1317
138	60S ribosomal protein L24	18 kDa	0.1295
139	CDP-diacylglycerol--inositol 3-phosphatidyltransferase	24 kDa	0.1294
140	40S ribosomal protein S5	25 kDa	0.1242
141	PREDICTED: saccharopine dehydrogenase-like oxidoreductase-like	12 kDa	0.124
142	peroxisomal membrane protein 11B	25 kDa	0.1228
143	PREDICTED: LOW QUALITY PROTEIN: 40S ribosomal protein S13-like	19 kDa	0.1227
144	PREDICTED: ADP-dependent glucokinase isoform X2	58 kDa	0.1199
145	PREDICTED: ER membrane protein complex subunit 1 isoform X1	111 kDa	0.1193
146	sorting and assembly machinery component 50 homolog A	52 kDa	0.1182
147	sodium/potassium-transporting ATPase subunit alpha-3	113 kDa	0.1173
148	ADP-ribosylation factor-like protein 9	26 kDa	0.1162
149	PREDICTED: vesicle transport through interaction with t-SNAREs homolog 1A isoform X1	26 kDa	0.1162
150	cytochrome c oxidase subunit 4 isoform 1, mitochondrial	20 kDa	0.1149
151	PREDICTED: phospholipid-transporting ATPase IB isoform X6	134 kDa	0.1089
152	PREDICTED: regulator of G-protein signaling 9 isoform X1	57 kDa	0.1066
153	PREDICTED: ER membrane protein complex subunit 1 isoform X1	114 kDa	0.1065
154	rhodopsin kinase	64 kDa	0.1061
155	histone 2, H2a	14 kDa	0.1048
156	PREDICTED: 40S ribosomal protein S3-like isoform X1	28 kDa	0.1045
157	PREDICTED: cGMP-gated cation channel alpha-1	36 kDa	0.1044
158	transmembrane protein 33	29 kDa	0.104
159	guanine nucleotide-binding protein subunit beta-5	43 kDa	0.1034
160	very-long-chain enoyl-CoA reductase	36 kDa	0.1028
161	retinol dehydrogenase-like	36 kDa	0.1024
162	lysophosphatidylcholine acyltransferase 2	60 kDa	0.1002
163	creatine kinase U-type, mitochondrial	22 kDa	0.1001
164	60S ribosomal protein L9	22 kDa	0.1001
165	rho-related gtp-binding protein rhoc	22 kDa	0.0995
166	retinol dehydrogenase 13	37 kDa	0.0994
167	PREDICTED: mitochondrial pyruvate carrier 2	14 kDa	0.0991
168	PREDICTED: cadherin-related family member 1-like isoform X2	99 kDa	0.0988
169	ras homolog gene family, member A	22 kDa	0.0983

170	PREDICTED: ras-related protein Rab-30	15 kDa	0.0982
171	PREDICTED: 60S ribosomal protein L6	30 kDa	0.0979
172	PREDICTED: GTP-binding protein SAR1b	22 kDa	0.0977
173	PREDICTED: 40S ribosomal protein S9-like	22 kDa	0.0971
174	hexokinase-1	54 kDa	0.0961
175	ADP-ribosylation factor-like 15a	23 kDa	0.0954
176	ras-related protein Rab-8A	23 kDa	0.0954
177	NADH dehydrogenase 1 beta subcomplex subunit 6	15 kDa	0.0947
178	40S ribosomal protein S25	15 kDa	0.0931
179	PREDICTED: CSC1-like protein 2 isoform X2	87 kDa	0.093
180	hexokinase-1	71 kDa	0.0929
181	surfeit gene 4, like	23 kDa	0.0927
182	Beta-centractin	15 kDa	0.0923
183	PREDICTED: sodium/potassium/calcium exchanger 1 isoform X1	80 kDa	0.0922
184	solute carrier family 3 (amino acid transporter heavy chain), member 2b	57 kDa	0.0897
185	NADH dehydrogenase	58 kDa	0.0887
186	PREDICTED: NADH dehydrogenase (ubiquinone) complex I, assembly factor 6	33 kDa	0.0875
187	isocitrate dehydrogenase	50 kDa	0.0861
188	PREDICTED: threonine dehydratase, mitochondrial-like isoform X2	60 kDa	0.0843
189	LETM1 and EF-hand domain-containing protein 1, mitochondrial	86 kDa	0.0843
190	dihydrolipoyllysine-residue acetyltransferase component of pyruvate dehydrogenase complex, mitochondrial	69 kDa	0.0835
191	sideroflexin-4	35 kDa	0.0825
192	acyl-CoA synthetase long-chain family member 3b	79 kDa	0.0822
193	DDRGK domain-containing protein 1 precursor	35 kDa	0.0822
194	malate dehydrogenase, mitochondrial	35 kDa	0.0801
195	PREDICTED: choline transporter-like protein 1	72 kDa	0.08
196	PREDICTED: oxoglutarate (alpha-ketoglutarate) dehydrogenase (lipoamide) isoform X3	26 kDa	0.0798
197	PREDICTED: protein-L-isoaspartate(D-aspartate) O-methyltransferase isoform X1	26 kDa	0.0794
198	PREDICTED: 40S ribosomal protein S24	17 kDa	0.0794
199	PREDICTED: regulator of G-protein signaling 9-binding protein-like	27 kDa	0.079
200	ragulator complex protein LAMTOR1	18 kDa	0.0788
201	60S ribosomal protein L14	18 kDa	0.0782
202	epimerase family protein SDR39U1	36 kDa	0.078
203	alpha-1,3/1,6-mannosyltransferase ALG2	46 kDa	0.0779
204	PREDICTED: actin, aortic smooth muscle	18 kDa	0.0777
205	PREDICTED: arylacetamide deacetylase isoform X1	47 kDa	0.0755
206	PREDICTED: CSC1-like protein 2	47 kDa	0.0753
207	dolichol-phosphate mannosyltransferase subunit 1	28 kDa	0.0747
208	PREDICTED: uncharacterized protein si:rp71-36a1.2	28 kDa	0.0743
209	uncharacterized protein LOC100001558	19 kDa	0.0739
210	PREDICTED: sphingomyelin phosphodiesterase 2 isoform X1	48 kDa	0.073
211	PREDICTED: tetratricopeptide repeat protein 8-like isoform X1	58 kDa	0.0728
212	green-sensitive opsin-4	39 kDa	0.0725
213	PREDICTED: protein tyrosine phosphatase type IVA 2-like isoform X3	19 kDa	0.071
214	rod cGMP-specific 3',5'-cyclic phosphodiesterase subunit alpha	99 kDa	0.0708
215	PREDICTED: E3 ubiquitin-protein ligase RNF170-like	29 kDa	0.0704
216	plasminogen receptor (KT)	20 kDa	0.0692
217	PREDICTED: syntaxin-12 isoform X1	30 kDa	0.0686
218	PREDICTED: solute carrier family 41 member 1	40 kDa	0.0686
219	PREDICTED: potassium voltage-gated channel subfamily B member 2	93 kDa	0.0676
220	signal peptidase complex subunit 3	20 kDa	0.0674
221	mitochondrial carrier homolog 2	31 kDa	0.0669
222	ADP-ribosylation factor-like protein 1	20 kDa	0.0666
223	nicastrin precursor	42 kDa	0.0664
224	methyltransferase like 7A precursor	31 kDa	0.0663
225	palmitoyltransferase ZDHHC2	42 kDa	0.0662
226	PREDICTED: cadherin-related family member 5-like isoform X2	20 kDa	0.0662
227	40S ribosomal protein S10	20 kDa	0.0662

228	ADP-ribosylation factor-like protein 3	20 kDa	0.0662
229	signal recognition particle receptor subunit beta	31 kDa	0.0661
230	ADP-ribosylation factor 1	21 kDa	0.0654
231	PREDICTED: ras-related protein Rap-2c	21 kDa	0.0654
232	oxoglutarate (alpha-ketoglutarate) dehydrogenase (lipoamide)	21 kDa	0.0646
233	PREDICTED: casein kinase I isoform gamma-1-like isoform X2	53 kDa	0.0646
234	alpha/beta hydrolase domain-containing protein 17C	32 kDa	0.0642
235	N-acetyltransferase 14	32 kDa	0.064
236	PREDICTED: FAS-associated factor 2-like isoform X1	54 kDa	0.0636
237	PREDICTED: NADH dehydrogenase	21 kDa	0.0634
238	cell division control protein 42 homolog	21 kDa	0.0634
239	calmegin precursor	66 kDa	0.0628
240	synaptophysin b isoform 1	33 kDa	0.0625
241	40S ribosomal protein S3a	33 kDa	0.0625
242	PREDICTED: mitochondrial ubiquitin ligase activator of nfkb 1-A	33 kDa	0.061
243	PREDICTED: F-box/LRR-repeat protein 20	45 kDa	0.0606
244	PREDICTED: V-type proton ATPase subunit S1	34 kDa	0.0588
245	PREDICTED: transmembrane emp24 domain-containing protein 2	23 kDa	0.0586
246	ras-related protein Rab-24	23 kDa	0.0582
247	phosphatidylglycerophosphatase and protein-tyrosine phosphatase 1	11 kDa	0.0578
248	receptor expression-enhancing protein 6	23 kDa	0.0573
249	PREDICTED: uncharacterized protein LOC101884052	11 kDa	0.0572
250	PREDICTED: ADP-ribosylation factor-like protein 3	23 kDa	0.057
251	ras-related C3 botulinum toxin substrate 2	23 kDa	0.057
252	mitochondrial chaperone BCS1	48 kDa	0.0567
253	sideroflexin-3	36 kDa	0.0567
254	HIG1 domain family member 1A	11 kDa	0.0566
255	thioredoxin-related transmembrane protein 2-B precursor	36 kDa	0.0563
256	PREDICTED: gamma-glutamyltransferase 5	60 kDa	0.056
257	PREDICTED: aspartyl/asparaginyl beta-hydroxylase isoform X6	11 kDa	0.056
258	PREDICTED: voltage-dependent anion-selective channel protein 3 isoform X3	36 kDa	0.0557
259	PREDICTED: voltage-dependent anion-selective channel protein 3 isoform X3	36 kDa	0.0557
260	PREDICTED: 40S ribosomal protein S8	24 kDa	0.055
261	ELMO domain-containing protein 2 precursor	24 kDa	0.0544
262	PREDICTED: NADH dehydrogenase	12 kDa	0.0543
263	PREDICTED: reticulon-3-B-like isoform X2	25 kDa	0.0536
264	selenoprotein T1a precursor	12 kDa	0.0527
265	PREDICTED: cyclic nucleotide-gated cation channel beta-1 isoform X2	116 kDa	0.0524
266	histone 1, H4, like	12 kDa	0.0522
267	PREDICTED: uncharacterized protein LOC100707031 isoform X1	12 kDa	0.0517
268	PREDICTED: 40S ribosomal protein S2	12 kDa	0.0512
269	Bardet-Biedl syndrome 5 protein homolog	39 kDa	0.0511
270	PREDICTED: ADP-dependent glucokinase isoform X2	26 kDa	0.0503
271	mitochondrial ATP synthase subunit f	13 kDa	0.0502
272	mitochondrial pyruvate carrier 1	13 kDa	0.0502
273	PREDICTED: 26S proteasome non-ATPase regulatory subunit 1-like	148 kDa	0.0501
274	PREDICTED: vesicle-associated membrane protein-associated protein A-like	26 kDa	0.0501
275	PREDICTED: vesicle-associated membrane protein-associated protein A-like	26 kDa	0.0501
276	guanine nucleotide-binding protein G(o) subunit alpha	40 kDa	0.0497
277	PREDICTED: calnexin isoform X1	67 kDa	0.0494
278	PREDICTED: sodium/potassium-transporting ATPase subunit alpha-3-like	27 kDa	0.0494
279	uncharacterized protein LOC100124614	13 kDa	0.0493
280	PREDICTED: prominin-1 isoform X1	95 kDa	0.0491
281	PREDICTED: l-isoaspartyl protein carboxyl methyltransferase, like isoform X2	27 kDa	0.0489
282	phosducin	27 kDa	0.0485
283	PREDICTED: ceroid-lipofuscinosis, neuronal 6a isoform X1	19 kDa	0.0484
284	PREDICTED: transmembrane emp24 domain-containing protein 9 isoform X1	27 kDa	0.0482
285	mannose-P-dolichol utilization defect 1 protein	27 kDa	0.0478
286	very-long-chain (3R)-3-hydroxyacyl-CoA dehydratase 2	28 kDa	0.047

287	PREDICTED: protein XRP2 isoform X1	42 kDa	0.0469
288	PREDICTED: UDP-N-acetylglucosamine transporter isoform X1	14 kDa	0.0467
289	ATP synthase F(0) complex subunit C3, mitochondrial	14 kDa	0.0463
290	fatty aldehyde dehydrogenase	28 kDa	0.0462
291	3-hydroxyacyl-CoA dehydrogenase type-2	28 kDa	0.046
292	PREDICTED: hexokinase-1-like	14 kDa	0.0459
293	PREDICTED: solute carrier family 1 (glial high affinity glutamate transporter), member 2a isoform X2	58 kDa	0.0456
294	PREDICTED: coiled-coil-helix-coiled-coil-helix domain-containing protein 6, mitochondrial isoform X2	29 kDa	0.0456
295	aquaporin-9	29 kDa	0.0456
296	PREDICTED: alkaline phosphatase, tissue-nonspecific isozyme isoform X2	58 kDa	0.0455
297	PREDICTED: retinal-specific ATP-binding cassette transporter isoform X1	265 kDa	0.0453
298	histone 2, H2a	14 kDa	0.0451
299	PREDICTED: uncharacterized protein LOC565091 isoform X1	14 kDa	0.0451
300	40S ribosomal protein S4, X isoform	29 kDa	0.0446
301	basigin precursor	45 kDa	0.044
302	alpha/beta hydrolase domain-containing protein 14A	30 kDa	0.0435
303	calcium-binding mitochondrial carrier protein Aralar1	76 kDa	0.0434
304	protein RD3	15 kDa	0.0433
305	PREDICTED: surfeit locus protein 4	30 kDa	0.0432
306	excitatory amino acid transporter 2	61 kDa	0.0429
307	F-box/LRR-repeat protein 2	46 kDa	0.0427
308	NADH dehydrogenase 1 beta subcomplex subunit 4	15 kDa	0.0426
309	membrane magnesium transporter 1 precursor	15 kDa	0.0426
310	prominin-1 precursor	94 kDa	0.0422
311	ES1 protein, mitochondrial precursor	31 kDa	0.042
312	PREDICTED: SEC14-like protein 3 isoform X1	31 kDa	0.0417
313	PREDICTED: GTPase HRas-like	15 kDa	0.0416
314	PREDICTED: dehydrogenase/reductase SDR family member 7B isoform X1	31 kDa	0.0415
315	PREDICTED: progestin and adipoQ receptor family member 4-like	31 kDa	0.0415
316	flotillin-2a	47 kDa	0.0413
317	uncharacterized protein LOC562845	31 kDa	0.041
318	mitochondrial dicarboxylate carrier	32 kDa	0.0409
319	PREDICTED: lipid phosphate phosphohydrolase 1 isoform X1	32 kDa	0.0407
320	serum/glucocorticoid regulated kinase 1-like	48 kDa	0.0403
321	NADH dehydrogenase 1 alpha subcomplex subunit 6	16 kDa	0.04
322	PREDICTED: rod cGMP-specific 3',5'-cyclic phosphodiesterase subunit beta	99 kDa	0.0397
323	PREDICTED: sarcoplasmic/endoplasmic reticulum calcium ATPase 2	116 kDa	0.0394
324	ATPase, Na+/K+ transporting, beta 2a polypeptide	16 kDa	0.0392
325	PREDICTED: aspartate beta-hydroxylase isoform X2	33 kDa	0.0391
326	protein NipSnap homolog 2	33 kDa	0.0388
327	trifunctional enzyme subunit beta, mitochondrial	50 kDa	0.0387
328	creatine kinase b-type	16 kDa	0.0386
329	PREDICTED: lipid phosphate phosphohydrolase 1-like	33 kDa	0.0385
330	mitochondrial import receptor subunit TOM20 homolog B	16 kDa	0.0383
331	PREDICTED: casein kinase I isoform gamma-2 isoform X1	51 kDa	0.0381
332	ubiquitin-conjugating enzyme E2Nb	16 kDa	0.038
333	uncharacterized protein LOC492776	16 kDa	0.038
334	PREDICTED: hydroxyacylglutathione hydrolase, mitochondrial isoform X1	34 kDa	0.0378
335	protein-S-isoprenylcysteine O-methyltransferase	34 kDa	0.0378
336	PREDICTED: uncharacterized protein LOC100694464	17 kDa	0.0375
337	60S ribosomal protein L22	17 kDa	0.0375
338	mitochondrial import inner membrane translocase subunit TIM44	52 kDa	0.0373
339	protein disulfide-isomerase TMX3 precursor	52 kDa	0.0372
340	PREDICTED: retinol dehydrogenase 12-like	34 kDa	0.0371
341	PREDICTED: NADH dehydrogenase	17 kDa	0.037
342	inactive hydroxysteroid dehydrogenase-like protein 1	35 kDa	0.0368
343	dehydrogenase/reductase (SDR family) member 13a, duplicate 3	35 kDa	0.0368
344	uncharacterized protein LOC100135257	35 kDa	0.0365

345	elongation of very long chain fatty acids-like 4	35 kDa	0.0365
346	PREDICTED: uncharacterized protein LOC100126019 isoform X1	107 kDa	0.0362
347	protein YIF1A	35 kDa	0.036
348	PREDICTED: 40S ribosomal protein S23-like	17 kDa	0.036
349	PREDICTED: creatine kinase S-type, mitochondrial isoform X2	17 kDa	0.0357
350	PREDICTED: 60S ribosomal protein L23	17 kDa	0.0357
351	peptidyl-prolyl cis-trans isomerase A	18 kDa	0.0355
352	PREDICTED: 60S ribosomal protein L12	18 kDa	0.0353
353	alpha-internexin	55 kDa	0.0352
354	optic atrophy 3 protein homolog	18 kDa	0.035
355	60S ribosomal protein L26	18 kDa	0.035
356	protein lunapark-A	36 kDa	0.035
357	PREDICTED: PI-PLC X domain-containing protein 2-like	37 kDa	0.0348
358	60S ribosomal protein L27a	18 kDa	0.0348
359	PREDICTED: mitochondrial fission process protein 1-like	18 kDa	0.0344
360	coiled-coil domain-containing protein 47 precursor	56 kDa	0.0343
361	ATP synthase subunit d, mitochondrial	18 kDa	0.0341
362	uncharacterized protein LOC100127838	18 kDa	0.0341
363	PREDICTED: 60S ribosomal protein L23a	18 kDa	0.0341
364	PREDICTED: 60S ribosomal protein L27-like	18 kDa	0.0341
365	40S ribosomal protein S18	18 kDa	0.0339
366	PREDICTED: histidine triad nucleotide-binding protein 3 isoform X1	18 kDa	0.0339
367	40S ribosomal protein S16	18 kDa	0.0337
368	coiled-coil domain-containing protein 126	18 kDa	0.0337
369	uncharacterized protein LOC100005305	19 kDa	0.0335
370	neutral amino acid transporter B(0)	57 kDa	0.0334
371	coiled-coil domain-containing protein 126	19 kDa	0.0333
372	golgin A7 family, member Ba	19 kDa	0.0333
373	PREDICTED: atlastin-3 isoform X1	58 kDa	0.033
374	glutathione S-transferase theta-like	19 kDa	0.0329
375	stomatin-like protein 2, mitochondrial	39 kDa	0.0322
376	pyruvate dehydrogenase E1 component subunit beta, mitochondrial	39 kDa	0.0322
377	PREDICTED: protein ATP1B4	39 kDa	0.0322
378	fatty-acid amide hydrolase 2-A	59 kDa	0.0322
379	D-beta-hydroxybutyrate dehydrogenase, mitochondrial	40 kDa	0.0317
380	uncharacterized protein LOC570464 precursor	20 kDa	0.0317
381	PREDICTED: potassium voltage-gated channel subfamily V member 2	60 kDa	0.0316
382	keratin, type II cytoskeletal 8	61 kDa	0.0315
383	golgin A7 family, member Ba	20 kDa	0.0315
384	eukaryotic translation initiation factor 2 subunit 2	20 kDa	0.0315
385	apoptosis-inducing factor 2	41 kDa	0.0312
386	PREDICTED: peroxiredoxin-5, mitochondrial-like	20 kDa	0.0308
387	PREDICTED: 40S ribosomal protein S2	20 kDa	0.0308
388	PREDICTED: ADP-ribosylation factor 6-like	20 kDa	0.0308
389	PREDICTED: protein tyrosine phosphatase type IVA 3-like isoform X3	20 kDa	0.0308
390	PREDICTED: 11-cis retinol dehydrogenase isoform X1	41 kDa	0.0304
391	transmembrane protein 30Aa	42 kDa	0.0303
392	PREDICTED: photoreceptor outer segment membrane glycoprotein 2-like isoform X1	42 kDa	0.0303
393	LIM domain and actin-binding protein 1	20 kDa	0.0301
394	PREDICTED: signal peptidase complex catalytic subunit SEC11A-like	21 kDa	0.0301
395	ADP-ribosylation factor-like protein 5A	21 kDa	0.0301
396	PREDICTED: guanine nucleotide-binding protein subunit alpha-11-like isoform X1	42 kDa	0.0299
397	PREDICTED: exportin-1-like, partial	21 kDa	0.0299
398	guanine nucleotide-binding protein G(I)/G(S)/G(T) subunit beta-3	21 kDa	0.0294
399	brain creatine kinase b	43 kDa	0.0294
400	PREDICTED: translocon-associated protein subunit gamma	21 kDa	0.0293
401	PREDICTED: cell division control protein 42 homolog isoform X1	21 kDa	0.0289
402	aspartate aminotransferase 2	43 kDa	0.0289
403	PREDICTED: glutaminase a isoform X1	66 kDa	0.0289

404	PREDICTED: transmembrane and coiled-coil domains protein 1-like	21 kDa	0.0288
405	peripherin 2, like	21 kDa	0.0288
406	outer dense fiber of sperm tails 2b	22 kDa	0.0286
407	ADP-ribosylation-like factor 6 interacting protein 5	22 kDa	0.0286
408	PREDICTED: ras-related C3 botulinum toxin substrate 1	22 kDa	0.0286
409	AFG3-like protein 2	89 kDa	0.0286
410	PREDICTED: guanine nucleotide-binding protein G(s) subunit alpha isoform X2	44 kDa	0.0283
411	phosphatidylinositide phosphatase SAC1-B	67 kDa	0.0281
412	protein kinase, cAMP-dependent, regulatory, type II, alpha A	45 kDa	0.028
413	ras-related protein R-Ras	22 kDa	0.0279
414	PREDICTED: glucose-induced degradation protein 8 homolog	22 kDa	0.0277
415	PREDICTED: charged multivesicular body protein 6-like isoform X1	22 kDa	0.0276
416	PREDICTED: transmembrane protein 126A isoform X1	22 kDa	0.0274
417	protein phosphatase 2, regulatory subunit B', epsilon isoform a	22 kDa	0.0274
418	synaptotagmin II	47 kDa	0.0269
419	PREDICTED: cadherin-related family member 5-like isoform X2	23 kDa	0.0269
420	adipocyte plasma membrane-associated protein	47 kDa	0.0268
421	membrane-associated progesterone receptor component 2	24 kDa	0.0261
422	PREDICTED: metal transporter CNNM4	24 kDa	0.0261
423	PREDICTED: NADPH--cytochrome P450 reductase isoform X1	48 kDa	0.026
424	glutamine synthetase 1	24 kDa	0.0257
425	60S ribosomal protein L15	24 kDa	0.0256
426	protein THEM6 precursor	24 kDa	0.0256
427	HD domain-containing protein 2	24 kDa	0.0256
428	PREDICTED: neural cell adhesion molecule 1 isoform X1	74 kDa	0.0255
429	protein THEM6 precursor	24 kDa	0.0255
430	NADH dehydrogenase	24 kDa	0.0253
431	PREDICTED: dephospho-CoA kinase domain-containing protein-like isoform X2	24 kDa	0.0253
432	transmembrane emp24 domain-containing protein 10 precursor	24 kDa	0.0252
433	rho GTPase-activating protein 1	50 kDa	0.0251
434	PREDICTED: serine/threonine-protein kinase NIM1-like	50 kDa	0.025
435	60S ribosomal protein L10	25 kDa	0.025
436	motile sperm domain-containing protein 1	25 kDa	0.025
437	PREDICTED: testis-expressed sequence 264 protein-like	25 kDa	0.025
438	cytochrome b-c1 complex subunit 2, mitochondrial	50 kDa	0.0249
439	rab GDP dissociation inhibitor beta	51 kDa	0.0247
440	60S ribosomal protein L10a	25 kDa	0.0246
441	tubulin gamma-1 chain	51 kDa	0.0244
442	phosphatidylinositide phosphatase SAC1-A	51 kDa	0.0243
443	dolichyl-diphosphooligosaccharide--protein glycosyltransferase 48 kDa subunit precursor	51 kDa	0.0242
444	UBX domain-containing protein 4	52 kDa	0.0241
445	transmembrane protein 147	25 kDa	0.0241
446	60S ribosomal protein L17	25 kDa	0.0241
447	PREDICTED: ras-related protein Rab-9B	25 kDa	0.0241
448	PREDICTED: phosphatidate cytidylyltransferase 1	52 kDa	0.0239
449	Probable saccharopine dehydrogenase	26 kDa	0.0237
450	solute carrier family 2, facilitated glucose transporter member 1	53 kDa	0.0236
451	squalene synthase	53 kDa	0.0236
452	oxoglutarate (alpha-ketoglutarate) dehydrogenase (lipoamide)	26 kDa	0.0234
453	PREDICTED: coiled-coil domain-containing protein 136-like isoform X1	53 kDa	0.0233
454	CAAX prenyl protease 1 homolog	53 kDa	0.0233
455	BRI3-binding protein precursor	26 kDa	0.0231
456	peroxisomal biogenesis factor 3	26 kDa	0.0231
457	uncharacterized protein LOC619200	27 kDa	0.0229
458	ATP synthase subunit s, mitochondrial	27 kDa	0.0228
459	PREDICTED: probable ATP-dependent RNA helicase ddx6	54 kDa	0.0228
460	PREDICTED: fatty aldehyde dehydrogenase-like	55 kDa	0.0226
461	PREDICTED: sterol 26-hydroxylase, mitochondrial	27 kDa	0.0225
462	PREDICTED: ribosomal protein S6 kinase alpha-3 isoform X1	83 kDa	0.0224

463	sorting and assembly machinery component 50 homolog B	27 kDa	0.0224
464	PREDICTED: ATP synthase subunit beta, mitochondrial-like	56 kDa	0.0221
465	PREDICTED: sodium-coupled neutral amino acid transporter 3-like isoform X1	56 kDa	0.0219
466	ER membrane protein complex subunit 10 isoform 1 precursor	28 kDa	0.0217
467	PREDICTED: ankyrin repeat domain-containing protein 33B	57 kDa	0.0216
468	thioredoxin-dependent peroxide reductase, mitochondrial	28 kDa	0.0215
469	V-type proton ATPase subunit D	28 kDa	0.0215
470	60S ribosomal protein L7	29 kDa	0.0214
471	T-complex protein 1 subunit beta	58 kDa	0.0213
472	PREDICTED: importin-5 isoform X1	119 kDa	0.0209
473	prenylcysteine oxidase 1 precursor	59 kDa	0.0208
474	neuronal membrane glycoprotein M6-b	30 kDa	0.0206
475	catechol O-methyltransferase	30 kDa	0.0205
476	PREDICTED: protein phosphatase 1 regulatory subunit 16A	61 kDa	0.0204
477	PREDICTED: ATPase, Ca++ transporting, cardiac muscle, slow twitch 2b isoform X1	92 kDa	0.0203
478	60S ribosomal protein L7a	30 kDa	0.0203
479	PREDICTED: calcium signal-modulating cyclophilin ligand isoform X1	30 kDa	0.0203
480	microtubule-associated protein RP/EB family member 3	30 kDa	0.0201
481	aquaporin 1	30 kDa	0.02
482	PREDICTED: tetraspanin-5-like isoform X1	31 kDa	0.0199
483	E3 ubiquitin-protein ligase MARCH5	31 kDa	0.0193
484	acyl-CoA-binding domain-containing protein 5-B	32 kDa	0.0192
485	surfeit locus protein 1	32 kDa	0.0191
486	B-cell receptor-associated protein 31	32 kDa	0.0191
487	thioredoxin-related transmembrane protein 1 precursor	32 kDa	0.019
488	uncharacterized protein C18orf19 homolog B	32 kDa	0.0189
489	PREDICTED: rap1 GTPase-GDP dissociation stimulator 1-like isoform X1	32 kDa	0.0188
490	PREDICTED: protein FAM57A-like	32 kDa	0.0187
491	PREDICTED: cyclic nucleotide-gated cation channel beta-3 isoform X4	66 kDa	0.0186
492	trimeric intracellular cation channel type B	33 kDa	0.0185
493	PREDICTED: serine protease 33-like	33 kDa	0.0185
494	phosphatidylinositol N-acetylglucosaminyltransferase subunit Q	67 kDa	0.0184
495	PREDICTED: E3 ubiquitin-protein ligase CHIP isoform X2	33 kDa	0.0184
496	N-ethylmaleimide-sensitive factor attachment protein, beta	33 kDa	0.0183
497	PREDICTED: uncharacterized protein LOC323326 isoform X1	33 kDa	0.0183
498	PREDICTED: synembryn-A-like isoform X2	67 kDa	0.0183
499	RPE-retinal G protein-coupled receptor	33 kDa	0.0182
500	pyrroline-5-carboxylate reductase 1a	34 kDa	0.018
501	PREDICTED: protein CLN8 isoform X1	34 kDa	0.018
502	mitochondrial carnitine/acylcarnitine carrier protein CACL	34 kDa	0.018
503	PREDICTED: protein SCO1 homolog, mitochondrial	34 kDa	0.0179
504	60S acidic ribosomal protein P0	34 kDa	0.0177
505	solute carrier family 7, member 3	70 kDa	0.0176
506	mitochondrial Rho GTPase 2	70 kDa	0.0176
507	retinal G protein coupled receptor b	35 kDa	0.0176
508	cyclin-dependent kinase 5 activator 2	35 kDa	0.0174
509	peroxisomal membrane protein PMP34	35 kDa	0.0174
510	PREDICTED: CAAX prenyl protease 2	35 kDa	0.0172
511	PREDICTED: heme oxygenase 2	36 kDa	0.017
512	glycerophosphodiester phosphodiesterase domain-containing protein 1	36 kDa	0.0167
513	protein SCO2 homolog, mitochondrial	36 kDa	0.0167
514	PREDICTED: meckelin isoform X2	111 kDa	0.0166
515	PREDICTED: transmembrane protein 43-like	37 kDa	0.0165
516	PREDICTED: xyloside xylosyltransferase 1 isoform X1	37 kDa	0.0165
517	limbic system-associated membrane protein precursor	37 kDa	0.0164
518	solute carrier family 35 member B1	37 kDa	0.0164
519	casein kinase 1, alpha 1	38 kDa	0.0161
520	inositol monophosphatase 3	37 kDa	0.0161
521	PREDICTED: epoxide hydrolase 1, partial	38 kDa	0.0161

522	PREDICTED: uncharacterized protein si:ch211-132g1.3 isoform X6	38 kDa	0.016
523	PREDICTED: tumor suppressor candidate 3	38 kDa	0.0159
524	metal transporter CNNM2	38 kDa	0.0159
525	abhydrolase domain-containing protein 16A	38 kDa	0.0158
526	PREDICTED: monoacylglycerol lipase abhd6-A	38 kDa	0.0158
527	dynamin-1-like protein	77 kDa	0.0158
528	PREDICTED: cadherin-related family member 2	39 kDa	0.0157
529	PREDICTED: ATP-dependent 6-phosphofructokinase, liver type-like	39 kDa	0.0155
530	erlin-1 precursor	39 kDa	0.0155
531	eukaryotic translation initiation factor 4A, isoform 1A	39 kDa	0.0155
532	PREDICTED: ATP-dependent RNA helicase DDX3X isoform X6	79 kDa	0.0154
533	PREDICTED: ATP-dependent zinc metalloprotease YME1L1 isoform X1	79 kDa	0.0154
534	opsin-1, short-wave-sensitive 2	39 kDa	0.0154
535	opsin-1, short-wave-sensitive 1	39 kDa	0.0154
536	NADH-ubiquinone oxidoreductase 75 kDa subunit, mitochondrial	80 kDa	0.0153
537	PREDICTED: cyclic nucleotide-gated channel rod photoreceptor subunit alpha-like	82 kDa	0.015
538	PREDICTED: electrogenic sodium bicarbonate cotransporter 1 isoform X2	123 kDa	0.0149
539	cAMP-dependent protein kinase catalytic subunit beta	41 kDa	0.0149
540	PREDICTED: presenilins-associated rhomboid-like protein, mitochondrial	41 kDa	0.0149
541	flotillin-1	41 kDa	0.0149
542	PREDICTED: ribosomal protein S6 kinase alpha-1 isoform X1	83 kDa	0.0148
543	mitochondrial trifunctional protein, alpha subunit	83 kDa	0.0147
544	reticulon-1 isoform 1	83 kDa	0.0147
545	PREDICTED: choline transporter-like protein 1 isoform X2	83 kDa	0.0146
546	probable hydrolase PNKD	41 kDa	0.0146
547	thromboxane-A synthase	42 kDa	0.0145
548	PREDICTED: reticulon-1 isoform X1	85 kDa	0.0144
549	PREDICTED: peroxisomal membrane protein PEX14	43 kDa	0.0141
550	solute carrier family 43 member 3	43 kDa	0.0139
551	acyl-CoA:lysophosphatidylglycerol acyltransferase 1	43 kDa	0.0139
552	reticulon-1 isoform 1	88 kDa	0.0139
553	monoacylglycerol lipase ABHD12	44 kDa	0.0138
554	PREDICTED: protein EFR3 homolog B isoform X2	91 kDa	0.0133
555	26S protease regulatory subunit 8	46 kDa	0.0131
556	PREDICTED: dyslexia-associated protein KIAA0319-like protein homolog isoform X1	93 kDa	0.0131
557	PREDICTED: protein NDRG3 isoform X1	46 kDa	0.013
558	PREDICTED: butyrophilin subfamily 3 member A1-like	46 kDa	0.013
559	PREDICTED: regulator of microtubule dynamics protein 2 isoform X1	47 kDa	0.0129
560	saccharopine dehydrogenase b	47 kDa	0.0128
561	ectonucleotide pyrophosphatase/phosphodiesterase family member 6 precursor	47 kDa	0.0127
562	26S proteasome non-ATPase regulatory subunit 3	48 kDa	0.0126
563	PREDICTED: required for meiotic nuclear division protein 1 homolog isoform X1	48 kDa	0.0125
564	PREDICTED: basic leucine zipper and W2 domain-containing protein 1-A	48 kDa	0.0125
565	palmitoyltransferase ZDHHC17	48 kDa	0.0124
566	PREDICTED: ubiquinol-cytochrome c reductase core protein II isoform X1	49 kDa	0.0124
567	PREDICTED: protein GPR108	49 kDa	0.0123
568	PREDICTED: calcium-binding mitochondrial carrier protein SCaMC-2-B isoform X2	49 kDa	0.0123
569	TLD domain-containing protein 1	50 kDa	0.0121
570	PREDICTED: LOW QUALITY PROTEIN: long-chain-fatty-acid--CoA ligase 6	50 kDa	0.0121
571	PREDICTED: oxysterol-binding protein-related protein 1-like	50 kDa	0.012
572	AP-2 complex subunit mu-A isoform 1	50 kDa	0.012
573	PREDICTED: uncharacterized protein si:ch211-11k18.4	51 kDa	0.0119
574	peroxisomal multifunctional enzyme type 2	51 kDa	0.0118
575	dihydrolipoyllysine-residue succinyltransferase component of 2-oxoglutarate dehydrogenase complex, mitochondrial	51 kDa	0.0117
576	cytochrome P450, family 20, subfamily A, polypeptide 1	52 kDa	0.0116
577	PREDICTED: V-type proton ATPase subunit S1	52 kDa	0.0116
578	citrate synthase, mitochondrial precursor	52 kDa	0.0116
579	epoxide hydrolase 1	52 kDa	0.0115
580	cytochrome b-c1 complex subunit 1, mitochondrial	52 kDa	0.0115

581	mitochondrial dynamics protein MID49	52 kDa	0.0115
582	ectonucleotide pyrophosphatase/phosphodiesterase family member 5 precursor	53 kDa	0.0114
583	PREDICTED: sodium-coupled neutral amino acid transporter 3	53 kDa	0.0113
584	NADH dehydrogenase	53 kDa	0.0113
585	PREDICTED: S-arrestin	53 kDa	0.0112
586	PREDICTED: abhydrolase domain-containing protein 8	53 kDa	0.0112
587	PREDICTED: protein LYRIC isoform X1	53 kDa	0.0112
588	PREDICTED: microtubule-associated protein futsch-like	217 kDa	0.0112
589	zinc transporter 1	54 kDa	0.0112
590	dihydrolipoyl dehydrogenase, mitochondrial	54 kDa	0.0112
591	PREDICTED: probable ATP-dependent RNA helicase ddx6-like isoform X2	54 kDa	0.011
592	V-type proton ATPase subunit H isoform 1	55 kDa	0.0109
593	T-complex protein 11-like protein 1	56 kDa	0.0107
594	6-phosphogluconate dehydrogenase, decarboxylating isoform 1	56 kDa	0.0107
595	dol-P-Man:Man(7)GlcNAc(2)-PP-Dol alpha-1,6-mannosyltransferase precursor	56 kDa	0.0106
596	GDP-Man:Man(3)GlcNAc(2)-PP-Dol alpha-1,2-mannosyltransferase isoform 1	57 kDa	0.0106
597	PREDICTED: peptidyl-prolyl cis-trans isomerase FKBP8 isoform X1	57 kDa	0.0105
598	PREDICTED: protein phosphatase 3, catalytic subunit, gamma isoform-like isoform X1	57 kDa	0.0104
599	T-complex protein 1 subunit zeta	58 kDa	0.0104
600	PREDICTED: protein phosphatase 3, catalytic subunit, gamma isoform isoform X1	58 kDa	0.0104
601	PREDICTED: cell adhesion molecule 3 isoform X1	58 kDa	0.0102
602	PREDICTED: glycerol kinase isoform X4	59 kDa	0.0102
603	PREDICTED: serine/threonine-protein phosphatase 2A 56 kDa regulatory subunit alpha isoform	59 kDa	0.0102
604	amine oxidase	59 kDa	0.0102
605	PREDICTED: lysophosphatidylcholine acyltransferase 1-like	59 kDa	0.0102
606	PREDICTED: peroxisomal N(1)-acetyl-spermine/spermidine oxidase	59 kDa	0.0101
607	T-complex protein 1 subunit epsilon	59 kDa	0.0101
608	PREDICTED: voltage-dependent calcium channel subunit alpha-2/delta-1-like	120 kDa	0.01
609	uncharacterized protein LOC619266 precursor	60 kDa	0.01
610	PREDICTED: calcium-activated potassium channel subunit alpha-1 isoform X15	60 kDa	0.01
611	PREDICTED: cyclin-dependent kinase 17 isoform X1	60 kDa	0.01
612	EH-domain containing 1a	60 kDa	0.01
613	non-specific lipid-transfer protein	60 kDa	0.0099
614	methylmalonate-semialdehyde dehydrogenase	61 kDa	0.0099
615	PREDICTED: brain-specific angiogenesis inhibitor 1-associated protein 2 isoform X1	61 kDa	0.0098
616	delta-1-pyrroline-5-carboxylate dehydrogenase, mitochondrial precursor	62 kDa	0.0097
617	zinc transporter 9	64 kDa	0.0094
618	PREDICTED: acetolactate synthase-like protein isoform X1	67 kDa	0.0089
619	PREDICTED: sodium/potassium/calcium exchanger 2-like isoform X2	68 kDa	0.0088
620	chaperone activity of bc1 complex-like, mitochondrial	70 kDa	0.0086
621	cleft lip and palate transmembrane protein 1 homolog	72 kDa	0.0083
622	78 kDa glucose-regulated protein precursor	72 kDa	0.0083
623	PREDICTED: LOW QUALITY PROTEIN: long-chain-fatty-acid--CoA ligase 6	75 kDa	0.008
624	transmembrane 9 superfamily member 4 precursor	75 kDa	0.0079
625	PREDICTED: protein PTHB1 isoform X1	76 kDa	0.0079
626	sphingomyelin phosphodiesterase 3	76 kDa	0.0078
627	ATP-binding cassette sub-family B member 8, mitochondrial	77 kDa	0.0078
628	PREDICTED: potassium/sodium hyperpolarization-activated cyclic nucleotide-gated channel 3-like	79 kDa	0.0075
629	dolichyl-diphosphooligosaccharide--protein glycosyltransferase subunit STT3A	81 kDa	0.0074
630	disintegrin and metalloproteinase domain-containing protein 10 precursor	85 kDa	0.007
631	aconitate hydratase, mitochondrial	86 kDa	0.007
632	PREDICTED: ATP-dependent 6-phosphofructokinase, platelet type isoform X2	86 kDa	0.0069
633	PREDICTED: retinal-specific ATP-binding cassette transporter isoform X2	261 kDa	0.0069
634	heat shock protein HSP 90-beta	89 kDa	0.0067
635	PREDICTED: paraplegin	92 kDa	0.0065
636	PREDICTED: 2-oxoglutarate dehydrogenase, mitochondrial	93 kDa	0.0064
637	PREDICTED: prominin-1-A isoform X10	94 kDa	0.0064
638	PREDICTED: oxysterol-binding protein-related protein 8-like	98 kDa	0.0061

639	cone cGMP-specific 3',5'-cyclic phosphodiesterase subunit alpha'	98 kDa	0.0061
640	PREDICTED: cleavage and polyadenylation specificity factor subunit 1 isoform X1	99 kDa	0.006
641	PREDICTED: bifunctional heparan sulfate N-deacetylase/N-sulfotransferase 1	102 kDa	0.0058
642	PREDICTED: transmembrane and TPR repeat-containing protein 3	105 kDa	0.0057
643	PREDICTED: AP-1 complex subunit beta-1 isoform X2	107 kDa	0.0055
644	sorting nexin-25	111 kDa	0.0054
645	PREDICTED: sorting nexin-14 isoform X2	113 kDa	0.0053
646	PREDICTED: uncharacterized protein KIAA1614 homolog isoform X1	115 kDa	0.0052
647	PREDICTED: solute carrier family 12 member 7 isoform X5	121 kDa	0.0049
648	PREDICTED: oxygen-regulated protein 1	232 kDa	0.0026
