# Supplementary material for: Purification of cone outer segment for proteomic analysis on its membrane proteins in carp retina
Source: PLoS One. 2017 Mar 14;12(3):e0173908. doi: 10.1371/journal.pone.0173908 (PMC5349680; doi:10.1371/journal.pone.0173908)
Supplement: S2 Table — Proteins in washed RIS-rich fraction were identified with LC-MS/MS analysis and are listed in descending order of emPAI values for 5 × 10^5^ rods. (PDF) [file pone.0173908.s002.pdf]

S2 Table. Identified proteins in washed RIS-rich fraction. Proteins in washed RIS-rich fraction were identified with LC-MS/MS analysis and are listed in descending order of emPAI values for 5×10^5 rods.

	Identified proteins in washed RIS-rich fraction	Molecular mass	emPAI
1	uncharacterized protein LOC100145214	33 kDa	733.05
2	ATP synthase F(0) complex subunit B1, mitochondrial	31 kDa	21.616
3	voltage-dependent anion-selective channel protein 1	31 kDa	11.403
4	PREDICTED: ATP synthase subunit alpha, mitochondrial	27 kDa	8.7106
5	creatine kinase S-type, mitochondrial	47 kDa	8.4939
6	PREDICTED: creatine kinase S-type, mitochondrial isoform X2	27 kDa	8.4926
7	PREDICTED: uncharacterized protein LOC100707031 isoform X1	12 kDa	8.3582
8	PREDICTED: creatine kinase S-type, mitochondrial isoform X2	47 kDa	7.7687
9	voltage-dependent anion-selective channel protein 2	30 kDa	6.5886
10	PREDICTED: sema domain, immunoglobulin domain (Ig), short basic domain, secreted, (semaphorin) 3Fa isoform X2	40 kDa	6.4744
11	PREDICTED: prohibitin isoform X2	22 kDa	5.7014
12	ATP synthase subunit g, mitochondrial	11 kDa	5.4627
13	creatine kinase S-type, mitochondrial	11 kDa	3.4905
14	serine/threonine-protein kinase MAK	11 kDa	3.2093
15	prohibitin 2a	35 kDa	2.9052
16	PREDICTED: prohibitin	22 kDa	2.8865
17	HIG1 domain family member 1A	11 kDa	2.8453
18	cytochrome b-c1 complex subunit 1, mitochondrial	52 kDa	2.818
19	PREDICTED: solute carrier family 25 (mitochondrial carrier; phosphate carrier), member 3a isoform X2	40 kDa	2.6469
20	voltage-dependent anion-selective channel protein 2-like	30 kDa	2.3697
21	PREDICTED: retinol dehydrogenase 8-like isoform X1	35 kDa	2.3033
22	mitochondrial 2-oxoglutarate/malate carrier protein	38 kDa	2.2404
23	malate dehydrogenase, mitochondrial	35 kDa	1.9284
24	PREDICTED: creatine kinase S-type, mitochondrial isoform X2	17 kDa	1.8764
25	PREDICTED: NADH dehydrogenase	15 kDa	1.8277
26	NADH dehydrogenase 1 beta subcomplex subunit 4	15 kDa	1.7781
27	ATP synthase subunit d, mitochondrial	18 kDa	1.6005
28	calcium-binding mitochondrial carrier protein Aralar1	76 kDa	1.4904
29	prohibitin 2-like	27 kDa	1.4654
30	ATP synthase subunit alpha, mitochondrial	33 kDa	1.404
31	ATP synthase subunit O, mitochondrial	26 kDa	1.3261
32	ADP/ATP translocase 3	27 kDa	1.2792
33	PREDICTED: NADH dehydrogenase	20 kDa	1.2519
34	PREDICTED: NADH dehydrogenase	19 kDa	1.2519
35	calcium-binding mitochondrial carrier protein Aralar1	60 kDa	1.1102
36	PREDICTED: hexokinase-1-like	14 kDa	1.0688
37	cytochrome c oxidase subunit 4 isoform 1, mitochondrial	20 kDa	1.0395
38	PREDICTED: NADH dehydrogenase	12 kDa	1.0008
39	PREDICTED: ATP synthase subunit gamma, mitochondrial isoform X1	33 kDa	0.9213
40	mitochondrial import receptor subunit TOM40 homolog	36 kDa	0.8869
41	isocitrate dehydrogenase	50 kDa	0.8761
42	PREDICTED: voltage-dependent anion-selective channel protein 2	11 kDa	0.8756
43	PREDICTED: prohibitin-2	27 kDa	0.8586
44	PREDICTED: voltage-dependent anion-selective channel protein 2	11 kDa	0.85
45	dihydrolipoyllysine-residue acetyltransferase component of pyruvate dehydrogenase complex, mitochondrial	69 kDa	0.7958
46	CDGSH iron sulfur domain 1	12 kDa	0.7186
47	PREDICTED: tubulin beta-4B chain-like	31 kDa	0.7144
48	PREDICTED: voltage-dependent anion-selective channel protein 3 isoform X3	36 kDa	0.7131
49	uncharacterized protein LOC541492	13 kDa	0.7088
50	PREDICTED: coiled-coil-helix-coiled-coil-helix domain-containing protein 6, mitochondrial isoform X2	29 kDa	0.707
51	mitochondrial import receptor subunit TOM40 homolog	36 kDa	0.7066

52	PREDICTED: NADH dehydrogenase	25 kDa	0.6935
53	PREDICTED: LOW QUALITY PROTEIN: actin, gamma 1	40 kDa	0.6866
54	ATPase, Na+/K+ transporting, beta 2b polypeptide	34 kDa	0.68
55	PREDICTED: pyruvate dehydrogenase E1 alpha 1 isoform X1	45 kDa	0.6756
56	S-arrestin	44 kDa	0.6623
57	PREDICTED: voltage-dependent anion-selective channel protein 3 isoform X3	36 kDa	0.642
58	PREDICTED: ATP synthase subunit beta, mitochondrial-like	56 kDa	0.6347
59	hexokinase-1	11 kDa	0.6334
60	sorting and assembly machinery component 50 homolog B	23 kDa	0.6189
61	PREDICTED: calcium-binding mitochondrial carrier protein Aralar2 isoform X1	15 kDa	0.6177
62	PREDICTED: L-lactate dehydrogenase B-B chain isoform X3	19 kDa	0.5989
63	PREDICTED: NADH dehydrogenase	14 kDa	0.591
64	uncharacterized protein LOC100135302	20 kDa	0.5895
65	PREDICTED: NADH dehydrogenase	17 kDa	0.5848
66	guanine nucleotide-binding protein G(I)/G(S)/G(T) subunit beta-1	37 kDa	0.5839
67	NADH dehydrogenase 1 alpha subcomplex subunit 11	16 kDa	0.5819
68	mitochondrial import receptor subunit TOM70	65 kDa	0.5705
69	PREDICTED: mitochondrial pyruvate carrier 2	14 kDa	0.5548
70	guanine nucleotide-binding protein G(I)/G(S)/G(T) subunit beta-1	37 kDa	0.531
71	sorting and assembly machinery component 50 homolog A	52 kDa	0.5031
72	NADH dehydrogenase 1 beta subcomplex subunit 6	15 kDa	0.5021
73	PREDICTED: threonine dehydratase, mitochondrial-like isoform X2	60 kDa	0.4921
74	NADH dehydrogenase 1 alpha subcomplex subunit 6	16 kDa	0.4489
75	NADH dehydrogenase (ubiquinone) 1 subunit c2	13 kDa	0.4395
76	aspartate aminotransferase 2a	48 kDa	0.4383
77	cytochrome c-1	36 kDa	0.4087
78	putative tubulin beta chain variant 1	20 kDa	0.3935
79	voltage-dependent anion-selective channel protein 2	20 kDa	0.3776
80	uncharacterized protein LOC100135302	23 kDa	0.3768
81	NADH-ubiquinone oxidoreductase 75 kDa subunit, mitochondrial	80 kDa	0.3711
82	mitochondrial NADH dehydrogenase (ubiquinone) 1 beta subcomplex subunit 5	22 kDa	0.3702
83	mitochondrial chaperone BCS1	48 kDa	0.3687
84	PREDICTED: ATPase, Na+/K+ transporting, beta 2b polypeptide isoform X1	24 kDa	0.3639
85	cytochrome b-c1 complex subunit 2, mitochondrial	50 kDa	0.3558
86	aspartate aminotransferase 2	43 kDa	0.3536
87	NADH dehydrogenase	41 kDa	0.3475
88	NADH dehydrogenase	31 kDa	0.3435
89	sodium/potassium-transporting ATPase subunit alpha-3	113 kDa	0.3395
90	PREDICTED: phosphate carrier protein, mitochondrial-like isoform X1	34 kDa	0.3353
91	PREDICTED: threo-3-hydroxyaspartate ammonia-lyase-like	41 kDa	0.332
92	NADH dehydrogenase	53 kDa	0.329
93	cytochrome c oxidase subunit 4 isoform 1, mitochondrial	20 kDa	0.3265
94	plasminogen receptor (KT)	20 kDa	0.3223
95	sideroflexin-3	36 kDa	0.311
96	PREDICTED: peroxiredoxin-5, mitochondrial-like	20 kDa	0.306
97	PREDICTED: calcium-binding mitochondrial carrier protein Aralar2 isoform X1	48 kDa	0.3028
98	NADH dehydrogenase	58 kDa	0.2958
99	solute carrier family 25, member 23	31 kDa	0.2892
100	NAD(P) transhydrogenase, mitochondrial	114 kDa	0.2842
101	PREDICTED: vesicle-associated membrane protein-associated protein A-like	26 kDa	0.283
102	PREDICTED: vesicle-associated membrane protein-associated protein A-like	26 kDa	0.283
103	stomatin-like protein 2, mitochondrial	39 kDa	0.2785
104	PREDICTED: sodium/potassium-transporting ATPase subunit alpha-3-like	27 kDa	0.2751
105	stomatin-like protein 2, mitochondrial	39 kDa	0.275
106	PREDICTED: pyruvate dehydrogenase E1 component subunit alpha, somatic form, mitochondrial isoform X1	25 kDa	0.2739
107	PREDICTED: ubiquinol-cytochrome c reductase core protein II isoform X1	49 kDa	0.2723
108	LETM1 and EF-hand domain-containing protein 1, mitochondrial	86 kDa	0.2666
109	protein disulfide-isomerase TMX3 precursor	47 kDa	0.2645
110	mitochondrial inner membrane protein	83 kDa	0.2592

111	reticulon-4-interacting protein 1 homolog, mitochondrial	42 kDa	0.2566
112	mitochondrial import inner membrane translocase subunit Tim17-B	18 kDa	0.2565
113	protein NipSnap homolog 2	33 kDa	0.2554
114	voltage-dependent anion-selective channel protein 2	20 kDa	0.2532
115	mitochondrial pyruvate carrier 1	13 kDa	0.2434
116	solute carrier family 25, member 23	23 kDa	0.2278
117	citrate synthase, mitochondrial precursor	52 kDa	0.2185
118	apolipoprotein O	24 kDa	0.2167
119	cytochrome c oxidase subunit VIIa polypeptide 3	16 kDa	0.2071
120	PREDICTED: mitochondrial glutamate carrier 1	34 kDa	0.2071
121	cytochrome c oxidase subunit Vaa	16 kDa	0.2042
122	PREDICTED: tubulin alpha chain-like	12 kDa	0.2021
123	dihydrolipoyl dehydrogenase, mitochondrial	54 kDa	0.2018
124	hexokinase-1	54 kDa	0.2009
125	ES1 protein, mitochondrial precursor	31 kDa	0.2005
126	transmembrane protein 256 precursor	12 kDa	0.1984
127	PREDICTED: ubiquinol-cytochrome c reductase core protein II isoform X1	40 kDa	0.1957
128	PREDICTED: uncharacterized protein LOC571872 isoform X1	12 kDa	0.1948
129	NADH dehydrogenase	12 kDa	0.1913
130	alpha-enolase	47 kDa	0.186
131	PREDICTED: NADH dehydrogenase	21 kDa	0.1825
132	PREDICTED: apoptosis-inducing factor 1, mitochondrial isoform X3	68 kDa	0.181
133	succinyl-CoA ligase	51 kDa	0.1776
134	PREDICTED: glutaryl-CoA dehydrogenase, mitochondrial-like	49 kDa	0.1759
135	PREDICTED: apoptosis-inducing factor 1, mitochondrial isoform X1	48 kDa	0.1759
136	NADH dehydrogenase	13 kDa	0.1755
137	mitochondrial carrier homolog 2	31 kDa	0.1739
138	heat shock cognate 71 kDa protein	42 kDa	0.1735
139	isocitrate dehydrogenase	40 kDa	0.1722
140	erlin-2 precursor	40 kDa	0.1713
141	cytochrome c oxidase subunit 6A1, mitochondrial	13 kDa	0.1698
142	enoyl-CoA hydratase, mitochondrial	31 kDa	0.1691
143	solute carrier family 25, member 23	31 kDa	0.1691
144	PREDICTED: LOW QUALITY PROTEIN: long-chain-fatty-acid--CoA ligase 6	50 kDa	0.1667
145	isocitrate dehydrogenase	43 kDa	0.1665
146	PREDICTED: NADH dehydrogenase	43 kDa	0.1648
147	L-lactate dehydrogenase B-A chain	36 kDa	0.1643
148	PREDICTED: isocitrate dehydrogenase	44 kDa	0.1624
149	PREDICTED: cytochrome b-c1 complex subunit 7-like	13 kDa	0.1619
150	pyruvate dehydrogenase E1 component subunit beta, mitochondrial	39 kDa	0.1589
151	translocase of outer mitochondrial membrane 40 homolog, like	35 kDa	0.1584
152	methylmalonate-semialdehyde dehydrogenase	61 kDa	0.1579
153	PREDICTED: uncharacterized protein LOC556653	23 kDa	0.1577
154	NADH-cytochrome b5 reductase 1	28 kDa	0.1561
155	rhodopsin	40 kDa	0.1555
156	PREDICTED: tubulin alpha-1C chain	45 kDa	0.1547
157	hexokinase-1	71 kDa	0.1528
158	uncharacterized protein LOC556781	23 kDa	0.1521
159	isocitrate dehydrogenase	43 kDa	0.152
160	3-hydroxyacyl-CoA dehydrogenase type-2	28 kDa	0.1504
161	mitochondrial import inner membrane translocase subunit tim16	14 kDa	0.1501
162	PREDICTED: gap junction delta-2 protein-like	21 kDa	0.1495
163	retinol dehydrogenase-like	36 kDa	0.1449
164	PREDICTED: tubulin alpha-4A chain-like, partial	22 kDa	0.1426
165	NADH dehydrogenase	24 kDa	0.1419
166	protein QIL1	12 kDa	0.1404
167	creatine kinase U-type, mitochondrial	22 kDa	0.1399
168	PREDICTED: tubulin beta-2B chain-like isoform 1	55 kDa	0.1394
169	PREDICTED: tubulin alpha chain	45 kDa	0.1388

170	OCIA domain-containing protein 1	30 kDa	0.1381
171	trifunctional enzyme subunit beta, mitochondrial	50 kDa	0.1374
172	reticulon-4	22 kDa	0.1374
173	PREDICTED: mitochondrial import inner membrane translocase subunit Tim23	22 kDa	0.1349
174	PREDICTED: protein FAM162B isoform X1	17 kDa	0.1314
175	PREDICTED: complex I assembly factor TIMMDC1, mitochondrial isoform X1	33 kDa	0.1305
176	translocase of outer mitochondrial membrane 40 homolog, like	36 kDa	0.1302
177	ATPase family AAA domain-containing protein 3	69 kDa	0.1242
178	protein NDRG1 isoform 1	42 kDa	0.1242
179	succinate dehydrogenase	61 kDa	0.1235
180	ras-related protein Rab-2A	24 kDa	0.1227
181	uncharacterized protein LOC556781	13 kDa	0.1218
182	succinate dehydrogenase	73 kDa	0.1212
183	PREDICTED: FAS-associated factor 2-like isoform X1	54 kDa	0.1196
184	PREDICTED: presenilins-associated rhomboid-like protein, mitochondrial	41 kDa	0.1181
185	PREDICTED: LOW QUALITY PROTEIN: long-chain-fatty-acid--CoA ligase 6	75 kDa	0.1152
186	mitochondrial import inner membrane translocase subunit Tim23	22 kDa	0.1127
187	PREDICTED: arylacetamide deacetylase isoform X2	16 kDa	0.1101
188	PREDICTED: ras-related protein Rab-1A-like isoform X1	22 kDa	0.1089
189	ATPase family AAA domain-containing protein 3	69 kDa	0.1088
190	coiled-coil-helix-coiled-coil-helix domain-containing protein 3, mitochondrial	37 kDa	0.1085
191	ras-related protein Rab-1B	22 kDa	0.108
192	Tubulin beta-2C chain	11 kDa	0.1069
193	retinol dehydrogenase 13	37 kDa	0.1068
194	PREDICTED: calcium-binding mitochondrial carrier protein SCaMC-2-B isoform X2	49 kDa	0.1065
195	NADH dehydrogenase	17 kDa	0.1065
196	synaptosomal-associated protein 25-B	23 kDa	0.1063
197	aconitate hydratase, mitochondrial	86 kDa	0.1031
198	PREDICTED: tubulin alpha chain-like	50 kDa	0.1025
199	PREDICTED: uncharacterized protein LOC571872 isoform X1	32 kDa	0.1009
200	mitochondrial import inner membrane translocase subunit Tim17-A	17 kDa	0.1009
201	uncharacterized protein LOC541537	27 kDa	0.0992
202	PREDICTED: uncharacterized protein LOC101885612	11 kDa	0.0971
203	PREDICTED: actin, aortic smooth muscle	18 kDa	0.0968
204	mitochondria-eating protein	55 kDa	0.096
205	28S ribosomal protein S36, mitochondrial	12 kDa	0.0957
206	brain creatine kinase b	43 kDa	0.0955
207	sorting and assembly machinery component 50 homolog B	27 kDa	0.0953
208	PREDICTED: NADH dehydrogenase	21 kDa	0.0942
209	elongation factor Tu, mitochondrial	49 kDa	0.0942
210	PREDICTED: mitochondrial fission process protein 1-like	18 kDa	0.0939
211	transmembrane protein 256 precursor	12 kDa	0.0928
212	glutaryl-CoA dehydrogenase a	18 kDa	0.0912
213	sideroflexin-4	35 kDa	0.0909
214	PREDICTED: vesicle-associated membrane protein 2-like	12 kDa	0.0902
215	acylglycerol kinase, mitochondrial precursor	48 kDa	0.0901
216	ras-related protein Rab-3A	28 kDa	0.0898
217	long-chain fatty acid transport protein 4	25 kDa	0.0897
218	histone 1, H4, like	12 kDa	0.0889
219	mitochondrial ubiquitin ligase activator of nfkb 1-A	12 kDa	0.0889
220	PREDICTED: succinate dehydrogenase cytochrome b560 subunit, mitochondrial isoform X1	19 kDa	0.0886
221	PREDICTED: ornithine aminotransferase, mitochondrial	49 kDa	0.0871
222	erlin-1 precursor	39 kDa	0.0859
223	PREDICTED: L-lactate dehydrogenase B-B chain isoform X4	19 kDa	0.0846
224	PREDICTED: S-arrestin	53 kDa	0.0833
225	PREDICTED: arylacetamide deacetylase isoform X1	47 kDa	0.0829
226	cysteine desulfurase, mitochondrial	50 kDa	0.0828
227	PREDICTED: mitochondrial ubiquitin ligase activator of nfkb 1-A	33 kDa	0.0823
228	PREDICTED: ATP-binding cassette sub-family B member 7, mitochondrial	82 kDa	0.0811

229	PREDICTED: stress-70 protein, mitochondrial-like, partial	17 kDa	0.0784
230	ATP-binding cassette sub-family B member 8, mitochondrial	77 kDa	0.0781
231	PREDICTED: cadherin-related family member 5-like isoform X2	20 kDa	0.0776
232	inactive hydroxysteroid dehydrogenase-like protein 1	35 kDa	0.077
233	PREDICTED: growth hormone-inducible transmembrane protein	35 kDa	0.0762
234	erlin-1 precursor	39 kDa	0.0753
235	solute carrier family 25 (mitochondrial carrier: glutamate), member 22	36 kDa	0.0737
236	PREDICTED: malate dehydrogenase 1Aa, NAD (soluble) isoform X1	36 kDa	0.072
237	dihydrolipoyllysine-residue succinyltransferase component of 2-oxoglutarate dehydrogenase complex, mitochondrial	51 kDa	0.0714
238	PREDICTED: calcium-binding mitochondrial carrier protein SCaMC-3-like isoform X1	10 kDa	0.0708
239	PREDICTED: magnesium transporter MRS2 homolog, mitochondrial isoform X1	48 kDa	0.0702
240	vesicle-associated membrane protein-associated protein A	30 kDa	0.0696
241	catechol-O-methyltransferase a	30 kDa	0.0692
242	NADH dehydrogenase (ubiquinone) Fe-S protein 8b	22 kDa	0.0689
243	probable D-lactate dehydrogenase, mitochondrial	53 kDa	0.0687
244	mitochondrial carnitine/acylcarnitine carrier protein CACL	34 kDa	0.0682
245	ADP-ribosylation factor-like protein 9	26 kDa	0.0682
246	PREDICTED: sodium/potassium-transporting ATPase subunit beta-2-like	19 kDa	0.0674
247	PREDICTED: transmembrane protein 126A isoform X1	22 kDa	0.0673
248	PREDICTED: mitochondrial import inner membrane translocase subunit Tim21	27 kDa	0.0656
249	Na+/K+ -ATPase alpha 1 subunit	90 kDa	0.0651
250	PREDICTED: uncharacterized protein LOC556653	35 kDa	0.0647
251	PREDICTED: transmembrane emp24 domain-containing protein 9 isoform X1	27 kDa	0.0644
252	acetyl-CoA acetyltransferase, mitochondrial precursor	48 kDa	0.0628
253	RAB5A, member RAS oncogene family, a	24 kDa	0.0627
254	stress-70 protein, mitochondrial	73 kDa	0.0615
255	very-long-chain enoyl-CoA reductase	36 kDa	0.0615
256	cytochrome c	11 kDa	0.0613
257	metaxin 1	36 kDa	0.0612
258	cytochrome c oxidase subunit IV isoform 2	20 kDa	0.0604
259	PREDICTED: coiled-coil domain-containing protein 136-like isoform X1	53 kDa	0.0602
260	heat shock cognate 71 kDa protein	29 kDa	0.0597
261	protein QIL1	12 kDa	0.0597
262	ATPase, Na+/K+ transporting, beta 2a polypeptide	16 kDa	0.0595
263	PREDICTED: ADP-dependent glucokinase isoform X2	58 kDa	0.0591
264	synaptojanin-2-binding protein	16 kDa	0.0578
265	pyrroline-5-carboxylate reductase 1a	34 kDa	0.0574
266	zinc transporter 9	64 kDa	0.0573
267	PREDICTED: protein SCO1 homolog, mitochondrial	34 kDa	0.0568
268	PREDICTED: dnaJ homolog subfamily C member 11	65 kDa	0.0557
269	PREDICTED: mitochondrial fission factor homolog B isoform X1	34 kDa	0.0555
270	PREDICTED: glutaminase a isoform X1	66 kDa	0.0549
271	calmegin precursor	66 kDa	0.0548
272	COX16 cytochrome c oxidase assembly homolog	13 kDa	0.0546
273	PREDICTED: peptidyl-prolyl cis-trans isomerase FKBP8 isoform X1	57 kDa	0.0545
274	mitochondrial import inner membrane translocase subunit TIM50 precursor	44 kDa	0.054
275	mitochondrial ATP synthase subunit f	13 kDa	0.054
276	60S ribosomal protein L18	22 kDa	0.0539
277	traB domain-containing protein	40 kDa	0.0534
278	probable glutamate--tRNA ligase, mitochondrial precursor	40 kDa	0.0534
279	metaxin-2	31 kDa	0.0531
280	transmembrane protein 11, mitochondrial	27 kDa	0.0528
281	reticulon-4	22 kDa	0.0528
282	78 kDa glucose-regulated protein precursor	72 kDa	0.0527
283	glyceraldehyde-3-phosphate dehydrogenase 2	36 kDa	0.0521
284	60S ribosomal protein L14	18 kDa	0.0518
285	60S ribosomal protein L24	18 kDa	0.0514
286	RAB1A, member RAS oncogene family	28 kDa	0.0496
287	ATP synthase F(0) complex subunit C3, mitochondrial	14 kDa	0.0487

288	serine hydroxymethyltransferase, mitochondrial	57 kDa	0.0483
289	thioredoxin-dependent peroxide reductase, mitochondrial	28 kDa	0.0482
290	60 kDa heat shock protein, mitochondrial	28 kDa	0.048
291	ras-related protein Rab-1B	19 kDa	0.0477
292	acyl-CoA synthetase long-chain family member 3b	79 kDa	0.0464
293	PREDICTED: nuclease EXOG, mitochondrial	39 kDa	0.0464
294	mitochondrial Rho GTPase 2	70 kDa	0.0457
295	uncharacterized protein LOC619266 precursor	60 kDa	0.0455
296	DDRGK domain-containing protein 1 precursor	35 kDa	0.0454
297	PREDICTED: ras-related protein Rab-11B	25 kDa	0.0453
298	PREDICTED: ER membrane protein complex subunit 1 isoform X1	111 kDa	0.045
299	guanine nucleotide-binding protein G(o) subunit alpha	40 kDa	0.045
300	PREDICTED: syntaxin-12 isoform X1	30 kDa	0.0444
301	RAB11a, member RAS oncogene family, like	20 kDa	0.0438
302	PREDICTED: NADH dehydrogenase	20 kDa	0.0438
303	mitochondrial trifunctional protein, alpha subunit	83 kDa	0.0434
304	phosphatidate cytidylyltransferase, mitochondrial precursor	15 kDa	0.0434
305	solute carrier family 3 (amino acid transporter heavy chain), member 2b	57 kDa	0.0432
306	AFG3-like protein 2	89 kDa	0.043
307	glycerophosphodiester phosphodiesterase domain-containing protein 1	36 kDa	0.0428
308	immunity-related GTPase family, q2	42 kDa	0.0423
309	40S ribosomal protein S25	15 kDa	0.0422
310	Beta-centractin	15 kDa	0.0418
311	PREDICTED: ADP-dependent glucokinase isoform X2	26 kDa	0.0417
312	PREDICTED: OCIA domain-containing protein 1 isoform X1	26 kDa	0.0417
313	PREDICTED: mitochondrial fission factor-like isoform X3	26 kDa	0.0417
314	mitochondrial dicarboxylate carrier	32 kDa	0.0416
315	PREDICTED: kynurenine/alpha-aminoadipate aminotransferase, mitochondrial isoform X1	48 kDa	0.0415
316	oxoglutarate (alpha-ketoglutarate) dehydrogenase (lipoamide)	21 kDa	0.0413
317	ras-related protein Rab-5C	37 kDa	0.0411
318	surfeit locus protein 1	32 kDa	0.0411
319	PREDICTED: succinate dehydrogenase	32 kDa	0.0411
320	PREDICTED: oxoglutarate (alpha-ketoglutarate) dehydrogenase (lipoamide) isoform X3	26 kDa	0.041
321	PREDICTED: mitochondrial import inner membrane translocase subunit Tim22	21 kDa	0.0407
322	PREDICTED: 2-oxoglutarate dehydrogenase, mitochondrial	93 kDa	0.0406
323	PREDICTED: potassium voltage-gated channel subfamily B member 2	93 kDa	0.0403
324	c3orf33 homolog	16 kDa	0.0395
325	rhodopsin kinase	27 kDa	0.0395
326	PREDICTED: oxoglutarate (alpha-ketoglutarate) dehydrogenase (lipoamide) isoform X2	44 kDa	0.0394
327	PREDICTED: NADH dehydrogenase (ubiquinone) complex I, assembly factor 6	33 kDa	0.0394
328	PREDICTED: transmembrane emp24 domain-containing protein 7 isoform X2	27 kDa	0.0391
329	PREDICTED: FUN14 domain-containing protein 2 isoform X1	16 kDa	0.0388
330	eukaryotic translation elongation factor 1 alpha 1-like	50 kDa	0.0387
331	ATPase family AAA domain-containing protein 1-B	22 kDa	0.0386
332	immunoglobulin superfamily member 8 precursor	68 kDa	0.0386
333	mitochondrial import receptor subunit TOM20 homolog B	16 kDa	0.0385
334	uncharacterized protein C2orf47 homolog, mitochondrial	28 kDa	0.0379
335	dolichyl-diphosphooligosaccharide--protein glycosyltransferase 48 kDa subunit precursor	51 kDa	0.0377
336	guanine nucleotide-binding protein G(t) subunit alpha-2	40 kDa	0.0372
337	ATP synthase subunit delta, mitochondrial	17 kDa	0.0372
338	PREDICTED: mitochondrial pyruvate carrier 2-like	17 kDa	0.0372
339	protein disulfide-isomerase TMX3 precursor	52 kDa	0.0371
340	uncharacterized protein LOC100127828	34 kDa	0.0371
341	PREDICTED: transmembrane emp24 domain-containing protein 2	23 kDa	0.0369
342	uncharacterized protein LOC100145220	29 kDa	0.0367
343	ubiquinone biosynthesis monooxygenase COQ6	52 kDa	0.0366

344	phosphatidylglycerophosphatase and protein-tyrosine phosphatase 1	11 kDa	0.0364
345	PREDICTED: probable UDP-sugar transporter protein SLC35A4 isoform X1	11 kDa	0.0364
346	60 kDa heat shock protein, mitochondrial	29 kDa	0.0362
347	mitochondrial folate transporter/carrier	35 kDa	0.0362
348	phosphatidate cytidylyltransferase, mitochondrial precursor	11 kDa	0.0359
349	PREDICTED: sodium/potassium-transporting ATPase subunit alpha-1-like, partial	35 kDa	0.0359
350	surfeit gene 4, like	23 kDa	0.0358
351	PREDICTED: ER membrane protein complex subunit 1 isoform X1	114 kDa	0.0355
352	PREDICTED: heme oxygenase 2	36 kDa	0.0354
353	PREDICTED: ras-related protein Rab-6A isoform X2	24 kDa	0.0353
354	cytochrome b-c1 complex subunit Rieske, mitochondrial	30 kDa	0.035
355	PREDICTED: ATP-dependent zinc metalloprotease YME1L1 isoform X1	79 kDa	0.0348
356	40S ribosomal protein S19	18 kDa	0.0348
357	PREDICTED: 60S ribosomal protein L6	30 kDa	0.0348
358	metaxin 1a	36 kDa	0.0347
359	ras-related protein Rab-14	24 kDa	0.0347
360	L-2-hydroxyglutarate dehydrogenase, mitochondrial	12 kDa	0.0346
361	optic atrophy 3 protein homolog	18 kDa	0.0345
362	60S ribosomal protein L15	24 kDa	0.0345
363	PREDICTED: sarcolemmal membrane-associated protein isoform X4	92 kDa	0.0344
364	acyl-CoA dehydrogenase-like	49 kDa	0.0343
365	PREDICTED: 40S ribosomal protein S8	24 kDa	0.0343
366	60S ribosomal protein L27a	18 kDa	0.0343
367	vesicle-trafficking protein SEC22b-A	24 kDa	0.0339
368	PREDICTED: saccharopine dehydrogenase-like oxidoreductase-like	12 kDa	0.0331
369	rab GDP dissociation inhibitor beta	51 kDa	0.033
370	mitochondrial trifunctional protein, alpha subunit	38 kDa	0.0328
371	E3 ubiquitin-protein ligase MARCH5	31 kDa	0.0328
372	PREDICTED: LOW QUALITY PROTEIN: 40S ribosomal protein S13-like	19 kDa	0.0327
373	PREDICTED: ER membrane protein complex subunit 6	12 kDa	0.0327
374	succinate dehydrogenase	32 kDa	0.0326
375	COX15 homolog	51 kDa	0.0326
376	rhodopsin kinase	64 kDa	0.0324
377	COX16 cytochrome c oxidase assembly homolog	12 kDa	0.0323
378	mimitin, mitochondrial	19 kDa	0.032
379	dynamin-like 120 kDa protein, mitochondrial precursor	112 kDa	0.0317
380	delta-1-pyrroline-5-carboxylate synthase	85 kDa	0.0316
381	gamma-enolase	52 kDa	0.0316
382	PREDICTED: NCK-interacting protein with SH3 domain-like	19 kDa	0.0313
383	synaptotagmin II	47 kDa	0.0308
384	PREDICTED: regulator of microtubule dynamics protein 2 isoform X1	47 kDa	0.0308
385	PREDICTED: apoptosis-inducing factor 1, mitochondrial isoform X1	13 kDa	0.0306
386	flotillin 2	47 kDa	0.0305
387	dolichyl-diphosphooligosaccharide--protein glycosyltransferase subunit 1 precursor	67 kDa	0.0304
388	NADH dehydrogenase	27 kDa	0.0303
389	PREDICTED: pyruvate dehydrogenase protein X component, mitochondrial	54 kDa	0.0303
390	ATP synthase subunit s, mitochondrial	27 kDa	0.03
391	cytochrome c-type heme lyase	34 kDa	0.0299
392	alpha/beta hydrolase domain-containing protein 11	34 kDa	0.0296
393	nucleoside diphosphate kinase A	20 kDa	0.0294
394	uncharacterized protein LOC100127828	34 kDa	0.0293
395	chaperone activity of bc1 complex-like, mitochondrial	70 kDa	0.0292
396	ras-related protein Rab-35	20 kDa	0.0292
397	very-long-chain (3R)-3-hydroxyacyl-CoA dehydratase 2	28 kDa	0.0287
398	PREDICTED: protein XRP2 isoform X1	42 kDa	0.0287
399	dnaJ homolog subfamily C member 11	64 kDa	0.0285
400	ras-related protein Rap-1b precursor	21 kDa	0.0284
401	fatty aldehyde dehydrogenase	28 kDa	0.0281
402	mitochondrial-processing peptidase subunit alpha	58 kDa	0.028

403	60S ribosomal protein L7	29 kDa	0.0277
404	uncharacterized protein C18orf19 homolog A	29 kDa	0.0276
405	peptidyl-prolyl cis-trans isomerase FKBP8	44 kDa	0.0275
406	histone 2, H2a	14 kDa	0.0274
407	epimerase family protein SDR39U1	36 kDa	0.0274
408	oxidase (cytochrome c) assembly 1-like	51 kDa	0.0274
409	isochorismatase domain-containing protein 2, mitochondrial	21 kDa	0.0274
410	ADP-ribosylation-like factor 6 interacting protein 5	22 kDa	0.0272
411	peptidyl-prolyl cis-trans isomerase FKBP8	44 kDa	0.0271
412	S-adenosylmethionine mitochondrial carrier protein	29 kDa	0.0271
413	protoporphyrinogen oxidase	52 kDa	0.027
414	heat shock protein 75 kDa, mitochondrial	82 kDa	0.027
415	solute carrier family 2, facilitated glucose transporter member 1	53 kDa	0.0263
416	PREDICTED: transmembrane protein 70, mitochondrial	30 kDa	0.0262
417	PREDICTED: 40S ribosomal protein S9-like	22 kDa	0.0259
418	abhydrolase domain-containing protein 16A	38 kDa	0.0257
419	PREDICTED: ras-related protein Rab-10	23 kDa	0.0256
420	adipocyte plasma membrane-associated protein	47 kDa	0.0252
421	PREDICTED: dnaJ homolog subfamily C member 30	47 kDa	0.0252
422	methyltransferase like 7A precursor	31 kDa	0.0251
423	PREDICTED: progestin and adipoQ receptor family member 4-like	31 kDa	0.025
424	NADH dehydrogenase	48 kDa	0.0242
425	PREDICTED: succinyl-CoA ligase	16 kDa	0.0242
426	CDP-diacylglycerol--inositol 3-phosphatidyltransferase	24 kDa	0.0239
427	protein THEM6 precursor	24 kDa	0.0239
428	protein THEM6 precursor	24 kDa	0.0238
429	synaptophysin b isoform 1	33 kDa	0.0235
430	PREDICTED: aspartate beta-hydroxylase isoform X2	33 kDa	0.0233
431	PREDICTED: protein MGARP isoform X2	41 kDa	0.0233
432	ubiquinol-cytochrome c reductase complex assembly factor 1	33 kDa	0.0233
433	fatty-acid amide hydrolase 1	16 kDa	0.0232
434	PREDICTED: reticulon-3-B-like isoform X2	25 kDa	0.0231
435	PREDICTED: glycerol kinase isoform X4	59 kDa	0.0231
436	translocase of outer mitochondrial membrane 20 homolog a	16 kDa	0.023
437	40S ribosomal protein S5	25 kDa	0.023
438	PREDICTED: calnexin isoform X1	67 kDa	0.0229
439	1-acyl-sn-glycerol-3-phosphate acyltransferase epsilon	42 kDa	0.0229
440	PREDICTED: probable palmitoyltransferase ZDHHC14 isoform X1	16 kDa	0.0227
441	PREDICTED: protein CCSMST1	16 kDa	0.0227
442	glutathione peroxidase 1	16 kDa	0.0227
443	PREDICTED: solute carrier family 25 member 51-like	34 kDa	0.0226
444	PREDICTED: kynurenine--oxoglutarate transaminase 1 isoform X1	51 kDa	0.0225
445	PREDICTED: hydroxyacylglutathione hydrolase, mitochondrial isoform X1	34 kDa	0.0225
446	PREDICTED: uncharacterized protein LOC100694464	17 kDa	0.0223
447	mitochondrial import inner membrane translocase subunit TIM44	52 kDa	0.0222
448	bcl-2-modifying factor	17 kDa	0.0221
449	reticulon-1 isoform 1	17 kDa	0.0221
450	PREDICTED: retinol dehydrogenase 12-like	34 kDa	0.0221
451	PREDICTED: cadherin-related family member 5-like isoform X2	44 kDa	0.0217
452	40S ribosomal protein S7	17 kDa	0.0216
453	PREDICTED: coiled-coil-helix-coiled-coil-helix domain-containing protein 2, mitochondrial	17 kDa	0.0213
454	PREDICTED: 60S ribosomal protein L9 isoform X2	17 kDa	0.0213
455	PREDICTED: 40S ribosomal protein S24	17 kDa	0.0212
456	lipoamide acyltransferase component of branched-chain alpha-keto acid dehydrogenase complex, mitochondrial	54 kDa	0.021
457	PREDICTED: AFG3-like protein 1	27 kDa	0.021
458	PREDICTED: 60S ribosomal protein L12	18 kDa	0.0209
459	protein SCO2 homolog, mitochondrial	36 kDa	0.0207
460	isovaleryl-CoA dehydrogenase, mitochondrial	46 kDa	0.0206

461	PREDICTED: transmembrane protein 43-like	37 kDa	0.0205
462	PREDICTED: peptidyl-prolyl cis-trans isomerase FKBP8 isoform X1	37 kDa	0.0201
463	uncharacterized protein LOC100127838	18 kDa	0.0201
464	PREDICTED: 60S ribosomal protein L23a	18 kDa	0.0201
465	PREDICTED: neutral alpha-glucosidase AB	18 kDa	0.0201
466	coiled-coil domain-containing protein 51	47 kDa	0.02
467	PREDICTED: sodium/potassium-transporting ATPase subunit beta-2-like	18 kDa	0.02
468	PREDICTED: [Pyruvate dehydrogenase (acetyl-transferring)] kinase isozyme 1, mitochondrial	47 kDa	0.02
469	flotillin-2a	47 kDa	0.0199
470	PREDICTED: epoxide hydrolase 1, partial	38 kDa	0.0199
471	40S ribosomal protein S16	18 kDa	0.0198
472	dnaJ homolog subfamily C member 2	48 kDa	0.0197
473	solute carrier family 25 member 40	39 kDa	0.0193
474	PREDICTED: alpha-aminoadipic semialdehyde dehydrogenase	59 kDa	0.019
475	40S ribosomal protein S4, X isoform	29 kDa	0.019
476	mitofusin-2	80 kDa	0.0189
477	PREDICTED: fatty aldehyde dehydrogenase isoform X2	40 kDa	0.0188
478	dolichyl-diphosphooligosaccharide--protein glycosyltransferase subunit 2 precursor	70 kDa	0.0187
479	carnitine O-palmitoyltransferase 2, mitochondrial	20 kDa	0.0186
480	ER membrane protein complex subunit 3	30 kDa	0.0185
481	methylmalonic aciduria type A protein, mitochondrial	30 kDa	0.0185
482	peripherin-2	20 kDa	0.0184
483	PREDICTED: surfeit locus protein 4	30 kDa	0.0184
484	60S ribosomal protein L7a	30 kDa	0.0184
485	PREDICTED: uncharacterized protein si:ch211-11k18.4	51 kDa	0.0184
486	PREDICTED: very long-chain specific acyl-CoA dehydrogenase, mitochondrial isoform X1	71 kDa	0.0183
487	hydroxysteroid dehydrogenase-like protein 2	51 kDa	0.0182
488	PREDICTED: paraplegin	92 kDa	0.0182
489	PREDICTED: calcium-binding mitochondrial carrier protein SCaMC-3-like isoform X2	41 kDa	0.0182
490	regulator of microtubule dynamics protein 3	51 kDa	0.018
491	PREDICTED: 40S ribosomal protein S2	20 kDa	0.018
492	NADH dehydrogenase 1 beta subcomplex subunit 10	20 kDa	0.018
493	long-chain fatty acid transport protein 4	73 kDa	0.0179
494	mitochondrial dynamics protein MID49	52 kDa	0.0177
495	PREDICTED: epoxide hydrolase 1-like, partial	20 kDa	0.0176
496	PREDICTED: dehydrogenase/reductase SDR family member 7B isoform X1	31 kDa	0.0176
497	PREDICTED: 3 beta-hydroxysteroid dehydrogenase type 7 isoform X1	42 kDa	0.0176
498	60S ribosomal protein L4	43 kDa	0.0172
499	ras-related protein Rap-1b-like	21 kDa	0.0171
500	ADP-ribosylation factor-like protein 6	21 kDa	0.0171
501	translocon-associated protein subunit delta precursor	21 kDa	0.017
502	tryptophan 5-hydroxylase 1	54 kDa	0.017
503	PREDICTED: bcl10-interacting CARD protein isoform X2	21 kDa	0.0169
504	PREDICTED: cytochrome c oxidase subunit 4 isoform 1, mitochondrial	21 kDa	0.0169
505	uncharacterized protein LOC393228	21 kDa	0.0168
506	PREDICTED: protein FAM57A-like	32 kDa	0.0168
507	PREDICTED: fatty aldehyde dehydrogenase-like	55 kDa	0.0167
508	outer dense fiber of sperm tails 2b	22 kDa	0.0166
509	PRA1 family protein 3	22 kDa	0.0166
510	fumarate hydratase, mitochondrial precursor	55 kDa	0.0166
511	60S ribosomal protein L9	22 kDa	0.0163
512	abhydrolase domain-containing protein 4	22 kDa	0.0163
513	PREDICTED: synembryn-A-like isoform X2	67 kDa	0.0163
514	protein kinase, cAMP-dependent, regulatory, type II, alpha A	45 kDa	0.0163
515	ubiquinone biosynthesis protein COQ7 homolog	22 kDa	0.0162
516	carnitine O-palmitoyltransferase 2, mitochondrial	22 kDa	0.0162
517	eukaryotic translation elongation factor 1 alpha 1-like	22 kDa	0.0161
518	solute carrier family 25 member 46	45 kDa	0.016

519	isobutyryl-CoA dehydrogenase, mitochondrial	45 kDa	0.016
520	protein-S-isoprenylcysteine O-methyltransferase	34 kDa	0.016
521	PREDICTED: probable arginine--tRNA ligase, mitochondrial isoform X1	22 kDa	0.016
522	PREDICTED: 10 kDa heat shock protein, mitochondrial isoform X2	11 kDa	0.0159
523	PREDICTED: aarF domain-containing protein kinase 4	11 kDa	0.0159
524	PREDICTED: saccharopine dehydrogenase-like oxidoreductase	11 kDa	0.0159
525	succinyl-CoA:3-ketoacid coenzyme A transferase 1, mitochondrial	57 kDa	0.0159
526	acyl-CoA dehydrogenase family member 9, mitochondrial	69 kDa	0.0159
527	succinyl-CoA ligase	34 kDa	0.0159
528	dihydroorotate dehydrogenase (quinone), mitochondrial	46 kDa	0.0158
529	aspartate aminotransferase, cytoplasmic	46 kDa	0.0158
530	PREDICTED: 60S ribosomal protein L18a-like	23 kDa	0.0158
531	60S acidic ribosomal protein P0	34 kDa	0.0157
532	mitochondrial ubiquitin ligase activator of nfkb 1-A	23 kDa	0.0156
533	methylglutaconyl-CoA hydratase, mitochondrial	35 kDa	0.0156
534	PREDICTED: transmembrane protein C9orf123 homolog	11 kDa	0.0154
535	saccharopine dehydrogenase b	47 kDa	0.0154
536	PREDICTED: flotillin 1a isoform X1	47 kDa	0.0153
537	PREDICTED: serine protease HTRA2, mitochondrial	47 kDa	0.0153
538	electron transfer flavoprotein subunit alpha, mitochondrial	35 kDa	0.0153
539	ADP-ribosylation factor-like protein 6-interacting protein 1	23 kDa	0.0153
540	PREDICTED: heme oxygenase 2	35 kDa	0.0152
541	transmembrane protein 65	24 kDa	0.015
542	ras-related protein Rab-5B	24 kDa	0.015
543	uncharacterized protein LOC100005854	24 kDa	0.015
544	PREDICTED: required for meiotic nuclear division protein 1 homolog isoform X1	48 kDa	0.0149
545	DnaJ (Hsp40) homolog, subfamily A, member 3B	36 kDa	0.0148
546	protein lunapark-A	36 kDa	0.0147
547	putative hexokinase HKDC1	61 kDa	0.0147
548	delta-1-pyrroline-5-carboxylate dehydrogenase, mitochondrial precursor	62 kDa	0.0146
549	cytosol aminopeptidase	37 kDa	0.0145
550	transmembrane emp24 domain-containing protein 10 precursor	24 kDa	0.0145
551	probable 28S ribosomal protein S10, mitochondrial	12 kDa	0.0145
552	fructose-bisphosphate aldolase C	37 kDa	0.0145
553	ubiquitin-60S ribosomal protein L40	12 kDa	0.0143
554	phosphatidylglycerophosphatase and protein-tyrosine phosphatase 1	12 kDa	0.0143
555	heat shock protein HSP 90-beta	89 kDa	0.0143
556	superoxide dismutase	25 kDa	0.0141
557	peroxisomal membrane protein 11B	25 kDa	0.0141
558	selenoprotein T1a precursor	12 kDa	0.0141
559	PREDICTED: glutaminase kidney isoform, mitochondrial isoform X2	38 kDa	0.014
560	dynamin-1-like protein	77 kDa	0.0139
561	phosphatidate cytidylyltransferase, mitochondrial precursor	12 kDa	0.0139
562	PRELI domain containing 1b	25 kDa	0.0139
563	UBX domain-containing protein 4	52 kDa	0.0138
564	cytochrome P450, family 20, subfamily A, polypeptide 1	52 kDa	0.0138
565	transmembrane protein 147	25 kDa	0.0138
566	NAD-dependent malic enzyme, mitochondrial	65 kDa	0.0138
567	PREDICTED: 40S ribosomal protein S2	12 kDa	0.0136
568	PREDICTED: neural cell adhesion molecule 1b isoform X11	92 kDa	0.0136
569	Probable saccharopine dehydrogenase	26 kDa	0.0135
570	PREDICTED: UPF0562 protein C7orf55 homolog	13 kDa	0.0135
571	probable asparagine--tRNA ligase, mitochondrial	26 kDa	0.0135
572	oxoglutarate (alpha-ketoglutarate) dehydrogenase (lipoamide)	26 kDa	0.0134
573	pyruvate dehydrogenase E1 component subunit alpha, somatic form, mitochondrial	13 kDa	0.0134
574	diablo, IAP-binding mitochondrial protein a	13 kDa	0.0133
575	PREDICTED: phospholipid scramblase 2	26 kDa	0.0132
576	BRI3-binding protein precursor	26 kDa	0.0132
577	rod outer segment membrane protein 1	40 kDa	0.0132

578	uncharacterized protein LOC100124614	13 kDa	0.0131
579	39S ribosomal protein L17, mitochondrial	13 kDa	0.013
580	flotillin-1	41 kDa	0.013
581	mannose-P-dolichol utilization defect 1 protein	27 kDa	0.0128
582	nicastrin precursor	42 kDa	0.0127
583	PREDICTED: transmembrane protein 186 isoform X1	13 kDa	0.0127
584	carnitine O-acetyltransferase b	70 kDa	0.0126
585	PREDICTED: acyl-CoA-binding domain-containing protein 5A isoform X2	56 kDa	0.0126
586	PREDICTED: proteoglycan 4-like isoform X1	13 kDa	0.0126
587	syntaxin binding protein 1b	57 kDa	0.0124
588	60S ribosomal protein L8	28 kDa	0.0124
589	PREDICTED: 40S ribosomal protein S3-like isoform X1	28 kDa	0.0122
590	solute carrier family 25 member 36-A	28 kDa	0.0122
591	aquaporin-9	29 kDa	0.0122
592	LYR motif containing protein 1	14 kDa	0.0121
593	60S ribosomal protein L30	14 kDa	0.012
594	acyl-coenzyme A thioesterase THEM4	29 kDa	0.012
595	PREDICTED: 40S ribosomal protein S15	14 kDa	0.0119
596	prenylcysteine oxidase 1 precursor	59 kDa	0.0118
597	ES1 protein homolog, mitochondrial	29 kDa	0.0118
598	dehydrogenase/reductase SDR family member 4	29 kDa	0.0117
599	PREDICTED: lymphocyte antigen 6D-like isoform X1	14 kDa	0.0117
600	PREDICTED: AFG3-like protein 1	60 kDa	0.0117
601	PREDICTED: pyruvate kinase PKM isoform X1	60 kDa	0.0117
602	PREDICTED: uncharacterized aarF domain-containing protein kinase 2	14 kDa	0.0116
603	PREDICTED: protein HEG homolog 1 isoform X3	45 kDa	0.0116
604	PREDICTED: potassium voltage-gated channel subfamily V member 2	60 kDa	0.0116
605	ubiquitin-conjugating enzyme E2 J1	30 kDa	0.0115
606	alpha-1,3/1,6-mannosyltransferase ALG2	46 kDa	0.0115
607	PREDICTED: ADP-ribosylation factor-like protein 13A isoform X1	46 kDa	0.0115
608	complement component 1 Q subcomponent-binding protein, mitochondrial	30 kDa	0.0115
609	PREDICTED: ATP synthase-coupling factor 6, mitochondrial isoform X1	15 kDa	0.0115
610	uncharacterized protein LOC447917 precursor	30 kDa	0.0114
611	F-box/LRR-repeat protein 2	46 kDa	0.0114
612	membrane magnesium transporter 1 precursor	15 kDa	0.0114
613	PREDICTED: pre-B-cell leukemia transcription factor-interacting protein 1 isoform X3	15 kDa	0.0114
614	PREDICTED: 40S ribosomal protein S15a isoform X1	15 kDa	0.0113
615	40S ribosomal protein S6	31 kDa	0.0112
616	isoleucine--tRNA ligase, mitochondrial	110 kDa	0.0111
617	propionyl-CoA carboxylase alpha chain, mitochondrial	79 kDa	0.0111
618	PREDICTED: cGMP-gated cation channel alpha-1	63 kDa	0.011
619	Meckel syndrome type 1 protein	15 kDa	0.011
620	ancient ubiquitous protein 1	48 kDa	0.0109
621	transcription factor A, mitochondrial	31 kDa	0.0109
622	holocytochrome c synthetase a	32 kDa	0.0109
623	HIG1 domain family member 2A, mitochondrial	15 kDa	0.0108
624	iron-sulfur cluster assembly 1 homolog, mitochondrial precursor	15 kDa	0.0108
625	PREDICTED: phosphorylase, glycogen, muscle b isoform X1	97 kDa	0.0108
626	PREDICTED: sphingomyelin phosphodiesterase 2 isoform X1	48 kDa	0.0108
627	PREDICTED: LOW QUALITY PROTEIN: metalloendopeptidase OMA1, mitochondrial	16 kDa	0.0108
628	60S ribosomal protein L31	16 kDa	0.0108
629	[3-methyl-2-oxobutanoate dehydrogenase	48 kDa	0.0107
630	N-acetyltransferase 14	32 kDa	0.0107
631	PREDICTED: ethylmalonyl-CoA decarboxylase isoform X1	32 kDa	0.0107
632	protein FAM136A	16 kDa	0.0107
633	solute carrier family 25 member 35	32 kDa	0.0106
634	PREDICTED: serine/threonine-protein phosphatase 2A 65 kDa regulatory subunit A beta isoform isoform X1	65 kDa	0.0106
635	PREDICTED: translocon-associated protein subunit alpha-like isoform X3	32 kDa	0.0105

636	PREDICTED: RNA-binding protein Musashi homolog 1-like isoform X1	33 kDa	0.0105
637	NADH dehydrogenase	16 kDa	0.0105
638	PREDICTED: sarcoplasmic/endoplasmic reticulum calcium ATPase 2	116 kDa	0.0105
639	rod cGMP-specific 3',5'-cyclic phosphodiesterase subunit alpha	99 kDa	0.0105
640	PREDICTED: glycerol-3-phosphate dehydrogenase, mitochondrial isoform X2	66 kDa	0.0105
641	uncharacterized protein LOC560648	16 kDa	0.0104
642	PREDICTED: protein CCSMST1	16 kDa	0.0104
643	RPE-retinal G protein-coupled receptor	33 kDa	0.0103
644	PREDICTED: enoyl-CoA delta isomerase 1, mitochondrial	33 kDa	0.0102
645	epidermal retinol dehydrogenase 2	34 kDa	0.0101
646	PREDICTED: vesicle-fusing ATPase isoform X1	86 kDa	0.01
647	PREDICTED: pre-B-cell leukemia transcription factor-interacting protein 1 isoform X3	17 kDa	0.01
648	solute carrier family 25 member 33	34 kDa	0.0099
649	solute carrier family 25 member 33	34 kDa	0.0099
650	mitochondrial dynamics protein MID51	52 kDa	0.0099
651	PREDICTED: protein transport protein Sec61 subunit alpha-like 1	52 kDa	0.0099
652	FUN14 domain-containing protein 1	17 kDa	0.0098
653	uncharacterized protein LOC100135257	35 kDa	0.0097
654	elongation of very long chain fatty acids-like 4	35 kDa	0.0097
655	uncharacterized protein C15orf61 homolog	17 kDa	0.0097
656	CAAX prenyl protease 1 homolog	53 kDa	0.0096
657	PREDICTED: cadherin-related family member 5-like isoform X2	17 kDa	0.0096
658	PREDICTED: 40S ribosomal protein S23-like	17 kDa	0.0096
659	PREDICTED: LETM1 domain-containing protein LETM2, mitochondrial	54 kDa	0.0096
660	PREDICTED: mitochondrial 10-formyltetrahydrofolate dehydrogenase	90 kDa	0.0096
661	PREDICTED: 60S ribosomal protein L23	17 kDa	0.0095
662	28S ribosomal protein S14, mitochondrial	17 kDa	0.0095
663	peptidyl-prolyl cis-trans isomerase A	17 kDa	0.0095
664	thioredoxin-related transmembrane protein 2-B precursor	36 kDa	0.0095
665	putative Ras-related protein Rab-42	18 kDa	0.0095
666	carnitine O-palmitoyltransferase 2, mitochondrial	18 kDa	0.0094
667	endoplasmin precursor	92 kDa	0.0094
668	glycerophosphodiester phosphodiesterase 1	36 kDa	0.0094
669	PREDICTED: poliovirus receptor-related protein 1-like isoform X2	55 kDa	0.0094
670	PREDICTED: acyl carrier protein, mitochondrial	18 kDa	0.0093
671	uncharacterized protein LOC100135288	18 kDa	0.0093
672	PREDICTED: mitochondrial coenzyme A transporter SLC25A42 isoform X1	36 kDa	0.0093
673	39S ribosomal protein L20, mitochondrial	18 kDa	0.0093
674	PREDICTED: glycerol-3-phosphate acyltransferase 1, mitochondrial	93 kDa	0.0092
675	presequence protease, mitochondrial precursor	18 kDa	0.0092
676	limbic system-associated membrane protein precursor	37 kDa	0.0092
677	calcium/calmodulin-dependent protein kinase type II delta 1 chain isoform 1	56 kDa	0.0092
678	arginase-1	37 kDa	0.0092
679	coiled-coil domain-containing protein 47 precursor	56 kDa	0.0092
680	dehydrogenase/reductase SDR family member 7	37 kDa	0.0091
681	PREDICTED: 60S ribosomal protein L27-like	18 kDa	0.0091
682	succinate dehydrogenase	18 kDa	0.0091
683	PREDICTED: protein GPR107 isoform X1	56 kDa	0.0091
684	40S ribosomal protein S18	18 kDa	0.009
685	inositol monophosphatase 3	37 kDa	0.009
686	PREDICTED: 2-oxoglutarate dehydrogenase-like, mitochondrial	115 kDa	0.009
687	peripherin-2	18 kDa	0.009
688	PREDICTED: T-cell activation inhibitor, mitochondrial	57 kDa	0.009
689	NADH dehydrogenase (ubiquinone) 1, alpha/beta subcomplex 1-like	19 kDa	0.0089
690	PREDICTED: 60S ribosomal protein L21	19 kDa	0.0089
691	PREDICTED: protein ERGIC-53	57 kDa	0.0089
692	PREDICTED: lysine (K)-specific demethylase 6A, like isoform X1	19 kDa	0.0089
693	PREDICTED: translocation protein SEC62	19 kDa	0.0089
694	amine oxidase	59 kDa	0.0087

695	PREDICTED: lysophosphatidylcholine acyltransferase 1-like	59 kDa	0.0087
696	PREDICTED: rod cGMP-specific 3',5'-cyclic phosphodiesterase subunit beta	99 kDa	0.0087
697	cytosolic 5'-nucleotidase 3	39 kDa	0.0086
698	D-beta-hydroxybutyrate dehydrogenase, mitochondrial	40 kDa	0.0085
699	PREDICTED: choline dehydrogenase, mitochondrial	20 kDa	0.0085
700	polyribonucleotide nucleotidyltransferase 1, mitochondrial	60 kDa	0.0084
701	ATPase family AAA domain-containing protein 1-B	20 kDa	0.0084
702	peripherin-2	20 kDa	0.0084
703	mitochondrial ubiquitin ligase activator of NFKB 1	40 kDa	0.0083
704	PREDICTED: probable hydrolase PNKD	20 kDa	0.0083
705	renalase	41 kDa	0.0082
706	PREDICTED: clathrin coat assembly protein AP180 isoform X5	41 kDa	0.0082
707	PREDICTED: protein tyrosine phosphatase type IVA 3-like isoform X3	20 kDa	0.0082
708	PREDICTED: cytochrome c oxidase assembly factor 1 homolog isoform X3	20 kDa	0.0082
709	PREDICTED: coenzyme Q-binding protein COQ10 homolog A, mitochondrial	20 kDa	0.0082
710	alpha-aminoadipic semialdehyde synthase, mitochondrial	105 kDa	0.0081
711	L-threonine 3-dehydrogenase, mitochondrial	42 kDa	0.0081
712	40S ribosomal protein S10	20 kDa	0.0081
713	PREDICTED: cGMP-gated cation channel alpha-1	63 kDa	0.008
714	LIM domain and actin-binding protein 1	20 kDa	0.008
715	PREDICTED: signal peptidase complex catalytic subunit SEC11A-like	21 kDa	0.008
716	PREDICTED: heparan sulfate 2-O-sulfotransferase 1	42 kDa	0.008
717	3-hydroxyisobutyryl-CoA hydrolase, mitochondrial	42 kDa	0.008
718	coatomer subunit gamma-2	42 kDa	0.008
719	PREDICTED: protein TsetseEP-like	21 kDa	0.008
720	PREDICTED: ADP-ribosylation factor-like protein 13B-like isoform X5	42 kDa	0.008
721	enoyl-CoA delta isomerase 2, mitochondrial	42 kDa	0.0079
722	PREDICTED: renin receptor isoform X1	42 kDa	0.0079
723	probable 2-oxoglutarate dehydrogenase E1 component DHKTD1, mitochondrial	107 kDa	0.0079
724	cytosol aminopeptidase	21 kDa	0.0078
725	PREDICTED: translocon-associated protein subunit gamma	21 kDa	0.0078
726	39S ribosomal protein L12, mitochondrial	21 kDa	0.0078
727	PREDICTED: cell division control protein 42 homolog isoform X1	21 kDa	0.0077
728	PREDICTED: transmembrane and coiled-coil domains protein 1-like	21 kDa	0.0077
729	PREDICTED: meckelin isoform X2	111 kDa	0.0077
730	Bcl-2/adenovirus E1B 19kD interaction protein XR	22 kDa	0.0076
731	basigin precursor	45 kDa	0.0075
732	phosphoglycerate kinase 1	45 kDa	0.0075
733	neuroplastin precursor	45 kDa	0.0075
734	protein FAM134A	45 kDa	0.0075
735	PREDICTED: protein RER1 isoform X2	22 kDa	0.0075
736	PREDICTED: lipoamide acyltransferase component of branched-chain alpha-keto acid dehydrogenase complex, mitochondrial-like	45 kDa	0.0075
737	1-acyl-sn-glycerol-3-phosphate acyltransferase gamma	45 kDa	0.0074
738	PREDICTED: glucose-induced degradation protein 8 homolog	22 kDa	0.0074
739	tyrosine-protein phosphatase non-receptor type 1	22 kDa	0.0074
740	PREDICTED: GTP-binding protein SAR1b	22 kDa	0.0074
741	28S ribosomal protein S27, mitochondrial	46 kDa	0.0073
742	presequence protease, mitochondrial precursor	115 kDa	0.0073
743	PREDICTED: cytochrome b-c1 complex subunit 6, mitochondrial-like	23 kDa	0.0073
744	2-amino-3-ketobutyrate coenzyme A ligase, mitochondrial	46 kDa	0.0072
745	ADP-ribosylation factor-like 15a	23 kDa	0.0072
746	transmembrane protein 160	23 kDa	0.0072
747	PREDICTED: probable peptidyl-tRNA hydrolase isoform X1	23 kDa	0.0072
748	mpv17-like protein 2	23 kDa	0.0072
749	electron transfer flavoprotein-ubiquinone oxidoreductase, mitochondrial	23 kDa	0.0072
750	PREDICTED: short/branched chain specific acyl-CoA dehydrogenase, mitochondrial	47 kDa	0.0071
751	uncharacterized protein LOC492355	23 kDa	0.0071
752	eukaryotic initiation factor 4A-II	47 kDa	0.0071
753	isocitrate dehydrogenase	47 kDa	0.0071

754	ras-related protein Rab-18-B	23 kDa	0.0071
755	PREDICTED: peptidyl-prolyl cis-trans isomerase A-like	23 kDa	0.0071
756	PREDICTED: ras-related protein Rab-7a isoform X1	24 kDa	0.007
757	recoverin	23 kDa	0.007
758	serum/glucocorticoid regulated kinase 1-like	48 kDa	0.0069
759	mitochondrial import inner membrane translocase subunit Tim10 B	24 kDa	0.0069
760	keratin, type I cytoskeletal 18	49 kDa	0.0068
761	PREDICTED: neural cell adhesion molecule 1 isoform X1	74 kDa	0.0068
762	nucleoside diphosphate kinase 7	24 kDa	0.0068
763	ELMO domain-containing protein 2 precursor	24 kDa	0.0067
764	PREDICTED: sialic acid-binding Ig-like lectin 6	24 kDa	0.0067
765	protein FAM173A	24 kDa	0.0067
766	PREDICTED: 60S ribosomal protein L13	24 kDa	0.0067
767	PREDICTED: glucose-6-phosphate translocase	50 kDa	0.0067
768	PREDICTED: amyloid-like protein 2 isoform X2	24 kDa	0.0067
769	60S ribosomal protein L10	25 kDa	0.0067
770	maleylacetoacetate isomerase isoform 2	25 kDa	0.0067
771	PREDICTED: GRAM domain-containing protein 2-like isoform X3	25 kDa	0.0066
772	prolactin regulatory element-binding protein	25 kDa	0.0066
773	hypoxanthine-guanine phosphoribosyltransferase	25 kDa	0.0066
774	60S ribosomal protein L10a	25 kDa	0.0066
775	UMP-CMP kinase	25 kDa	0.0066
776	GTP:AMP phosphotransferase AK3, mitochondrial	25 kDa	0.0065
777	phosphatidylinositide phosphatase SAC1-A	51 kDa	0.0065
778	solute carrier family 25 member 47-A	25 kDa	0.0065
779	28S ribosomal protein S34, mitochondrial	25 kDa	0.0065
780	PREDICTED: V-type proton ATPase subunit S1	52 kDa	0.0064
781	PREDICTED: phosphatidate cytidylyltransferase 1	52 kDa	0.0064
782	PREDICTED: glutaminase kidney isoform, mitochondrial isoform X3	26 kDa	0.0064
783	bcl-2-like protein 13	52 kDa	0.0063
784	N-acylneuraminate cytidylyltransferase	53 kDa	0.0063
785	polyribonucleotide nucleotidyltransferase 1, mitochondrial	26 kDa	0.0063
786	probable leucine--tRNA ligase, mitochondrial	26 kDa	0.0063
787	PREDICTED: dnaJ homolog subfamily C member 30-like isoform X1	26 kDa	0.0062
788	peroxisomal biogenesis factor 3	26 kDa	0.0062
789	PREDICTED: lon protease homolog, mitochondrial	108 kDa	0.0062
790	insulin receptor a precursor	27 kDa	0.0061
791	mitochondrial peptide methionine sulfoxide reductase	27 kDa	0.0061
792	fascin-2	55 kDa	0.0061
793	phosducin	27 kDa	0.006
794	diablo homolog, mitochondrial	27 kDa	0.006
795	PREDICTED: putative Dol-P-Glc:Glc(2)Man(9)GlcNAc(2)-PP-Dol alpha-1,2-glucosyltransferase	55 kDa	0.006
796	triosephosphate isomerase A	27 kDa	0.006
797	PREDICTED: glutathione reductase, mitochondrial isoform X2	27 kDa	0.006
798	methylmalonyl-CoA mutase, mitochondrial	84 kDa	0.006
799	diablo homolog, mitochondrial	27 kDa	0.0059
800	HCLS1-associated protein X-1	28 kDa	0.0059
801	dol-P-Man:Man(7)GlcNAc(2)-PP-Dol alpha-1,6-mannosyltransferase precursor	56 kDa	0.0059
802	aldehyde dehydrogenase 2 family (mitochondrial), tandem duplicate 2	57 kDa	0.0058
803	dolichol-phosphate mannosyltransferase subunit 1	28 kDa	0.0058
804	ER membrane protein complex subunit 10 isoform 1 precursor	28 kDa	0.0058
805	neural cell adhesion molecule 1 precursor	28 kDa	0.0058
806	PREDICTED: Golgi SNAP receptor complex member 1 isoform X1	28 kDa	0.0057
807	PREDICTED: solute carrier family 1 (glial high affinity glutamate transporter), member 2a isoform X2	58 kDa	0.0057
808	transmembrane protein 33	29 kDa	0.0057
809	PREDICTED: cell adhesion molecule 3 isoform X1	58 kDa	0.0056
810	ATP-dependent Clp protease proteolytic subunit, mitochondrial	29 kDa	0.0056
811	PREDICTED: transmembrane protein 101	29 kDa	0.0056

812	28S ribosomal protein S2, mitochondrial	29 kDa	0.0055
813	glutamate dehydrogenase 1b	60 kDa	0.0055
814	dol-P-Man:Man(5)GlcNAc(2)-PP-Dol alpha-1,3-mannosyltransferase	30 kDa	0.0055
815	alpha/beta hydrolase domain-containing protein 14A	30 kDa	0.0055
816	PREDICTED: protein phosphatase 1 regulatory subunit 16A	61 kDa	0.0054
817	PREDICTED: ATPase, Ca++ transporting, cardiac muscle, slow twitch 2b isoform X1	92 kDa	0.0054
818	actin-like protein 6A	31 kDa	0.0053
819	PREDICTED: SEC14-like protein 3 isoform X1	31 kDa	0.0052
820	L-2-hydroxyglutarate dehydrogenase, mitochondrial	31 kDa	0.0052
821	T-complex protein 1 subunit gamma	63 kDa	0.0052
822	PREDICTED: G-protein coupled receptor 98 isoform X2	31 kDa	0.0052
823	leucine-rich repeat, immunoglobulin-like and transmembrane domains 1 precursor	64 kDa	0.0051
824	leucine-rich PPR motif-containing protein, mitochondrial	162 kDa	0.0051
825	B-cell receptor-associated protein 31	32 kDa	0.0051
826	thioredoxin-related transmembrane protein 1 precursor	32 kDa	0.0051
827	uncharacterized protein C18orf19 homolog B	32 kDa	0.005
828	PREDICTED: ATP-dependent (S)-NAD(P)H-hydrate dehydratase isoform X10	32 kDa	0.005
829	uncharacterized protein LOC100127834	33 kDa	0.005
830	3-hydroxyisobutyrate dehydrogenase, mitochondrial	33 kDa	0.005
831	40S ribosomal protein S3a	33 kDa	0.005
832	PREDICTED: LOW QUALITY PROTEIN: metalloendopeptidase OMA1, mitochondrial	33 kDa	0.0049
833	3-mercaptopyruvate sulfurtransferase	33 kDa	0.0049
834	PREDICTED: protein FAM134C	33 kDa	0.0049
835	carnitine O-acetyltransferase	68 kDa	0.0048
836	PREDICTED: protein CLN8 isoform X1	34 kDa	0.0048
837	PREDICTED: carnitine palmitoyltransferase 1A isoform X2	69 kDa	0.0048
838	PREDICTED: uncharacterized protein KIAA1467 homolog isoform X1	69 kDa	0.0048
839	40S ribosomal protein SA	34 kDa	0.0048
840	malonyl-CoA decarboxylase, mitochondrial	34 kDa	0.0048
841	solute carrier family 7, member 3	70 kDa	0.0047
842	PREDICTED: transmembrane and TPR repeat-containing protein 3	105 kDa	0.0047
843	enoyl-CoA hydratase domain-containing protein 2, mitochondrial	35 kDa	0.0047
844	dehydrogenase/reductase (SDR family) member 13a, duplicate 3	35 kDa	0.0047
845	PREDICTED: mitochondrial thiamine pyrophosphate carrier isoform X1	35 kDa	0.0047
846	heme oxygenase 2	35 kDa	0.0047
847	glyoxylate reductase/hydroxypyruvate reductase	35 kDa	0.0046
848	PREDICTED: mitochondrial Rho GTPase 1 isoform X4	71 kDa	0.0046
849	PREDICTED: uncharacterized protein LOC100126019 isoform X1	107 kDa	0.0046
850	PREDICTED: CAAX prenyl protease 2	35 kDa	0.0046
851	long-chain fatty acid transport protein 1	72 kDa	0.0046
852	PREDICTED: zinc finger protein-like 1 isoform X1	36 kDa	0.0045
853	methylcrotonoyl-CoA carboxylase subunit alpha, mitochondrial	72 kDa	0.0045
854	PREDICTED: syntaxin-18 isoform X1	36 kDa	0.0045
855	PREDICTED: coiled-coil domain containing 127a isoform X1	36 kDa	0.0045
856	PREDICTED: trans-Golgi network integral membrane protein 2	36 kDa	0.0045
857	PREDICTED: xyloside xylosyltransferase 1 isoform X1	37 kDa	0.0044
858	uncharacterized protein LOC100170509	75 kDa	0.0043
859	protein phosphatase 1, catalytic subunit, alpha	37 kDa	0.0043
860	PREDICTED: syntaxin-16 isoform X1	38 kDa	0.0043
861	amine oxidase	38 kDa	0.0043
862	neurotrimin isoform 1 precursor	38 kDa	0.0042
863	2-methoxy-6-polyprenyl-1,4-benzoquinol methylase, mitochondrial precursor	38 kDa	0.0042
864	PREDICTED: serine/threonine-protein phosphatase PP1-beta catalytic subunit	38 kDa	0.0042
865	PREDICTED: hydroxyindole O-methyltransferase isoform X1	38 kDa	0.0042
866	enkurin domain-containing protein 1	39 kDa	0.0042
867	PREDICTED: tectonic-2	39 kDa	0.0042
868	cell adhesion molecule 1b precursor	39 kDa	0.0042
869	PREDICTED: ATP-dependent 6-phosphofructokinase, liver type-like	39 kDa	0.0041
870	Bardet-Biedl syndrome 5 protein homolog	39 kDa	0.0041

871	eukaryotic translation initiation factor 4A, isoform 1A	39 kDa	0.0041
872	protein phosphatase 1 regulatory subunit 7 isoform 1	40 kDa	0.0041
873	PREDICTED: ATP-dependent zinc metalloprotease YME1L1-like isoform X1	81 kDa	0.004
874	28S ribosomal protein S22, mitochondrial	40 kDa	0.004
875	PREDICTED: tectonic-2	40 kDa	0.004
876	PREDICTED: red-sensitive opsin-1 isoform X1	41 kDa	0.004
877	cAMP-dependent protein kinase catalytic subunit beta	41 kDa	0.004
878	G-protein coupled receptor 98 precursor	82 kDa	0.0039
879	PREDICTED: protein phosphatase 1K, mitochondrial isoform X1	41 kDa	0.0039
880	PREDICTED: transmembrane protein 237A	42 kDa	0.0039
881	PREDICTED: coiled-coil domain-containing protein 136 isoform X2	42 kDa	0.0038
882	PREDICTED: glycine--tRNA ligase	86 kDa	0.0038
883	PREDICTED: peroxisomal membrane protein PEX14	43 kDa	0.0038
884	polymerase delta-interacting protein 2	43 kDa	0.0037
885	3-ketoacyl-CoA thiolase, mitochondrial	43 kDa	0.0037
886	acyl-CoA:lysophosphatidylglycerol acyltransferase 1	43 kDa	0.0037
887	reticulon-1 isoform 1	88 kDa	0.0037
888	monoacylglycerol lipase ABHD12	44 kDa	0.0037
889	PREDICTED: sorting nexin-13 isoform X3	44 kDa	0.0037
890	ATPase family AAA domain-containing protein 1-A isoform 1	44 kDa	0.0037
891	GPI-anchor transamidase precursor	45 kDa	0.0036
892	28S ribosomal protein S5, mitochondrial precursor	45 kDa	0.0036
893	PREDICTED: POC1 centriolar protein homolog A-like	45 kDa	0.0036
894	PREDICTED: dolichyl-diphosphooligosaccharide--protein glycosyltransferase subunit STT3B	91 kDa	0.0036
895	PREDICTED: centrosomal protein POC5 isoform X1	45 kDa	0.0035
896	28S ribosomal protein S9, mitochondrial	46 kDa	0.0035
897	28S ribosomal protein S29, mitochondrial	46 kDa	0.0035
898	casein kinase 2, alpha 1 polypeptide	46 kDa	0.0035
899	PREDICTED: peptide chain release factor 1, mitochondrial-like	46 kDa	0.0035
900	PREDICTED: dyslexia-associated protein KIAA0319-like protein homolog isoform X1	93 kDa	0.0035
901	medium-chain specific acyl-CoA dehydrogenase, mitochondrial	46 kDa	0.0035
902	PREDICTED: V-type proton ATPase 116 kDa subunit a isoform 2 isoform X2	47 kDa	0.0034
903	PREDICTED: outer dense fiber protein 2	96 kDa	0.0034
904	PREDICTED: neutral alpha-glucosidase AB	96 kDa	0.0034
905	uncharacterized protein C6orf136 homolog	48 kDa	0.0033
906	glycogen synthase kinase-3 alpha	48 kDa	0.0033
907	PREDICTED: mannosyl-oligosaccharide 1,2-alpha-mannosidase IA isoform X1	48 kDa	0.0033
908	PREDICTED: mannosyl-oligosaccharide glucosidase isoform X1	49 kDa	0.0033
909	adenylyl cyclase-associated protein 1	50 kDa	0.0032
910	PREDICTED: NAD kinase 2, mitochondrial isoform X2	50 kDa	0.0032
911	PREDICTED: oxysterol-binding protein-related protein 1-like	50 kDa	0.0032
912	endoplasmic reticulum resident protein 44	50 kDa	0.0032
913	puromycin-sensitive aminopeptidase	102 kDa	0.0032
914	elongation factor 1-gamma	51 kDa	0.0031
915	evolutionarily conserved signaling intermediate in Toll pathway, mitochondrial	52 kDa	0.0031
916	epoxide hydrolase 1	52 kDa	0.0031
917	PREDICTED: monocarboxylate transporter 2	52 kDa	0.0031
918	squalene synthase	53 kDa	0.003
919	PREDICTED: sodium-coupled neutral amino acid transporter 3	53 kDa	0.003
920	PREDICTED: protein LYRIC isoform X1	53 kDa	0.003
921	PREDICTED: AP-2 complex subunit alpha-1 isoform X1	109 kDa	0.003
922	progressive ankylosis-like protein	55 kDa	0.0029
923	V-type proton ATPase subunit H isoform 1	55 kDa	0.0029
924	PREDICTED: succinate-semialdehyde dehydrogenase, mitochondrial isoform X1	55 kDa	0.0029
925	PREDICTED: tudor and KH domain-containing protein isoform X1	56 kDa	0.0029
926	4-aminobutyrate aminotransferase, mitochondrial	56 kDa	0.0029
927	V-type proton ATPase subunit B, brain isoform	56 kDa	0.0028
928	GDP-Man:Man(3)GlcNAc(2)-PP-Dol alpha-1,2-mannosyltransferase isoform 1	57 kDa	0.0028
929	PREDICTED: atlastin-3 isoform X1	58 kDa	0.0028

930	E3 ubiquitin–protein ligase synoviolin precursor	59 kDa	0.0027
931	fatty–acid amide hydrolase 2–A	59 kDa	0.0027
932	PREDICTED: UPF0606 protein KIAA1549 isoform X2	120 kDa	0.0027
933	lysophosphatidylcholine acyltransferase 2	60 kDa	0.0027
934	keratin, type II cytoskeletal 8	61 kDa	0.0026
935	PREDICTED: propionyl–CoA carboxylase beta chain, mitochondrial isoform X1	61 kDa	0.0026
936	PREDICTED: GPI transamidase component PIG–S isoform X2	63 kDa	0.0025
937	PREDICTED: asparagine––tRNA ligase, cytoplasmic	64 kDa	0.0025
938	phenylalanine––tRNA ligase beta subunit	66 kDa	0.0024
939	phosphatidylinositide phosphatase SAC1–B	67 kDa	0.0024
940	PREDICTED: mitochondrial intermediate peptidase	68 kDa	0.0023
941	PREDICTED: V–type proton ATPase catalytic subunit A	68 kDa	0.0023
942	electron transfer flavoprotein–ubiquinone oxidoreductase, mitochondrial	69 kDa	0.0023
943	PREDICTED: aarF domain–containing protein kinase 4	70 kDa	0.0023
944	PREDICTED: long–chain fatty acid transport protein 6–like	70 kDa	0.0023
945	PREDICTED: epidermal growth factor receptor kinase substrate 8 isoform X1	71 kDa	0.0023
946	polyadenylate–binding protein 1	71 kDa	0.0022
947	PREDICTED: signal recognition particle receptor subunit alpha	71 kDa	0.0022
948	cleft lip and palate transmembrane protein 1 homolog	72 kDa	0.0022
949	selenoprotein O	77 kDa	0.0021
950	PREDICTED: striatin–3 isoform X1	79 kDa	0.002
951	PREDICTED: synembryn–A–like	79 kDa	0.002
952	dolichyl–diphosphooligosaccharide––protein glycosyltransferase subunit STT3A	81 kDa	0.002
953	PREDICTED: ribosomal protein S6 kinase alpha–3 isoform X1	83 kDa	0.0019
954	probable C–mannosyltransferase DPY19L1	83 kDa	0.0019
955	ATP–dependent RNA helicase SUPV3L1, mitochondrial precursor	86 kDa	0.0019
956	PREDICTED: heat shock 70 kDa protein 4L	93 kDa	0.0017
957	PREDICTED: V–type proton ATPase 116 kDa subunit a isoform 1 isoform X1	96 kDa	0.0017
958	PREDICTED: oxysterol–binding protein–related protein 8–like	98 kDa	0.0016
959	cone cGMP–specific 3',5'–cyclic phosphodiesterase subunit alpha'	98 kDa	0.0016
960	PREDICTED: cadherin–related family member 1–like isoform X2	99 kDa	0.0016
961	PREDICTED: pyruvate dehydrogenase phosphatase regulatory subunit, mitochondrial–like	99 kDa	0.0016
962	PREDICTED: bifunctional heparan sulfate N–deacetylase/N–sulfotransferase 1	102 kDa	0.0016
963	PREDICTED: potassium/sodium hyperpolarization–activated cyclic nucleotide–gated channel 1	102 kDa	0.0016
964	PREDICTED: kinesin–like protein KIFC3 isoform X5	110 kDa	0.0014
965	PREDICTED: spermatogenesis–associated protein 20	115 kDa	0.0014
966	von Willebrand factor A domain–containing protein 8	126 kDa	0.0013
967	PREDICTED: nodal modulator 1	130 kDa	0.0012
968	PREDICTED: PDZ domain–containing protein 8	131 kDa	0.0012
969	PREDICTED: 26S proteasome non–ATPase regulatory subunit 1–like	148 kDa	0.0011
970	PREDICTED: protein fantom isoform X1	215 kDa	0.0007
971	PREDICTED: rootletin isoform X1	226 kDa	0.0007
