# Supplementary material for: Purification of cone outer segment for proteomic analysis on its membrane proteins in carp retina
Source: PLoS One. 2017 Mar 14;12(3):e0173908. doi: 10.1371/journal.pone.0173908 (PMC5349680; doi:10.1371/journal.pone.0173908)
Supplement: S3 Table — Proteins in washed COS-rich fraction were identified with LC-MS/MS analysis and are listed in descending order of emPAI values for 5 × 10^5^ cones. (PDF) [file pone.0173908.s003.pdf]

S3 Table. Identified proteins in washed COS-rich fraction. Proteins in washed COS-rich fraction were identified with LC-MS/MS analysis and are listed in descending order of emPAI values for 5×10^5 cones.

	Identified proteins in washed COS-rich fraction	Molecular mass	emPAI
1	uncharacterized protein LOC100145214	33 kDa	277.27
2	ba1 globin, like	16 kDa	36.229
3	guanine nucleotide-binding protein G(t) subunit alpha-2	40 kDa	28.315
4	voltage-dependent anion-selective channel protein 1	31 kDa	22.103
5	mitochondrial 2-oxoglutarate/malate carrier protein	38 kDa	19.592
6	ATP synthase F(0) complex subunit B1, mitochondrial	31 kDa	14.092
7	voltage-dependent anion-selective channel protein 2	30 kDa	13.789
8	PREDICTED: ATP synthase subunit alpha, mitochondrial	27 kDa	10.735
9	voltage-dependent anion-selective channel protein 2-like	30 kDa	9.6742
10	arrestin-C	40 kDa	8.5749
11	PREDICTED: voltage-dependent anion-selective channel protein 2	11 kDa	7.348
12	PREDICTED: prohibitin isoform X2	22 kDa	7.3326
13	PREDICTED: prohibitin	22 kDa	7.2457
14	ADP/ATP translocase 3	27 kDa	7.2237
15	PREDICTED: solute carrier family 25 (mitochondrial carrier; phosphate carrier), member 3a isoform X2	40 kDa	6.9013
16	alpha globin-like	15 kDa	6.5356
17	PREDICTED: regulator of G-protein signaling 9-binding protein-like	27 kDa	6.3251
18	NADH dehydrogenase 1 beta subcomplex subunit 6	15 kDa	5.3325
19	ATP synthase subunit g, mitochondrial	11 kDa	5.1649
20	band 3 anion transport protein	11 kDa	5.0632
21	ATP synthase subunit alpha, mitochondrial	33 kDa	4.4947
22	PREDICTED: uncharacterized protein LOC100707031 isoform X1	12 kDa	4.2863
23	ADP-ribosylation factor-like protein 9	26 kDa	4.0831
24	prohibitin 2a	35 kDa	4.0018
25	band 3 anion transport protein	35 kDa	3.6752
26	PREDICTED: ATP synthase subunit gamma, mitochondrial isoform X1	33 kDa	3.4842
27	PREDICTED: coiled-coil-helix-coiled-coil-helix domain-containing protein 6, mitochondrial isoform X2	29 kDa	3.4397
28	calcium-binding mitochondrial carrier protein Aralar1	76 kDa	3.3774
29	dihydrolipoyllysine-residue acetyltransferase component of pyruvate dehydrogenase complex, mitochondrial	69 kDa	3.2991
30	PREDICTED: ammonium transporter Rh type A isoform X1	12 kDa	3.2045
31	ras-related protein Rab-1B	22 kDa	3.103
32	voltage-dependent anion-selective channel protein 2	20 kDa	3.0643
33	ATP synthase subunit O, mitochondrial	26 kDa	2.9888
34	PREDICTED: phosphate carrier protein, mitochondrial-like isoform X1	34 kDa	2.8384
35	calcium-binding mitochondrial carrier protein Aralar1	60 kDa	2.7242
36	cytochrome c-1	36 kDa	2.571
37	PREDICTED: tubulin beta-4B chain-like	31 kDa	2.5302
38	PREDICTED: ATPase, Na+/K+ transporting, beta 2b polypeptide isoform X1	24 kDa	2.3123
39	ras-related protein Rab-2A	24 kDa	2.3123
40	NADH dehydrogenase 1 beta subcomplex subunit 4	15 kDa	2.222
41	PREDICTED: NADH dehydrogenase	12 kDa	2.187
42	ES1 protein, mitochondrial precursor	31 kDa	2.1733
43	retinol dehydrogenase 13	37 kDa	2.1611
44	ATPase, Na+/K+ transporting, beta 2b polypeptide	34 kDa	2.155
45	flotillin-1	41 kDa	2.1371
46	flotillin-2a	47 kDa	2.1241
47	PREDICTED: calcium-binding mitochondrial carrier protein Aralar2 isoform X1	15 kDa	2.1192
48	histone 1, H4, like	12 kDa	2.0635
49	green-sensitive opsin-4	39 kDa	2.0402
50	PREDICTED: ras-related protein Rab-1A-like isoform X1	22 kDa	2.014
51	PREDICTED: NADH dehydrogenase	20 kDa	1.9289
52	creatine kinase b-type	16 kDa	1.9177

53	PREDICTED: creatine kinase S-type, mitochondrial isoform X2	47 kDa	1.9172
54	peripherin-2	20 kDa	1.8952
55	protein disulfide-isomerase TMX3 precursor	47 kDa	1.8814
56	uncharacterized protein LOC541492	13 kDa	1.8285
57	G-protein-coupled receptor kinase 7A	62 kDa	1.8236
58	PREDICTED: threonine dehydratase, mitochondrial-like isoform X2	60 kDa	1.7725
59	retinol dehydrogenase 8a	35 kDa	1.735
60	mitochondrial import inner membrane translocase subunit tim16	14 kDa	1.7187
61	erlin-2 precursor	40 kDa	1.6659
62	G-protein-coupled receptor kinase 7A	62 kDa	1.6653
63	reticulon-4	22 kDa	1.6103
64	ras-related protein Rab-1B	19 kDa	1.5373
65	sodium/potassium-transporting ATPase subunit alpha-3	113 kDa	1.4576
66	guanine nucleotide-binding protein G(o) subunit alpha	40 kDa	1.4444
67	NADH dehydrogenase 1 alpha subcomplex subunit 6	16 kDa	1.4242
68	PREDICTED: LOW QUALITY PROTEIN: actin, gamma 1	40 kDa	1.421
69	LETM1 and EF-hand domain-containing protein 1, mitochondrial	86 kDa	1.414
70	PREDICTED: ADP-ribosylation factor 2	16 kDa	1.4099
71	signal peptidase complex subunit 3	20 kDa	1.3911
72	PREDICTED: ras-related protein Rab-11B	25 kDa	1.3698
73	CDGSH iron sulfur domain 1	12 kDa	1.3672
74	ubiquitin-60S ribosomal protein L40	12 kDa	1.3672
75	ADP-ribosylation factor 1	21 kDa	1.3378
76	PREDICTED: vesicle-associated membrane protein 2-like	12 kDa	1.3324
77	long-chain fatty acid transport protein 4	25 kDa	1.3264
78	PREDICTED: calcium-binding mitochondrial carrier protein Aralar2 isoform X1	48 kDa	1.2667
79	reticulon-4	22 kDa	1.216
80	PREDICTED: prohibitin-2	27 kDa	1.2107
81	PREDICTED: mitochondrial import inner membrane translocase subunit Tim23	22 kDa	1.1991
82	PREDICTED: ras-related protein Rab-10	23 kDa	1.1746
83	band 3 anion transport protein	27 kDa	1.1697
84	PREDICTED: ATP synthase subunit beta, mitochondrial-like	56 kDa	1.1576
85	NADH-cytochrome b5 reductase 1	28 kDa	1.1502
86	RAB1A, member RAS oncogene family	28 kDa	1.1502
87	reticulon-4-interacting protein 1 homolog, mitochondrial	42 kDa	1.1361
88	PREDICTED: LOW QUALITY PROTEIN: 40S ribosomal protein S13-like	19 kDa	1.1243
89	RAB5A, member RAS oncogene family, a	24 kDa	1.1139
90	PREDICTED: ras-related protein Rab-6A isoform X2	24 kDa	1.1139
91	PREDICTED: succinate dehydrogenase cytochrome b560 subunit, mitochondrial isoform X1	19 kDa	1.1062
92	guanine nucleotide-binding protein subunit beta-5	43 kDa	1.0998
93	OCIA domain-containing protein 1	30 kDa	1.0506
94	PREDICTED: mitochondrial pyruvate carrier 2	14 kDa	1.049
95	putative tubulin beta chain variant 1	20 kDa	1.0392
96	protein RD3	15 kDa	1.0384
97	PREDICTED: voltage-dependent anion-selective channel protein 3 isoform X3	36 kDa	0.9969
98	PREDICTED: voltage-dependent anion-selective channel protein 3 isoform X3	36 kDa	0.9969
99	adipocyte plasma membrane-associated protein	47 kDa	0.9967
100	cytochrome b-c1 complex subunit 1, mitochondrial	52 kDa	0.9894
101	NADH dehydrogenase	58 kDa	0.9887
102	sorting and assembly machinery component 50 homolog A	52 kDa	0.9865
103	PREDICTED: dehydrogenase/reductase SDR family member 7B isoform X1	31 kDa	0.985
104	ras-related protein Rap-1b-like	21 kDa	0.9522
105	ADP-ribosylation factor-like protein 6	21 kDa	0.9522
106	NADH dehydrogenase 1 alpha subcomplex subunit 11	16 kDa	0.9516
107	PREDICTED: ubiquinol-cytochrome c reductase core protein II isoform X1	49 kDa	0.9416
108	creatine kinase U-type, mitochondrial	22 kDa	0.9018
109	PREDICTED: regulator of G-protein signaling 9 isoform X2	62 kDa	0.9007
110	PREDICTED: peripherin-2	39 kDa	0.8957
111	dolichol-phosphate mannosyltransferase subunit 1	28 kDa	0.8834

112	PREDICTED: transmembrane emp24 domain-containing protein 2	23 kDa	0.8563
113	ras-related protein Rab-8A	23 kDa	0.8563
114	serine/threonine-protein kinase MAK	11 kDa	0.8547
115	DDRGK domain-containing protein 1 precursor	35 kDa	0.8532
116	mitochondrial import receptor subunit TOM70	65 kDa	0.838
117	sorting and assembly machinery component 50 homolog B	23 kDa	0.8351
118	PREDICTED: 60S ribosomal protein L9 isoform X2	17 kDa	0.8278
119	HIG1 domain family member 1A	11 kDa	0.8235
120	ER membrane protein complex subunit 3	30 kDa	0.8153
121	ras-related protein Rab-5B	24 kDa	0.815
122	protein NDRG1 isoform 1	42 kDa	0.8101
123	very-long-chain enoyl-CoA reductase	36 kDa	0.8058
124	ras-related protein Rab-14	24 kDa	0.8053
125	PREDICTED: syntaxin-12 isoform X1	30 kDa	0.8037
126	protein THEM6 precursor	24 kDa	0.8005
127	PREDICTED: 40S ribosomal protein S8	24 kDa	0.7958
128	PREDICTED: tubulin alpha chain-like	12 kDa	0.7852
129	PREDICTED: mitochondrial fission process protein 1-like	18 kDa	0.7831
130	transmembrane protein 256 precursor	12 kDa	0.7762
131	transmembrane protein 256 precursor	12 kDa	0.7762
132	cytochrome b-c1 complex subunit 2, mitochondrial	50 kDa	0.7759
133	PREDICTED: reticulon-3-B-like isoform X2	25 kDa	0.7731
134	PREDICTED: saccharopine dehydrogenase-like oxidoreductase-like	12 kDa	0.7674
135	solute carrier family 3 (amino acid transporter heavy chain), member 2b	57 kDa	0.7645
136	PREDICTED: tubulin alpha chain	45 kDa	0.7509
137	dihydrolipoyllysine-residue succinyltransferase component of 2-oxoglutarate dehydrogenase complex, mitochondrial	51 kDa	0.748
138	calmegin precursor	66 kDa	0.7279
139	mitochondrial ATP synthase subunit f	13 kDa	0.7183
140	PREDICTED: ubiquinol-cytochrome c reductase core protein II isoform X1	40 kDa	0.7172
141	protein NipSnap homolog 2	33 kDa	0.712
142	NADH dehydrogenase (ubiquinone) 1 subunit c2	13 kDa	0.7107
143	transmembrane protein 11, mitochondrial	27 kDa	0.7043
144	PREDICTED: red-sensitive opsin-1 isoform X1	41 kDa	0.6974
145	cytochrome c oxidase subunit IV isoform 2	20 kDa	0.6919
146	PREDICTED: tubulin beta-2B chain-like isoform 1	55 kDa	0.6904
147	ras-related protein Rab-35	20 kDa	0.6779
148	PREDICTED: mitochondrial glutamate carrier 1	34 kDa	0.6776
149	PREDICTED: metal transporter CNNM4	35 kDa	0.6615
150	sideroflexin-3	36 kDa	0.6512
151	trifunctional enzyme subunit beta, mitochondrial	50 kDa	0.6511
152	PREDICTED: hexokinase-1-like	14 kDa	0.6489
153	succinyl-CoA ligase	51 kDa	0.6439
154	PREDICTED: NADH dehydrogenase	21 kDa	0.6433
155	mitochondrial import receptor subunit TOM40 homolog	36 kDa	0.6413
156	mitochondrial import receptor subunit TOM40 homolog	36 kDa	0.6364
157	dolichyl-diphosphooligosaccharide--protein glycosyltransferase 48 kDa subunit precursor	51 kDa	0.6301
158	peroxiredoxin-2	22 kDa	0.6233
159	PREDICTED: pyruvate dehydrogenase E1 alpha 1 isoform X1	45 kDa	0.62
160	60S ribosomal protein L9	22 kDa	0.6195
161	vesicle-associated membrane protein-associated protein A	30 kDa	0.6169
162	protein kinase, cAMP-dependent, regulatory, type II, alpha A	45 kDa	0.6162
163	retinoschisin precursor	22 kDa	0.6157
164	solute carrier family 2, facilitated glucose transporter member 1	53 kDa	0.6104
165	PREDICTED: 40S ribosomal protein S9-like	22 kDa	0.6009
166	membrane magnesium transporter 1 precursor	15 kDa	0.5968
167	uncharacterized protein LOC492355	23 kDa	0.5834
168	peripherin 2b (retinal degeneration, slow)	39 kDa	0.5832
169	mitochondrial chaperone BCS1	48 kDa	0.5706

170	elongation factor 1-alpha	48 kDa	0.5674
171	PREDICTED: ras-related protein Rab-7a isoform X1	24 kDa	0.5669
172	AFG3-like protein 2	89 kDa	0.565
173	uncharacterized protein C18orf19 homolog B	32 kDa	0.5577
174	PREDICTED: cyclic nucleotide-gated channel rod photoreceptor subunit alpha-like	82 kDa	0.5572
175	PREDICTED: guanine nucleotide-binding protein G(i) subunit alpha-1	40 kDa	0.556
176	PREDICTED: uncharacterized protein si:dkey-182g1.3	16 kDa	0.5479
177	mitochondrial inner membrane protein	83 kDa	0.5418
178	PREDICTED: LOW QUALITY PROTEIN: long-chain-fatty-acid--CoA ligase 6	50 kDa	0.5399
179	nicastrin precursor	42 kDa	0.538
180	arrestin-C	33 kDa	0.531
181	epidermal retinol dehydrogenase 2	34 kDa	0.5248
182	PREDICTED: guanine nucleotide-binding protein subunit alpha-13-like	34 kDa	0.5248
183	dynamin-1-like protein	77 kDa	0.5221
184	isocitrate dehydrogenase	43 kDa	0.5212
185	brain creatine kinase b	43 kDa	0.518
186	inactive hydroxysteroid dehydrogenase-like protein 1	35 kDa	0.5068
187	dehydrogenase/reductase (SDR family) member 13a, duplicate 3	35 kDa	0.5068
188	uncharacterized protein LOC100135257	35 kDa	0.503
189	40S ribosomal protein S7	17 kDa	0.5024
190	PREDICTED: vesicle-associated membrane protein-associated protein A-like	26 kDa	0.499
191	PREDICTED: vesicle-associated membrane protein-associated protein A-like	26 kDa	0.499
192	PREDICTED: nucleoside diphosphate kinase B isoform X1	17 kDa	0.4987
193	NADH dehydrogenase	53 kDa	0.4981
194	hexokinase-1	71 kDa	0.4976
195	PREDICTED: gamma-glutamyltransferase 5 isoform X1	62 kDa	0.4973
196	PREDICTED: tubulin alpha-1C chain	45 kDa	0.4934
197	NAD-dependent protein deacetylase sirtuin-3, mitochondrial	18 kDa	0.4877
198	PREDICTED: 60S ribosomal protein L12	18 kDa	0.4841
199	optic atrophy 3 protein homolog	18 kDa	0.4806
200	PREDICTED: transmembrane emp24 domain-containing protein 9 isoform X1	27 kDa	0.4798
201	calcium/calmodulin-dependent protein kinase type II delta 1 chain isoform 1	56 kDa	0.4722
202	uncharacterized protein LOC100127838	18 kDa	0.4671
203	very-long-chain (3R)-3-hydroxyacyl-CoA dehydratase 2	28 kDa	0.4664
204	PREDICTED: cyclic nucleotide-gated cation channel beta-3-like isoform X2	28 kDa	0.4664
205	40S ribosomal protein S18	18 kDa	0.4639
206	transmembrane protein 33	29 kDa	0.4516
207	carbonic anhydrase	29 kDa	0.4495
208	PREDICTED: ADP-dependent glucokinase isoform X2	58 kDa	0.4478
209	PREDICTED: sodium/potassium/calcium exchanger 2-like isoform X2	68 kDa	0.4467
210	ATPase family AAA domain-containing protein 3	69 kDa	0.4441
211	opsin-1, short-wave-sensitive 2	39 kDa	0.4398
212	opsin-1, short-wave-sensitive 1	39 kDa	0.4398
213	PREDICTED: L-lactate dehydrogenase B-B chain isoform X4	19 kDa	0.4364
214	PREDICTED: L-lactate dehydrogenase B-B chain isoform X3	19 kDa	0.4336
215	cytochrome c oxidase subunit 4 isoform 1, mitochondrial	20 kDa	0.4308
216	PREDICTED: tubulin alpha chain-like	50 kDa	0.4307
217	plasminogen receptor (KT)	20 kDa	0.428
218	ras-related protein Rab-8B	20 kDa	0.428
219	eukaryotic translation elongation factor 1 alpha 1-like	50 kDa	0.4274
220	PREDICTED: potassium voltage-gated channel subfamily B member 2	93 kDa	0.4184
221	NADH dehydrogenase 1 beta subcomplex subunit 10	20 kDa	0.4172
222	PREDICTED: ADP-ribosylation factor 6-like	20 kDa	0.4172
223	mitochondrial carrier homolog 2	31 kDa	0.414
224	ADP-ribosylation factor-like protein 1	20 kDa	0.412
225	PREDICTED: cadherin-related family member 5-like isoform X2	20 kDa	0.4095
226	PREDICTED: epoxide hydrolase 1-like, partial	20 kDa	0.4095
227	immunity-related GTPase family, q2	42 kDa	0.4074
228	LIM domain and actin-binding protein 1	20 kDa	0.407

229	heat shock cognate 71 kDa protein	42 kDa	0.4049
230	zinc transporter 9	64 kDa	0.4025
231	mitochondrial dicarboxylate carrier	32 kDa	0.4023
232	ras-related protein Rap-1b precursor	21 kDa	0.4021
233	hexokinase-1	54 kDa	0.3958
234	cone cGMP-specific 3',5'-cyclic phosphodiesterase subunit alpha'	98 kDa	0.3931
235	uncharacterized protein LOC393228	21 kDa	0.3902
236	uncharacterized protein LOC100302470 precursor	21 kDa	0.3902
237	synaptophysin b isoform 1	33 kDa	0.3866
238	PREDICTED: complex I assembly factor TIMMDC1, mitochondrial isoform X1	33 kDa	0.3793
239	dolichyl-diphosphooligosaccharide--protein glycosyltransferase subunit 1 precursor	67 kDa	0.3773
240	neurocalcin-delta B	22 kDa	0.3769
241	ras homolog gene family, member A	22 kDa	0.3727
242	uncharacterized protein LOC100127828	34 kDa	0.3653
243	PREDICTED: cyclic nucleotide-gated channel cone photoreceptor subunit alpha isoform X2	81 kDa	0.364
244	heat shock protein 75 kDa, mitochondrial	82 kDa	0.3629
245	synaptotagmin II	47 kDa	0.3608
246	PREDICTED: retinol dehydrogenase 8-like isoform X1	35 kDa	0.36
247	phosphatidylglycerophosphatase and protein-tyrosine phosphatase 1	11 kDa	0.3579
248	PREDICTED: growth hormone-inducible transmembrane protein	35 kDa	0.3574
249	ras-related protein Rab-18-B	23 kDa	0.3566
250	PREDICTED: [Pyruvate dehydrogenase (acetyl-transferring)] kinase isozyme 1, mitochondrial	47 kDa	0.3559
251	PREDICTED: flotillin 1a isoform X1	47 kDa	0.355
252	uncharacterized protein LOC100135302	23 kDa	0.3547
253	ras-related protein Rab-43	23 kDa	0.3547
254	PREDICTED: uncharacterized protein LOC101884052	11 kDa	0.3541
255	surfeit gene 4, like	23 kDa	0.3528
256	methylmalonate-semialdehyde dehydrogenase	61 kDa	0.3447
257	vitamin K epoxide reductase complex subunit 1-like protein 1	12 kDa	0.3431
258	retinol dehydrogenase-like	36 kDa	0.3426
259	CDP-diacylglycerol--inositol 3-phosphatidyltransferase	24 kDa	0.3418
260	protein THEM6 precursor	24 kDa	0.3401
261	28S ribosomal protein S36, mitochondrial	12 kDa	0.3396
262	guanine nucleotide binding protein (G protein), beta polypeptide 3b	37 kDa	0.3391
263	NADH dehydrogenase	24 kDa	0.3384
264	PREDICTED: dephospho-CoA kinase domain-containing protein-like isoform X2	24 kDa	0.3384
265	PREDICTED: ras-related protein Rab-35-like	24 kDa	0.3384
266	PREDICTED: LOW QUALITY PROTEIN: long-chain-fatty-acid--CoA ligase 6	75 kDa	0.3353
267	phosphatidylglycerophosphatase and protein-tyrosine phosphatase 1	12 kDa	0.3327
268	NAD(P) transhydrogenase, mitochondrial	114 kDa	0.3299
269	40S ribosomal protein S5	25 kDa	0.3299
270	PREDICTED: uncharacterized protein LOC571872 isoform X1	12 kDa	0.3294
271	PREDICTED: Fc receptor-like protein 5 isoform X2	12 kDa	0.3294
272	PREDICTED: NADH dehydrogenase	25 kDa	0.3283
273	guanylyl cyclase 3 (guanylate cyclase retinal cone [Cyprinus carpio])	128 kDa	0.327
274	citrate synthase, mitochondrial precursor	52 kDa	0.3198
275	PREDICTED: SH3-containing GRB2-like protein 3-interacting protein 1 isoform X2	12 kDa	0.3168
276	synaptic vesicle glycoprotein 2B	12 kDa	0.3168
277	erlin-1 precursor	39 kDa	0.3154
278	stomatin-like protein 2, mitochondrial	39 kDa	0.3134
279	PREDICTED: ADP-dependent glucokinase isoform X2	26 kDa	0.3113
280	mitochondrial pyruvate carrier 1	13 kDa	0.3108
281	pyruvate dehydrogenase E1 component subunit alpha, somatic form, mitochondrial	13 kDa	0.3108
282	PREDICTED: phospholipid scramblase 2	26 kDa	0.3069
283	PREDICTED: uncharacterized protein LOC100334801 isoform X2	26 kDa	0.3069
284	cytochrome c oxidase subunit 6A1, mitochondrial	13 kDa	0.3051
285	guanine nucleotide-binding protein G(i) subunit alpha-2	41 kDa	0.3028
286	PREDICTED: mitochondrial import inner membrane translocase subunit Tim21	27 kDa	0.3027

287	phosducin	27 kDa	0.3
288	ubiquitin-like 3b	13 kDa	0.2996
289	mitochondrial trifunctional protein, alpha subunit	83 kDa	0.2989
290	PREDICTED: protein MGARP isoform X2	41 kDa	0.2965
291	mannose-P-dolichol utilization defect 1 protein	27 kDa	0.2959
292	PREDICTED: transmembrane emp24 domain-containing protein 7 isoform X2	27 kDa	0.2946
293	PREDICTED: 40S ribosomal protein S20	13 kDa	0.2942
294	PREDICTED: proteoglycan 4-like isoform X1	13 kDa	0.2916
295	PREDICTED: prostate stem cell antigen-like	13 kDa	0.2916
296	ras-related GTP-binding protein C	13 kDa	0.2916
297	60S ribosomal protein L8	28 kDa	0.2869
298	ATP synthase F(0) complex subunit C3, mitochondrial	14 kDa	0.2866
299	ras-related protein Rab-3A	28 kDa	0.2844
300	PREDICTED: NADH dehydrogenase	43 kDa	0.2821
301	60S ribosomal protein L7	29 kDa	0.282
302	monoacylglycerol lipase ABHD12	44 kDa	0.2797
303	PREDICTED: neuroplastin-like	44 kDa	0.2797
304	histone 2, H2a	14 kDa	0.2793
305	60S ribosomal protein L30	14 kDa	0.2793
306	40S ribosomal protein S4, X isoform	29 kDa	0.2761
307	PREDICTED: lipoamide acyltransferase component of branched-chain alpha-keto acid dehydrogenase complex, mitochondrial-like	45 kDa	0.2706
308	60S ribosomal protein L7a	30 kDa	0.2672
309	PREDICTED: NADH dehydrogenase	15 kDa	0.2658
310	PREDICTED: multiple PDZ domain protein isoform X1	93 kDa	0.2638
311	PREDICTED: ammonium transporter Rh type A-like	15 kDa	0.2637
312	26S protease regulatory subunit 8	46 kDa	0.2634
313	aquaporin 1	30 kDa	0.2629
314	PREDICTED: 40S ribosomal protein S15a isoform X1	15 kDa	0.2616
315	PREDICTED: ankyrin 1, erythrocytic a isoform X12	31 kDa	0.2578
316	PREDICTED: GTPase HRas-like	15 kDa	0.2575
317	PREDICTED: equilibrative nucleoside transporter 1-like	15 kDa	0.2575
318	enoyl-CoA hydratase, mitochondrial	31 kDa	0.2558
319	PREDICTED: ER membrane protein complex subunit 1 isoform X1	111 kDa	0.2557
320	40S ribosomal protein S25	15 kDa	0.2555
321	NADH-ubiquinone oxidoreductase 75 kDa subunit, mitochondrial	80 kDa	0.254
322	aspartate aminotransferase 2a	48 kDa	0.2539
323	Beta-centractin	15 kDa	0.2535
324	PREDICTED: probable signal peptidase complex subunit 2-like isoform X1	15 kDa	0.2535
325	tubulin, alpha 8 like	48 kDa	0.252
326	PREDICTED: carnitine O-palmitoyltransferase 1, liver isoform isoform X2	48 kDa	0.2482
327	PREDICTED: gamma-aminobutyric acid receptor subunit alpha-6 isoform X1	49 kDa	0.2463
328	PREDICTED: uncharacterized protein LOC571872 isoform X1	32 kDa	0.2453
329	cytochrome c oxidase subunit Vaa	16 kDa	0.2441
330	60S ribosomal protein L28	16 kDa	0.2441
331	microsomal glutathione S-transferase-like	16 kDa	0.2441
332	elongation factor Tu, mitochondrial	49 kDa	0.2427
333	brain creatine kinase	16 kDa	0.2423
334	PREDICTED: FUN14 domain-containing protein 2 isoform X1	16 kDa	0.2388
335	PREDICTED: mitochondrial ubiquitin ligase activator of nfkb 1-A	33 kDa	0.2382
336	RPE-retinal G protein-coupled receptor	33 kDa	0.2382
337	isocitrate dehydrogenase	50 kDa	0.238
338	mitochondrial import receptor subunit TOM20 homolog B	16 kDa	0.2371
339	elongation factor Ts, mitochondrial	34 kDa	0.2365
340	ubiquitin-conjugating enzyme E2Nb	16 kDa	0.2354
341	alpha/beta hydrolase domain-containing protein 11	34 kDa	0.2323
342	ras-related protein Rab-27A	17 kDa	0.2321
343	mitochondrial import inner membrane translocase subunit TIM44	52 kDa	0.2308
344	PREDICTED: equilibrative nucleoside transporter 1-like	34 kDa	0.2307
345	NADH dehydrogenase	17 kDa	0.2305

346	chaperone activity of bc1 complex-like, mitochondrial	70 kDa	0.23
347	PREDICTED: retinol dehydrogenase 12-like	34 kDa	0.2299
348	retinal G protein coupled receptor b	35 kDa	0.2291
349	PREDICTED: NADH dehydrogenase	17 kDa	0.2289
350	PREDICTED: protein transport protein Sec61 subunit alpha-like 1	52 kDa	0.2287
351	ubiquinone biosynthesis monooxygenase COQ6	52 kDa	0.2276
352	G protein-coupled receptor kinase 1 b	17 kDa	0.2242
353	PREDICTED: coiled-coil domain-containing protein 136-like isoform X1	53 kDa	0.2235
354	CAAX prenyl protease 1 homolog	53 kDa	0.2235
355	PREDICTED: 40S ribosomal protein S23-like	17 kDa	0.2227
356	dihydrolipoyl dehydrogenase, mitochondrial	54 kDa	0.222
357	PREDICTED: protein FAM162B isoform X1	17 kDa	0.2212
358	PREDICTED: 40S ribosomal protein S24	17 kDa	0.2212
359	PREDICTED: 60S ribosomal protein L23	17 kDa	0.2212
360	microsomal glutathione S-transferase 1.2	17 kDa	0.2212
361	translocase of outer mitochondrial membrane 40 homolog, like	36 kDa	0.2199
362	putative Ras-related protein Rab-42	18 kDa	0.2197
363	ragulator complex protein LAMTOR1	18 kDa	0.2197
364	microsomal glutathione S-transferase 1.1	18 kDa	0.2197
365	long-chain fatty acid transport protein 4	73 kDa	0.2197
366	lipoamide acyltransferase component of branched-chain alpha-keto acid dehydrogenase complex, mitochondrial	54 kDa	0.2195
367	60S ribosomal protein L14	18 kDa	0.2183
368	PREDICTED: fatty aldehyde dehydrogenase-like	55 kDa	0.2171
369	L-lactate dehydrogenase B-A chain	36 kDa	0.217
370	60S ribosomal protein L24	18 kDa	0.2168
371	60S ribosomal protein L26	18 kDa	0.2168
372	60S ribosomal protein L27a	18 kDa	0.2154
373	PREDICTED: ankyrin-1-like	18 kDa	0.2154
374	PREDICTED: multiple PDZ domain protein isoform X1	37 kDa	0.2142
375	mitochondrial import inner membrane translocase subunit Tim17-B	18 kDa	0.214
376	ATP synthase subunit d, mitochondrial	18 kDa	0.2113
377	ras-related protein Rab-5C	37 kDa	0.2108
378	uncharacterized protein LOC559844	37 kDa	0.2101
379	PREDICTED: epoxide hydrolase 1, partial	38 kDa	0.2088
380	40S ribosomal protein S16	18 kDa	0.2086
381	PREDICTED: peptidyl-prolyl cis-trans isomerase FKBP8 isoform X1	57 kDa	0.2075
382	neurotrimin isoform 1 precursor	38 kDa	0.2062
383	2-methoxy-6-polyprenyl-1,4-benzoquinol methylase, mitochondrial precursor	38 kDa	0.2049
384	PREDICTED: serine/threonine-protein kinase DCLK1 isoform X1	38 kDa	0.2042
385	acyl-CoA synthetase long-chain family member 3b	79 kDa	0.2006
386	PREDICTED: NCK-interacting protein with SH3 domain-like	19 kDa	0.1997
387	EF-hand calcium-binding domain-containing protein 4A	19 kDa	0.1997
388	PREDICTED: ceroid-lipofuscinosis, neuronal 6a isoform X1	19 kDa	0.1973
389	NADH dehydrogenase	41 kDa	0.1929
390	cytochrome P450, family 27, subfamily C, polypeptide 1	62 kDa	0.1914
391	PREDICTED: cadherin-related family member 1 isoform X2	83 kDa	0.1902
392	uncharacterized protein LOC100158473	20 kDa	0.1894
393	PREDICTED: sterol 26-hydroxylase, mitochondrial	20 kDa	0.1872
394	ER membrane protein complex subunit 4	20 kDa	0.1872
395	phosphatidylinositol N-acetylglucosaminyltransferase subunit H	20 kDa	0.1872
396	PREDICTED: signal peptidase complex catalytic subunit SEC11A-like	21 kDa	0.1862
397	PREDICTED: protein XRP2 isoform X1	42 kDa	0.1853
398	PREDICTED: protein TsetseEP-like	21 kDa	0.1851
399	uncharacterized protein LOC570464 precursor	21 kDa	0.1851
400	enoyl-CoA delta isomerase 2, mitochondrial	42 kDa	0.1837
401	calpain-5	21 kDa	0.1831
402	PREDICTED: vesicle-fusing ATPase isoform X1	86 kDa	0.1825
403	PREDICTED: translocon-associated protein subunit gamma	21 kDa	0.181
404	PREDICTED: cell division control protein 42 homolog isoform X1	21 kDa	0.1791

405	aspartate aminotransferase 2	43 kDa	0.1787
406	PREDICTED: glutaminase a isoform X1	66 kDa	0.1786
407	PREDICTED: transmembrane and coiled-coil domains protein 1-like	21 kDa	0.1781
408	ADP-ribosylation factor-like protein 8B-A	21 kDa	0.1781
409	phosphatidylserine synthase 1	21 kDa	0.1781
410	PREDICTED: cadherin-related family member 5-like isoform X2	44 kDa	0.1773
411	ADP-ribosylation-like factor 6 interacting protein 5	22 kDa	0.1771
412	outer dense fiber of sperm tails 2b	22 kDa	0.1771
413	peptidyl-prolyl cis-trans isomerase FKBP8	44 kDa	0.1768
414	mitochondrial NADH dehydrogenase (ubiquinone) 1 beta subcomplex subunit 5	22 kDa	0.1753
415	Bcl-2/adenovirus E1B 19kD interaction protein XR	22 kDa	0.1753
416	ADP-ribosylation factor-like 3, like 1	22 kDa	0.1753
417	PREDICTED: guanine nucleotide-binding protein G(s) subunit alpha isoform X2	44 kDa	0.1749
418	recoverin-like	22 kDa	0.1743
419	lactation elevated protein 1 homolog B	22 kDa	0.1743
420	ubiquinone biosynthesis protein COQ7 homolog	22 kDa	0.1734
421	PREDICTED: protein RER1 isoform X2	22 kDa	0.1734
422	rho-related gtp-binding protein rhoc	22 kDa	0.1734
423	synaptobrevin homolog YKT6	22 kDa	0.1707
424	PREDICTED: transmembrane protein 126A isoform X1	22 kDa	0.1698
425	PREDICTED: paraplegin	92 kDa	0.1696
426	F-box/LRR-repeat protein 2	46 kDa	0.1691
427	PREDICTED: flavin reductase (NADPH)-like	23 kDa	0.169
428	ras-related protein Rab-18	23 kDa	0.1681
429	PREDICTED: protein NDRG3 isoform X1	46 kDa	0.1678
430	bcl2-associated X protein, b	23 kDa	0.1672
431	PREDICTED: pyruvate dehydrogenase kinase, isozyme 3 isoform X2	46 kDa	0.1669
432	PREDICTED: regulator of microtubule dynamics protein 2 isoform X1	47 kDa	0.1665
433	PREDICTED: cadherin-related family member 5-like isoform X2	23 kDa	0.1664
434	coiled-coil domain-containing protein 51	47 kDa	0.1649
435	saccharopine dehydrogenase b	47 kDa	0.1649
436	PREDICTED: prominin-1 isoform X1	95 kDa	0.1637
437	PREDICTED: ADP-ribosylation factor-like protein 3	23 kDa	0.1631
438	apolipoprotein O	24 kDa	0.1615
439	PREDICTED: sphingomyelin phosphodiesterase 2 isoform X1	48 kDa	0.1608
440	PREDICTED: NADPH--cytochrome P450 reductase isoform X1	48 kDa	0.1608
441	NADH dehydrogenase	48 kDa	0.1604
442	[3-methyl-2-oxobutanoate dehydrogenase	48 kDa	0.16
443	60S ribosomal protein L15	24 kDa	0.1584
444	PREDICTED: metal transporter CNNM4	74 kDa	0.1578
445	ceramide-1-phosphate transfer protein	24 kDa	0.1576
446	60S ribosomal protein L10	25 kDa	0.1547
447	PREDICTED: ras-related protein Rab-28 isoform X2	25 kDa	0.1532
448	60S ribosomal protein L10a	25 kDa	0.1525
449	PREDICTED: von Willebrand factor A domain-containing protein 1	25 kDa	0.1525
450	peroxisomal membrane protein 11B	25 kDa	0.1518
451	hydroxysteroid dehydrogenase-like protein 2	51 kDa	0.1516
452	PREDICTED: V-type proton ATPase subunit S1	52 kDa	0.1492
453	protein disulfide-isomerase TMX3 precursor	52 kDa	0.1481
454	epoxide hydrolase 1	52 kDa	0.1478
455	Probable saccharopine dehydrogenase	26 kDa	0.1464
456	PREDICTED: OCIA domain-containing protein 1 isoform X1	26 kDa	0.1451
457	PREDICTED: mitochondrial fission factor-like isoform X3	26 kDa	0.1451
458	PREDICTED: sodium-coupled neutral amino acid transporter 3	53 kDa	0.1449
459	PREDICTED: sarcoplasmic/endoplasmic reticulum calcium ATPase 3 isoform X2	53 kDa	0.1446
460	PREDICTED: abhydrolase domain-containing protein 8	53 kDa	0.1439
461	bcl-2-like protein 1	26 kDa	0.1432
462	NADH dehydrogenase	27 kDa	0.1426
463	PREDICTED: ATP synthase subunit s-like protein isoform X1	27 kDa	0.142

464	ATP synthase subunit s, mitochondrial	27 kDa	0.1414
465	ER membrane protein complex subunit 7 precursor	27 kDa	0.1414
466	G protein-coupled receptor kinase 1 b	54 kDa	0.1411
467	sorting and assembly machinery component 50 homolog B	27 kDa	0.1384
468	transmembrane emp24 domain-containing protein 4 precursor	27 kDa	0.1384
469	PREDICTED: sodium-coupled neutral amino acid transporter 3-like isoform X1	56 kDa	0.1356
470	large neutral amino acids transporter small subunit 1	57 kDa	0.1348
471	uncharacterized protein C2orf47 homolog, mitochondrial	28 kDa	0.1344
472	ER membrane protein complex subunit 10 isoform 1 precursor	28 kDa	0.1344
473	serine hydroxymethyltransferase, mitochondrial	57 kDa	0.1334
474	3-hydroxyacyl-CoA dehydrogenase type-2	28 kDa	0.1333
475	thioredoxin-dependent peroxide reductase, mitochondrial	28 kDa	0.1333
476	PREDICTED: 40S ribosomal protein S3-like isoform X1	28 kDa	0.1328
477	PREDICTED: solute carrier family 1 (glial high affinity glutamate transporter), member 2a isoform X2	58 kDa	0.1323
478	aquaporin-9	29 kDa	0.1323
479	PREDICTED: olfactory guanylyl cyclase GC-D isoform X3 (guanylate cyclase retinal rod1 [Cyprinus carpio])	88 kDa	0.1313
480	UDP glucuronosyltransferase 1 family, polypeptide B1 precursor	29 kDa	0.1312
481	heat shock protein HSP 90-beta	89 kDa	0.1303
482	PREDICTED: lysophosphatidylcholine acyltransferase 1-like	59 kDa	0.13
483	S-adenosylmethionine mitochondrial carrier protein	29 kDa	0.1297
484	neuronal membrane glycoprotein M6-b	30 kDa	0.1276
485	calpain-5	30 kDa	0.1262
486	erythrocyte membrane protein band 4.1b (elliptocytosis 1, RH-linked)	30 kDa	0.1257
487	uncharacterized protein LOC447917 precursor	30 kDa	0.1247
488	prominin-1 precursor	94 kDa	0.123
489	40S ribosomal protein S6	31 kDa	0.1224
490	methyltransferase like 7A precursor	31 kDa	0.1215
491	signal recognition particle receptor subunit beta	31 kDa	0.1211
492	eukaryotic translation initiation factor 3 subunit F	31 kDa	0.1206
493	E3 ubiquitin-protein ligase MARCH5	31 kDa	0.1197
494	erythrocyte band 7 integral membrane protein	32 kDa	0.1193
495	surfeit locus protein 1	32 kDa	0.118
496	integral membrane protein 2B	32 kDa	0.118
497	thioredoxin-related transmembrane protein 1 precursor	32 kDa	0.1176
498	PREDICTED: cadherin-related family member 1-like isoform X2	99 kDa	0.1164
499	PREDICTED: rap1 GTPase-GDP dissociation stimulator 1-like isoform X1	32 kDa	0.1163
500	PREDICTED: translocon-associated protein subunit alpha-like isoform X3	32 kDa	0.1155
501	PREDICTED: aspartate beta-hydroxylase isoform X2	33 kDa	0.1143
502	ubiquinol-cytochrome c reductase complex assembly factor 1	33 kDa	0.1139
503	PREDICTED: mitogen-activated protein kinase 14A-like isoform X2	33 kDa	0.1128
504	PREDICTED: calnexin isoform X1	67 kDa	0.1126
505	PREDICTED: enoyl-CoA delta isomerase 1, mitochondrial	33 kDa	0.1124
506	trifunctional enzyme subunit alpha, mitochondrial	33 kDa	0.1124
507	PREDICTED: protein CLN8 isoform X1	34 kDa	0.1116
508	protein-S-isoprenylcysteine O-methyltransferase	34 kDa	0.1109
509	PREDICTED: mitochondrial fission factor homolog B isoform X1	34 kDa	0.109
510	mitochondrial Rho GTPase 2	70 kDa	0.1089
511	dolichyl-diphosphooligosaccharide--protein glycosyltransferase subunit 2 precursor	70 kDa	0.1083
512	peroxisomal membrane protein PMP34	35 kDa	0.1076
513	elongation of very long chain fatty acids-like 4	35 kDa	0.1072
514	PREDICTED: very long-chain specific acyl-CoA dehydrogenase, mitochondrial isoform X1	71 kDa	0.1062
515	malate dehydrogenase, mitochondrial	35 kDa	0.1059
516	iron-sulfur protein NUBPL	36 kDa	0.1052
517	thioredoxin-related transmembrane protein 2-B precursor	36 kDa	0.1048
518	disintegrin and metalloproteinase domain-containing protein 10 precursor	36 kDa	0.1048
519	stress-70 protein, mitochondrial	73 kDa	0.1036
520	protein SCO2 homolog, mitochondrial	36 kDa	0.1032

521	DnaJ (Hsp40) homolog, subfamily A, member 3B	36 kDa	0.1032
522	PREDICTED: transmembrane protein 43-like	37 kDa	0.1022
523	phosducin-like protein	37 kDa	0.1019
524	transferrin receptor protein 1	77 kDa	0.0987
525	ATP-binding cassette sub-family B member 8, mitochondrial	77 kDa	0.0986
526	abhydrolase domain-containing protein 16A	38 kDa	0.098
527	ATPase asna1	38 kDa	0.0977
528	cyclin-Y-like protein 1	39 kDa	0.0971
529	heterogeneous nuclear ribonucleoprotein K	39 kDa	0.0962
530	PREDICTED: hydroxymethylbilane synthase a isoform X1	39 kDa	0.0962
531	Bardet-Biedl syndrome 5 protein homolog	39 kDa	0.096
532	eukaryotic translation initiation factor 4A, isoform 1A	39 kDa	0.0957
533	pyruvate dehydrogenase E1 component subunit beta, mitochondrial	39 kDa	0.0951
534	mitofusin-2	80 kDa	0.095
535	lectin, mannose-binding 2-like b precursor	40 kDa	0.0943
536	isocitrate dehydrogenase	40 kDa	0.0935
537	major histocompatibility complex class I UEA precursor	41 kDa	0.0904
538	PREDICTED: synaptotagmin-14 isoform X1	41 kDa	0.0904
539	PREDICTED: annexin A4-like isoform X1	42 kDa	0.0894
540	1-acyl-sn-glycerol-3-phosphate acyltransferase epsilon	42 kDa	0.0889
541	PREDICTED: guanine nucleotide-binding protein subunit alpha-11-like isoform X1	42 kDa	0.0887
542	PREDICTED: ADP-ribosylation factor-like protein 13B-like isoform X5	42 kDa	0.0885
543	HLA-B associated transcript 1	42 kDa	0.088
544	solute carrier family 43 member 3	43 kDa	0.0861
545	reticulon-1 isoform 1	88 kDa	0.0857
546	transducin beta-like protein 2 precursor	44 kDa	0.085
547	ATPase family AAA domain-containing protein 1-A isoform 1	44 kDa	0.0848
548	PREDICTED: mitogen-activated protein kinase 3	44 kDa	0.0844
549	dnaJ homolog subfamily A member 3, mitochondrial	44 kDa	0.0842
550	serine/threonine-protein kinase DCLK2 isoform 1	90 kDa	0.0838
551	basigin precursor	45 kDa	0.0835
552	GPI-anchor transamidase precursor	45 kDa	0.0835
553	neuroplastin precursor	45 kDa	0.0833
554	1-acyl-sn-glycerol-3-phosphate acyltransferase gamma	45 kDa	0.0825
555	endoplasmin precursor	92 kDa	0.0821
556	28S ribosomal protein S27, mitochondrial	46 kDa	0.0818
557	glycosaminoglycan xylosylkinase	46 kDa	0.0812
558	26S proteasome non-ATPase regulatory subunit 3	48 kDa	0.0781
559	PREDICTED: protein tweety homolog 3 isoform X1	48 kDa	0.078
560	PREDICTED: kynurenine/alpha-aminoadipate aminotransferase, mitochondrial isoform X1	48 kDa	0.0778
561	uncharacterized protein C6orf136 homolog	48 kDa	0.0774
562	PREDICTED: required for meiotic nuclear division protein 1 homolog isoform X1	48 kDa	0.0772
563	PREDICTED: apoptosis-inducing factor 1, mitochondrial isoform X1	48 kDa	0.0767
564	keratin, type I cytoskeletal 18	49 kDa	0.0763
565	PREDICTED: calcium-binding mitochondrial carrier protein SCaMC-2-B isoform X2	49 kDa	0.076
566	acyl-CoA dehydrogenase-like	49 kDa	0.076
567	adenylyl cyclase-associated protein 1	50 kDa	0.0751
568	calcium/calmodulin-dependent protein kinase IGa	50 kDa	0.0748
569	erythrocyte membrane protein band 4.1b (elliptocytosis 1, RH-linked)	50 kDa	0.0744
570	elongation factor 1-gamma	51 kDa	0.0731
571	UBX domain-containing protein 4	52 kDa	0.072
572	cytochrome P450, family 20, subfamily A, polypeptide 1	52 kDa	0.072
573	PREDICTED: metal transporter CNNM4	52 kDa	0.072
574	PREDICTED: phosphatidate cytidylyltransferase 1	52 kDa	0.0715
575	vimentin	53 kDa	0.0707
576	26S protease regulatory subunit 7	52 kDa	0.0707
577	squalene synthase	53 kDa	0.0704
578	zinc transporter 1	54 kDa	0.0693
579	PREDICTED: pyruvate dehydrogenase protein X component, mitochondrial	54 kDa	0.0688

580	PREDICTED: FAS-associated factor 2-like isoform X1	54 kDa	0.0687
581	PREDICTED: monocarboxylate transporter 4 isoform X1	55 kDa	0.0681
582	WD repeat-containing protein 37	55 kDa	0.0678
583	V-type proton ATPase subunit H isoform 1	55 kDa	0.0673
584	dynamin-like 120 kDa protein, mitochondrial precursor	112 kDa	0.0671
585	6-phosphogluconate dehydrogenase, decarboxylating isoform 1	56 kDa	0.0659
586	dol-P-Man:Man(7)GlcNAc(2)-PP-Dol alpha-1,6-mannosyltransferase precursor	56 kDa	0.0658
587	annexin 11a isoform 2	56 kDa	0.0658
588	PREDICTED: protein ERGIC-53	57 kDa	0.0646
589	neutral amino acid transporter B(0)	57 kDa	0.0646
590	PREDICTED: protein phosphatase 3, catalytic subunit, gamma isoform-like isoform X1	57 kDa	0.0646
591	T-complex protein 1 subunit beta	58 kDa	0.064
592	prenylcysteine oxidase 1 precursor	59 kDa	0.0624
593	fatty-acid amide hydrolase 2-A	59 kDa	0.0624
594	PREDICTED: pyruvate kinase PKM isoform X1	60 kDa	0.0617
595	PREDICTED: extended synaptotagmin-1 isoform X1	60 kDa	0.0615
596	lysine--tRNA ligase	60 kDa	0.0614
597	keratin, type II cytoskeletal 8	61 kDa	0.0612
598	PREDICTED: ankyrin-1-like isoform X2	184 kDa	0.0611
599	PREDICTED: cGMP-gated cation channel alpha-1	63 kDa	0.0587
600	dnaJ homolog subfamily C member 11	64 kDa	0.058
601	leucine-rich repeat, immunoglobulin-like and transmembrane domains 1 precursor	64 kDa	0.0579
602	phosphatidylinositide phosphatase SAC1-B	67 kDa	0.0549
603	PREDICTED: V-type proton ATPase catalytic subunit A	68 kDa	0.0541
604	PREDICTED: carnitine palmitoyltransferase 1A isoform X2	69 kDa	0.0539
605	acyl-CoA dehydrogenase family member 9, mitochondrial	69 kDa	0.0537
606	PREDICTED: aarF domain-containing protein kinase 4	70 kDa	0.0532
607	solute carrier family 7, member 3	70 kDa	0.0531
608	polyadenylate-binding protein 1	71 kDa	0.0522
609	PREDICTED: signal recognition particle receptor subunit alpha	71 kDa	0.0522
610	cleft lip and palate transmembrane protein 1 homolog	72 kDa	0.0516
611	long-chain fatty acid transport protein 1	72 kDa	0.0515
612	78 kDa glucose-regulated protein precursor	72 kDa	0.0512
613	succinate dehydrogenase	73 kDa	0.0508
614	PREDICTED: caseinolytic peptidase B protein homolog	74 kDa	0.0497
615	PREDICTED: ATP-dependent RNA helicase DDX3X isoform X6	79 kDa	0.0467
616	MAGUK p55 subfamily member 5-A	80 kDa	0.0462
617	PREDICTED: ATP-binding cassette sub-family B member 7, mitochondrial	82 kDa	0.0451
618	PREDICTED: ribosomal protein S6 kinase alpha-3 isoform X1	83 kDa	0.0443
619	methylmalonyl-CoA mutase, mitochondrial	84 kDa	0.0441
620	transferrin receptor 1b	86 kDa	0.043
621	PREDICTED: dolichyl-diphosphooligosaccharide--protein glycosyltransferase subunit STT3B	91 kDa	0.0404
622	PREDICTED: ATPase, Ca++ transporting, cardiac muscle, slow twitch 2b isoform X1	92 kDa	0.0403
623	PREDICTED: neural cell adhesion molecule 1b isoform X11	92 kDa	0.0401
624	PREDICTED: prominin-1-A isoform X10	94 kDa	0.0393
625	PREDICTED: patched domain-containing protein 3	97 kDa	0.038
626	PREDICTED: dynamin 1a isoform X5	99 kDa	0.0371
627	PREDICTED: solute carrier family 12 member 7 isoform X5	121 kDa	0.0304
628	PREDICTED: electrogenic sodium bicarbonate cotransporter 1 isoform X2	123 kDa	0.0299
629	PREDICTED: uncharacterized protein LOC102079324	130 kDa	0.0282
