# Supplementary material for: Purification of cone outer segment for proteomic analysis on its membrane proteins in carp retina
Source: PLoS One. 2017 Mar 14;12(3):e0173908. doi: 10.1371/journal.pone.0173908 (PMC5349680; doi:10.1371/journal.pone.0173908)
Supplement: S4 Table — Proteins in washed CIS-rich fraction were identified with LC-MS/MS analysis and are listed in descending order of emPAI values for 5 × 10^5^ cones. (PDF) [file pone.0173908.s004.pdf]

S4 Table. Identified proteins in washed CIS-rich fraction. Proteins in washed CIS-rich fraction were identified with LC-MS/MS analysis and are listed in descending order of emPAI values for 5×10^5 cones.

	Identified proteins in washed CIS-rich fraction	Molecular mass	emPAI
1	uncharacterized protein LOC100145214	33 kDa	7847
2	ATP synthase F(0) complex subunit B1, mitochondrial	31 kDa	2258
3	PREDICTED: prohibitin isoform X2	22 kDa	314.23
4	voltage-dependent anion-selective channel protein 1	31 kDa	177.58
5	PREDICTED: prohibitin	22 kDa	158.55
6	PREDICTED: creatine kinase S-type, mitochondrial isoform X2	27 kDa	85.687
7	ATP synthase subunit g, mitochondrial	11 kDa	77.77
8	PREDICTED: ATP synthase subunit alpha, mitochondrial	27 kDa	76.757
9	voltage-dependent anion-selective channel protein 2	30 kDa	72.567
10	mitochondrial 2-oxoglutarate/malate carrier protein	38 kDa	66.02
11	PREDICTED: voltage-dependent anion-selective channel protein 2	11 kDa	57.343
12	PREDICTED: creatine kinase S-type, mitochondrial isoform X2	47 kDa	47.71
13	cytochrome b-c1 complex subunit 1, mitochondrial	52 kDa	43.7
14	ATP synthase subunit d, mitochondrial	18 kDa	39.82
15	PREDICTED: uncharacterized protein LOC100707031 isoform X1	12 kDa	36.977
16	prohibitin 2a	35 kDa	33.777
17	PREDICTED: prohibitin-2	27 kDa	33.76
18	creatine kinase S-type, mitochondrial	47 kDa	32.798
19	PREDICTED: retinol dehydrogenase 8-like isoform X1	35 kDa	29.97
20	PREDICTED: NADH dehydrogenase	15 kDa	29.018
21	PREDICTED: NADH dehydrogenase	14 kDa	26.823
22	voltage-dependent anion-selective channel protein 2-like	30 kDa	26.292
23	prohibitin 2-like	27 kDa	25.383
24	PREDICTED: L-lactate dehydrogenase B-B chain isoform X3	19 kDa	24.783
25	PREDICTED: solute carrier family 25 (mitochondrial carrier; phosphate carrier), member 3a isoform X2	40 kDa	24.575
26	PREDICTED: ATP synthase subunit gamma, mitochondrial isoform X1	33 kDa	19.243
27	ATP synthase subunit alpha, mitochondrial	33 kDa	18.732
28	ES1 protein, mitochondrial precursor	31 kDa	18.641
29	serine/threonine-protein kinase MAK	11 kDa	18.142
30	NADH dehydrogenase 1 beta subcomplex subunit 4	15 kDa	17.471
31	ADP/ATP translocase 3	27 kDa	16.99
32	PREDICTED: NADH dehydrogenase	19 kDa	16.812
33	uncharacterized protein LOC541492	13 kDa	16.12
34	ATP synthase subunit O, mitochondrial	26 kDa	15.708
35	NADH dehydrogenase 1 alpha subcomplex subunit 11	16 kDa	14.404
36	PREDICTED: NADH dehydrogenase	20 kDa	14.001
37	PREDICTED: NADH dehydrogenase	25 kDa	13.02
38	PREDICTED: threonine dehydratase, mitochondrial-like isoform X2	60 kDa	12.608
39	ADP-ribosylation factor-like protein 9	26 kDa	12.599
40	PREDICTED: voltage-dependent anion-selective channel protein 3 isoform X3	36 kDa	12.562
41	mitochondrial import receptor subunit TOM70	65 kDa	12.518
42	sorting and assembly machinery component 50 homolog B	23 kDa	12.399
43	malate dehydrogenase, mitochondrial	35 kDa	12.138
44	ba1 globin, like	16 kDa	12.076
45	cytochrome c oxidase subunit 4 isoform 1, mitochondrial	20 kDa	11.65
46	cytochrome b-c1 complex subunit 1, mitochondrial	13 kDa	11.245
47	mitochondrial pyruvate carrier 1	13 kDa	10.912
48	mitochondrial import inner membrane translocase subunit tim16	14 kDa	10.712
49	calcium-binding mitochondrial carrier protein Aralar1	76 kDa	10.452
50	PREDICTED: voltage-dependent anion-selective channel protein 3 isoform X3	36 kDa	10.25
51	PREDICTED: cytochrome b-c1 complex subunit 7-like	13 kDa	9.4493
52	PREDICTED: threo-3-hydroxyaspartate ammonia-lyase-like	41 kDa	9.414
53	mitochondrial import receptor subunit TOM40 homolog	36 kDa	9.3517

54	stomatin-like protein 2, mitochondrial	39 kDa	9.2827
55	calcium-binding mitochondrial carrier protein Aralar1	60 kDa	9.1383
56	HIG1 domain family member 1A	11 kDa	8.7413
57	PREDICTED: ATP synthase subunit beta, mitochondrial-like	56 kDa	8.4217
58	arrestin-C	40 kDa	8.2907
59	mitochondrial NADH dehydrogenase (ubiquinone) 1 beta subcomplex subunit 5	22 kDa	8.091
60	PREDICTED: coiled-coil-helix-coiled-coil-helix domain-containing protein 6, mitochondrial isoform X2	29 kDa	8.0897
61	PREDICTED: phosphate carrier protein, mitochondrial-like isoform X1	34 kDa	7.9053
62	PREDICTED: NADH dehydrogenase	12 kDa	7.742
63	cytochrome c oxidase subunit IV isoform 2	20 kDa	7.485
64	PREDICTED: pyruvate dehydrogenase E1 alpha 1 isoform X1	45 kDa	7.336
65	dihydrolipoyllysine-residue acetyltransferase component of pyruvate dehydrogenase complex, mitochondrial	69 kDa	7.2543
66	sideroflexin-3	36 kDa	7.1363
67	stomatin-like protein 2, mitochondrial	39 kDa	7.1153
68	PREDICTED: NADH dehydrogenase	17 kDa	7.0023
69	NADH dehydrogenase	41 kDa	6.9657
70	cytochrome c oxidase subunit VIIa polypeptide 3	16 kDa	6.909
71	PREDICTED: voltage-dependent anion-selective channel protein 2	11 kDa	6.7737
72	plasminogen receptor (KT)	20 kDa	6.554
73	NADH dehydrogenase 1 beta subcomplex subunit 6	15 kDa	6.357
74	NADH dehydrogenase (ubiquinone) 1 subunit c2	13 kDa	6.0537
75	sorting and assembly machinery component 50 homolog A	52 kDa	6.0447
76	aspartate aminotransferase 2a	48 kDa	5.9803
77	cytochrome c-1	36 kDa	5.9553
78	mitochondrial chaperone BCS1	48 kDa	5.938
79	cytochrome c oxidase subunit 6A1, mitochondrial	13 kDa	5.9023
80	NADH dehydrogenase	24 kDa	5.8123
81	NADH dehydrogenase	31 kDa	5.646
82	CDGSH iron sulfur domain 1	12 kDa	5.5593
83	PREDICTED: mitochondrial pyruvate carrier 2	14 kDa	5.542
84	cytochrome c oxidase subunit 4 isoform 1, mitochondrial	20 kDa	5.5203
85	mitochondrial import receptor subunit TOM40 homolog	36 kDa	5.4667
86	histone 1, H4, like	12 kDa	5.142
87	PREDICTED: calcium-binding mitochondrial carrier protein Aralar2 isoform X1	48 kDa	4.8943
88	PREDICTED: calcium-binding mitochondrial carrier protein Aralar2 isoform X1	15 kDa	4.7787
89	cytochrome b-c1 complex subunit 2, mitochondrial	50 kDa	4.7677
90	trifunctional enzyme subunit beta, mitochondrial	50 kDa	4.7373
91	PREDICTED: pyruvate dehydrogenase E1 component subunit alpha, somatic form, mitochondrial isoform X1	25 kDa	4.669
92	alpha globin-like	15 kDa	4.5913
93	isocitrate dehydrogenase	50 kDa	4.306
94	NADH dehydrogenase	53 kDa	4.2797
95	reticulon-4-interacting protein 1 homolog, mitochondrial	42 kDa	4.219
96	retinol dehydrogenase 13	37 kDa	4.108
97	NADH-cytochrome b5 reductase 1	28 kDa	3.9437
98	apolipoprotein O	24 kDa	3.9143
99	PREDICTED: ubiquinol-cytochrome c reductase core protein II isoform X1	49 kDa	3.7547
100	protein NipSnap homolog 2	33 kDa	3.6203
101	inactive hydroxysteroid dehydrogenase-like protein 1	35 kDa	3.568
102	NADH dehydrogenase 1 alpha subcomplex subunit 6	16 kDa	3.4727
103	LETM1 and EF-hand domain-containing protein 1, mitochondrial	86 kDa	3.3119
104	aspartate aminotransferase 2	43 kDa	3.2839
105	voltage-dependent anion-selective channel protein 2	20 kDa	2.9212
106	PREDICTED: mitochondrial glutamate carrier 1	34 kDa	2.9134
107	translocase of outer mitochondrial membrane 40 homolog, like	35 kDa	2.8674
108	mitochondrial dicarboxylate carrier	32 kDa	2.842
109	PREDICTED: ubiquinol-cytochrome c reductase core protein II isoform X1	40 kDa	2.8386
110	ADP-ribosylation factor-like protein 9	25 kDa	2.8341

111	NADH dehydrogenase	58 kDa	2.8289
112	NADH-ubiquinone oxidoreductase 75 kDa subunit, mitochondrial	80 kDa	2.7294
113	succinyl-CoA ligase	51 kDa	2.6793
114	translocase of outer mitochondrial membrane 40 homolog, like	36 kDa	2.6352
115	NADH dehydrogenase	13 kDa	2.5723
116	NADH dehydrogenase (ubiquinone) 1 subunit c2	13 kDa	2.5723
117	isocitrate dehydrogenase	43 kDa	2.5709
118	PREDICTED: NADH dehydrogenase	43 kDa	2.5394
119	PREDICTED: coiled-coil domain-containing protein 136-like isoform X1	53 kDa	2.5324
120	PREDICTED: isocitrate dehydrogenase	44 kDa	2.4932
121	PREDICTED: mitochondrial fission process protein 1-like	18 kDa	2.4453
122	cytochrome c oxidase subunit Vaa	18 kDa	2.3777
123	28S ribosomal protein S36, mitochondrial	12 kDa	2.2468
124	heat shock cognate 71 kDa protein	42 kDa	2.2293
125	ATPase family AAA domain-containing protein 3	69 kDa	2.2163
126	3-hydroxyacyl-CoA dehydrogenase type-2	28 kDa	2.1361
127	PREDICTED: uncharacterized protein LOC556653	23 kDa	2.1319
128	PREDICTED: hexokinase-1-like	14 kDa	2.1305
129	uncharacterized protein C18orf19 homolog B	32 kDa	2.1159
130	PREDICTED: NADH dehydrogenase	21 kDa	2.0971
131	NADH dehydrogenase	12 kDa	2.0714
132	cytochrome c oxidase subunit Vaa	16 kDa	2.0471
133	guanine nucleotide-binding protein G(t) subunit alpha-2	40 kDa	2.0231
134	enoyl-CoA hydratase, mitochondrial	31 kDa	1.9767
135	OCIA domain-containing protein 1	30 kDa	1.9289
136	reticulon-4	22 kDa	1.9169
137	pyruvate dehydrogenase E1 component subunit beta, mitochondrial	39 kDa	1.9136
138	methylmalonate-semialdehyde dehydrogenase	61 kDa	1.8848
139	NADH dehydrogenase	17 kDa	1.822
140	retinol dehydrogenase-like	36 kDa	1.8206
141	L-lactate dehydrogenase B-A chain	36 kDa	1.8206
142	sideroflexin-4	35 kDa	1.8005
143	PREDICTED: presenilins-associated rhomboid-like protein, mitochondrial	41 kDa	1.7799
144	coiled-coil-helix-coiled-coil-helix domain-containing protein 3, mitochondrial	37 kDa	1.7621
145	ATPase family AAA domain-containing protein 3	69 kDa	1.7604
146	PREDICTED: complex I assembly factor TIMMDC1, mitochondrial isoform X1	33 kDa	1.7283
147	PREDICTED: NADH dehydrogenase	21 kDa	1.7263
148	PREDICTED: L-lactate dehydrogenase B-B chain isoform X4	19 kDa	1.7052
149	uncharacterized protein LOC100135302	20 kDa	1.6653
150	putative tubulin beta chain variant 1	20 kDa	1.6459
151	mitochondrial inner membrane protein	83 kDa	1.6335
152	PREDICTED: LOW QUALITY PROTEIN: actin, gamma 1	40 kDa	1.6204
153	creatine kinase U-type, mitochondrial	22 kDa	1.619
154	isocitrate dehydrogenase	43 kDa	1.6078
155	PREDICTED: calcium-binding mitochondrial carrier protein SCaMC-2-B isoform X2	49 kDa	1.5994
156	PREDICTED: vesicle-associated membrane protein-associated protein A-like	26 kDa	1.5929
157	PREDICTED: vesicle-associated membrane protein-associated protein A-like	26 kDa	1.5929
158	thioredoxin-dependent peroxide reductase, mitochondrial	28 kDa	1.5892
159	mitochondrial import inner membrane translocase subunit Tim17-B	18 kDa	1.5758
160	protein QIL1	12 kDa	1.5631
161	PREDICTED: nuclease EXOG, mitochondrial	39 kDa	1.5583
162	transmembrane protein 256 precursor	12 kDa	1.5345
163	PREDICTED: very long-chain specific acyl-CoA dehydrogenase, mitochondrial isoform X1	71 kDa	1.5216
164	PREDICTED: dehydrogenase/reductase SDR family member 7B isoform X1	31 kDa	1.5171
165	glutaryl-CoA dehydrogenase a	18 kDa	1.5004
166	isocitrate dehydrogenase	40 kDa	1.4881
167	ATP-binding cassette sub-family B member 8, mitochondrial	77 kDa	1.4777
168	sorting and assembly machinery component 50 homolog B	27 kDa	1.464
169	NAD(P) transhydrogenase, mitochondrial	114 kDa	1.4534

170	PREDICTED: calcium/calmodulin-dependent protein kinase type II delta 1 chain isoform X3	28 kDa	1.4404
171	dihydrolipoyl dehydrogenase, mitochondrial	54 kDa	1.4384
172	metaxin 1	36 kDa	1.4365
173	PREDICTED: LOW QUALITY PROTEIN: long-chain-fatty-acid--CoA ligase 6	50 kDa	1.4107
174	PREDICTED: mitochondrial fission factor-like isoform X4	24 kDa	1.3838
175	COX16 cytochrome c oxidase assembly homolog	13 kDa	1.3807
176	mitochondrial carrier homolog 2	31 kDa	1.3454
177	PREDICTED: tubulin beta-4B chain-like	31 kDa	1.3266
178	reticulon-4	22 kDa	1.3162
179	PREDICTED: choline dehydrogenase, mitochondrial	25 kDa	1.2768
180	ATPase, Na+/K+ transporting, beta 2b polypeptide	34 kDa	1.2638
181	[3-methyl-2-oxobutanoate dehydrogenase	48 kDa	1.2509
182	PREDICTED: retinol dehydrogenase 12-like	34 kDa	1.2403
183	AFG3-like protein 2	89 kDa	1.2328
184	cytochrome b-c1 complex subunit Rieske, mitochondrial	30 kDa	1.2288
185	PREDICTED: probable UDP-sugar transporter protein SLC35A4 isoform X1	11 kDa	1.2058
186	PREDICTED: presenilins-associated rhomboid-like protein, mitochondrial-like isoform X2	11 kDa	1.2058
187	elongation factor Tu, mitochondrial	49 kDa	1.1921
188	erlin-2 precursor	40 kDa	1.1837
189	ADP-ribosylation factor-like protein 6	21 kDa	1.1684
190	acetyl-CoA acetyltransferase, mitochondrial precursor	48 kDa	1.1577
191	PREDICTED: mitochondrial import inner membrane translocase subunit Tim22	21 kDa	1.1569
192	ras-related protein Rab-2A	24 kDa	1.1561
193	solute carrier family 25 (mitochondrial carrier: glutamate), member 22	36 kDa	1.1538
194	PREDICTED: mitochondrial fission factor-like isoform X3	26 kDa	1.1463
195	mitochondrial import receptor subunit TOM20 homolog B	16 kDa	1.1448
196	PREDICTED: glutaryl-CoA dehydrogenase, mitochondrial-like	49 kDa	1.1278
197	zinc transporter 9	64 kDa	1.1104
198	citrate synthase, mitochondrial precursor	52 kDa	1.0972
199	PREDICTED: stress-70 protein, mitochondrial-like, partial	17 kDa	1.0902
200	PREDICTED: tubulin alpha chain-like	12 kDa	1.0861
201	mitochondrial trifunctional protein, alpha subunit	83 kDa	1.071
202	transmembrane protein 256 precursor	12 kDa	1.0682
203	PREDICTED: coiled-coil domain-containing protein 136-like isoform X1	30 kDa	1.0538
204	PREDICTED: kynurenine/alpha-aminoadipate aminotransferase, mitochondrial isoform X1	48 kDa	1.0501
205	PREDICTED: ras-related protein Rab-1A-like isoform X1	22 kDa	1.0436
206	PREDICTED: nucleoside diphosphate kinase B isoform X1	17 kDa	1.0402
207	ras-related protein Rab-1B	22 kDa	1.0343
208	succinate dehydrogenase	61 kDa	1.0337
209	mitochondrial import inner membrane translocase subunit Tim17-A	17 kDa	1.0283
210	regulator of microtubule dynamics protein 3	51 kDa	1.0172
211	PREDICTED: protein FAM162B isoform X1	17 kDa	1.0167
212	PREDICTED: peroxiredoxin-5, mitochondrial-like	20 kDa	1.0114
213	succinate dehydrogenase	73 kDa	1.0022
214	pyrroline-5-carboxylate reductase 1a	34 kDa	0.9856
215	uncharacterized protein LOC100135302	23 kDa	0.9732
216	histone H2AX	15 kDa	0.972
217	dnaJ homolog subfamily C member 11	64 kDa	0.9565
218	methylglutaconyl-CoA hydratase, mitochondrial	35 kDa	0.9354
219	PREDICTED: FAS-associated factor 2-like isoform X1	54 kDa	0.9249
220	dihydrolipoyllysine-residue succinyltransferase component of 2-oxoglutarate dehydrogenase complex, mitochondrial	51 kDa	0.9224
221	PREDICTED: dephospho-CoA kinase domain-containing protein-like isoform X2	24 kDa	0.9039
222	PREDICTED: LOW QUALITY PROTEIN: long-chain-fatty-acid--CoA ligase 6	75 kDa	0.891
223	ubiquinone biosynthesis monooxygenase COQ6	52 kDa	0.8882
224	metaxin 1a	36 kDa	0.8661
225	PREDICTED: tubulin alpha chain	45 kDa	0.8658

226	NADH dehydrogenase (ubiquinone) Fe-S protein 8b	22 kDa	0.8573
227	hexokinase-1	54 kDa	0.8501
228	ATP synthase F(0) complex subunit C3, mitochondrial	14 kDa	0.8416
229	PREDICTED: transmembrane protein 126A isoform X1	22 kDa	0.8358
230	histone 2, H2a	14 kDa	0.8304
231	uncharacterized protein C18orf19 homolog A	29 kDa	0.8156
232	histone 2, H2a	14 kDa	0.8087
233	hexokinase-1	11 kDa	0.8001
234	PREDICTED: apoptosis-inducing factor 1, mitochondrial isoform X3	68 kDa	0.7994
235	regulator of microtubule dynamics protein 3	35 kDa	0.7984
236	cysteine desulfurase, mitochondrial	50 kDa	0.7902
237	hexokinase-1	71 kDa	0.7863
238	protein disulfide-isomerase TMX3 precursor	47 kDa	0.7839
239	PREDICTED: coiled-coil-helix-coiled-coil-helix domain-containing protein 2, mitochondrial	17 kDa	0.7803
240	PREDICTED: tubulin alpha-1C chain	45 kDa	0.7769
241	PREDICTED: mitochondrial import inner membrane translocase subunit Tim21	27 kDa	0.7623
242	protein NDRG1 isoform 1	42 kDa	0.7588
243	ubiquinone biosynthesis monooxygenase COQ6	52 kDa	0.7533
244	PREDICTED: uncharacterized protein LOC101885612	11 kDa	0.7513
245	cytochrome c	11 kDa	0.7513
246	optic atrophy 3 protein homolog	18 kDa	0.7487
247	vesicle-associated membrane protein-associated protein A	30 kDa	0.7465
248	PREDICTED: gap junction delta-2 protein-like	21 kDa	0.7351
249	PREDICTED: dnaJ homolog subfamily C member 11	65 kDa	0.7321
250	PREDICTED: protein SCO1 homolog, mitochondrial	34 kDa	0.7261
251	PREDICTED: hydroxyacylglutathione hydrolase, mitochondrial isoform X1	34 kDa	0.7261
252	uncharacterized protein LOC100127838	18 kDa	0.7193
253	PREDICTED: ATP-binding cassette sub-family B member 7, mitochondrial	82 kDa	0.716
254	L-2-hydroxyglutarate dehydrogenase, mitochondrial	31 kDa	0.7117
255	PREDICTED: uncharacterized protein LOC571872 isoform X1	12 kDa	0.7078
256	PREDICTED: mitochondrial fission factor homolog B isoform X1	34 kDa	0.707
257	stress-70 protein, mitochondrial	73 kDa	0.7019
258	mitochondrial trifunctional protein, alpha subunit	38 kDa	0.6997
259	PREDICTED: protein MGARP isoform X2	41 kDa	0.6936
260	mitochondrial import inner membrane translocase subunit Tim23	22 kDa	0.6933
261	PREDICTED: uncharacterized protein LOC556653	35 kDa	0.6888
262	mitochondrial ubiquitin ligase activator of nfkb 1-A	12 kDa	0.6878
263	COX16 cytochrome c oxidase assembly homolog	12 kDa	0.6878
264	phosphatidate cytidylyltransferase, mitochondrial precursor	12 kDa	0.6878
265	uncharacterized protein LOC100145220	29 kDa	0.6777
266	PREDICTED: apoptosis-inducing factor 1, mitochondrial isoform X1	48 kDa	0.6765
267	PREDICTED: mitochondrial import inner membrane translocase subunit Tim23	22 kDa	0.6713
268	PREDICTED: pyrroline-5-carboxylate reductase 2-like	16 kDa	0.6675
269	calcium/calmodulin-dependent protein kinase type II delta 1 chain isoform 1	56 kDa	0.6639
270	aconitate hydratase, mitochondrial	86 kDa	0.6634
271	mitochondrial ATP synthase subunit f	13 kDa	0.6508
272	PREDICTED: choline dehydrogenase, mitochondrial	20 kDa	0.643
273	translocase of outer mitochondrial membrane 20 homolog a	16 kDa	0.6392
274	PREDICTED: cytochrome b-c1 complex subunit Rieske, mitochondrial-like	26 kDa	0.6391
275	PREDICTED: [Pyruvate dehydrogenase (acetyl-transferring)] kinase isozyme 1, mitochondrial	47 kDa	0.6342
276	arrestin-C	33 kDa	0.6335
277	PREDICTED: regulator of G-protein signaling 9-binding protein-like	27 kDa	0.6307
278	uncharacterized protein LOC541537	27 kDa	0.6307
279	PREDICTED: ATP-binding cassette sub-family B member 10, mitochondrial	30 kDa	0.6231
280	acylglycerol kinase, mitochondrial precursor	48 kDa	0.6225
281	delta-1-pyrroline-5-carboxylate dehydrogenase, mitochondrial precursor	62 kDa	0.6219
282	PREDICTED: ATPase, Na+/K+ transporting, beta 2b polypeptide isoform X1	24 kDa	0.6172
283	PREDICTED: glutaminase a isoform X1	66 kDa	0.6105

284	PREDICTED: mitochondrial pyruvate carrier 2-like	17 kDa	0.6069
285	PREDICTED: uncharacterized aarF domain-containing protein kinase 5 isoform X1	42 kDa	0.6033
286	PREDICTED: sterol 26-hydroxylase, mitochondrial	20 kDa	0.6001
287	60S ribosomal protein L15	24 kDa	0.5996
288	abhydrolase domain-containing protein 16A	38 kDa	0.5985
289	PREDICTED: growth hormone-inducible transmembrane protein	35 kDa	0.5898
290	uncharacterized protein C2orf47 homolog, mitochondrial	28 kDa	0.5809
291	PREDICTED: tubulin alpha chain-like	50 kDa	0.5754
292	PREDICTED: ATP-dependent zinc metalloprotease YME1L1 isoform X1	79 kDa	0.5752
293	60 kDa heat shock protein, mitochondrial	28 kDa	0.5705
294	3-ketoacyl-CoA thiolase, mitochondrial	43 kDa	0.5674
295	carnitine O-palmitoyltransferase 2, mitochondrial	18 kDa	0.5611
296	very-long-chain enoyl-CoA reductase	36 kDa	0.5593
297	PREDICTED: growth hormone-inducible transmembrane protein	36 kDa	0.5566
298	mitochondrial Rho GTPase 2	70 kDa	0.5565
299	PREDICTED: actin, aortic smooth muscle	18 kDa	0.5559
300	ubiquinol-cytochrome c reductase complex assembly factor 1	33 kDa	0.5468
301	vesicle-associated membrane protein-associated protein A	30 kDa	0.5381
302	PREDICTED: ornithine aminotransferase, mitochondrial	49 kDa	0.5359
303	ras-related protein Rab-5C	37 kDa	0.534
304	mitochondrial carnitine/acylcarnitine carrier protein CACL	34 kDa	0.5278
305	NADH dehydrogenase	27 kDa	0.5141
306	PREDICTED: succinate dehydrogenase cytochrome b560 subunit, mitochondrial isoform X1	19 kDa	0.5124
307	phosphatidate cytidylyltransferase, mitochondrial precursor	15 kDa	0.511
308	PREDICTED: transmembrane protein C9orf123 homolog	11 kDa	0.5014
309	coiled-coil domain-containing protein 51	47 kDa	0.4994
310	40S ribosomal protein S25	15 kDa	0.4948
311	E3 ubiquitin-protein ligase MARCH5	31 kDa	0.4904
312	mitochondrial import inner membrane translocase subunit TIM44	52 kDa	0.4864
313	PREDICTED: ras-related protein Rab-7a isoform X1	24 kDa	0.4851
314	transmembrane protein 65	24 kDa	0.4851
315	traB domain-containing protein	40 kDa	0.4835
316	carnitine O-palmitoyltransferase 2, mitochondrial	20 kDa	0.4829
317	RAB1A, member RAS oncogene family	28 kDa	0.4809
318	solute carrier family 25 member 47-B	12 kDa	0.4679
319	serine hydroxymethyltransferase, mitochondrial	57 kDa	0.4676
320	protein QIL1	12 kDa	0.4617
321	nucleoside diphosphate kinase A	20 kDa	0.46
322	sodium/potassium-transporting ATPase subunit alpha-3	113 kDa	0.46
323	PREDICTED: ras-related protein Rab-11B	25 kDa	0.4566
324	PREDICTED: FUN14 domain-containing protein 2 isoform X1	16 kDa	0.4517
325	PREDICTED: mitochondrial ubiquitin ligase activator of nfkb 1-A	33 kDa	0.4502
326	PREDICTED: saccharopine dehydrogenase-like oxidoreductase-like	12 kDa	0.4498
327	LIM domain and actin-binding protein 1	20 kDa	0.4494
328	60 kDa heat shock protein, mitochondrial	29 kDa	0.4492
329	synaptojanin-2-binding protein	16 kDa	0.4474
330	39S ribosomal protein L43, mitochondrial	16 kDa	0.4474
331	PREDICTED: mitochondrial dicarboxylate carrier	29 kDa	0.4467
332	PREDICTED: solute carrier family 25 member 51-like	34 kDa	0.4416
333	acyl-CoA dehydrogenase family member 9, mitochondrial	69 kDa	0.4368
334	PREDICTED: heat shock cognate 71 kDa protein, partial	30 kDa	0.4256
335	PREDICTED: sarcolemmal membrane-associated protein isoform X4	92 kDa	0.4246
336	mitochondrial import inner membrane translocase subunit TIM14	13 kDa	0.4225
337	PREDICTED: magnesium transporter MRS2 homolog, mitochondrial isoform X1	48 kDa	0.4222
338	cytosolic 5'-nucleotidase 3	39 kDa	0.4201
339	outer dense fiber of sperm tails 2b	22 kDa	0.4201
340	60S ribosomal protein L18	22 kDa	0.417
341	uncharacterized protein LOC556781	13 kDa	0.4125
342	mitochondrial carnitine/acylcarnitine carrier protein	22 kDa	0.4111

343	metaxin-2	31 kDa	0.4105
344	PREDICTED: LETM1 domain-containing protein LETM2, mitochondrial	54 kDa	0.4099
345	PREDICTED: ADP-dependent glucokinase isoform X2	58 kDa	0.4098
346	heat shock protein 75 kDa, mitochondrial	82 kDa	0.4056
347	PREDICTED: mitochondrial glutamate carrier 1-like	22 kDa	0.4053
348	solute carrier family 25 member 46	45 kDa	0.4015
349	epimerase family protein SDR39U1	36 kDa	0.3997
350	PREDICTED: malate dehydrogenase 1Aa, NAD (soluble) isoform X1	36 kDa	0.3979
351	PREDICTED: 40S ribosomal protein S9-like	22 kDa	0.3969
352	surfeit locus protein 1	32 kDa	0.3945
353	PREDICTED: succinate dehydrogenase	32 kDa	0.3945
354	PREDICTED: multiple PDZ domain protein isoform X1	93 kDa	0.3942
355	PREDICTED: ras-related protein Rab-10	23 kDa	0.3915
356	hydroxysteroid dehydrogenase-like protein 2	51 kDa	0.391
357	COX15 homolog	51 kDa	0.3898
358	mpv17-like protein 2	23 kDa	0.3863
359	PREDICTED: uncharacterized protein LOC571872 isoform X1	32 kDa	0.385
360	1-acyl-sn-glycerol-3-phosphate acyltransferase epsilon	42 kDa	0.3843
361	PREDICTED: 60S ribosomal protein L23a	18 kDa	0.3842
362	very-long-chain (3R)-3-hydroxyacyl-CoA dehydratase 2	28 kDa	0.3834
363	delta-1-pyrroline-5-carboxylate synthase	85 kDa	0.381
364	PREDICTED: mitochondrial Rho GTPase 1 isoform X4	71 kDa	0.38
365	mitochondrial dynamics protein MID51	52 kDa	0.377
366	fatty aldehyde dehydrogenase	28 kDa	0.3751
367	PREDICTED: LOW QUALITY PROTEIN: 40S ribosomal protein S13-like	19 kDa	0.3748
368	PREDICTED: 40S ribosomal protein S14 isoform X1	19 kDa	0.3717
369	RAB5A, member RAS oncogene family, a	24 kDa	0.3713
370	ras-related protein Rab-1B	19 kDa	0.3687
371	PREDICTED: paraplegin	92 kDa	0.3682
372	PREDICTED: 40S ribosomal protein S8	24 kDa	0.3596
373	alpha/beta hydrolase domain-containing protein 11	34 kDa	0.3592
374	chaperone activity of bc1 complex-like, mitochondrial	70 kDa	0.3546
375	coiled-coil domain-containing protein 90B, mitochondrial	29 kDa	0.3538
376	PREDICTED: tubulin beta-2B chain-like isoform 1	55 kDa	0.3527
377	PREDICTED: reticulon-3-B-like isoform X2	25 kDa	0.3487
378	dynamin-like 120 kDa protein, mitochondrial precursor	112 kDa	0.3475
379	uncharacterized protein LOC100135257	35 kDa	0.3467
380	calmegin precursor	66 kDa	0.3453
381	electron transfer flavoprotein subunit alpha, mitochondrial	35 kDa	0.3437
382	PREDICTED: mitochondrial 2-oxodicarboxylate carrier isoform X2	15 kDa	0.3427
383	retinol dehydrogenase 8a	35 kDa	0.3408
384	NADH dehydrogenase 1 beta subcomplex subunit 10	20 kDa	0.3362
385	PREDICTED: 40S ribosomal protein S15a isoform X1	15 kDa	0.3359
386	dolichyl-diphosphooligosaccharide--protein glycosyltransferase subunit 1 precursor	67 kDa	0.3337
387	PREDICTED: regulator of microtubule dynamics protein 2 isoform X1	47 kDa	0.3333
388	acyl-CoA synthetase long-chain family member 3b	79 kDa	0.3283
389	succinate dehydrogenase	32 kDa	0.3221
390	arginase-1	37 kDa	0.3216
391	cytosol aminopeptidase	21 kDa	0.3174
392	PREDICTED: succinyl-CoA ligase	16 kDa	0.3172
393	PREDICTED: LOW QUALITY PROTEIN: metalloendopeptidase OMA1, mitochondrial	16 kDa	0.3172
394	transmembrane protein 11, mitochondrial	27 kDa	0.3158
395	ATP synthase subunit s, mitochondrial	27 kDa	0.3123
396	calcium-binding mitochondrial carrier protein SCaMC-1	16 kDa	0.3114
397	retinol dehydrogenase 14	32 kDa	0.3104
398	peptidyl-prolyl cis-trans isomerase FKBP8	44 kDa	0.3101
399	PRA1 family protein 3	22 kDa	0.3067
400	PREDICTED: sterol 26-hydroxylase, mitochondrial	27 kDa	0.3056
401	mitochondrial import inner membrane translocase subunit TIM50 precursor	44 kDa	0.3049

402	PREDICTED: NADH dehydrogenase (ubiquinone) complex I, assembly factor 6	33 kDa	0.3048
403	ubiquinone biosynthesis protein COQ7 homolog	22 kDa	0.2986
404	rho-related gtp-binding protein rhoc	22 kDa	0.2986
405	ATPase family AAA domain-containing protein 1-B	22 kDa	0.2986
406	carnitine O-palmitoyltransferase 2, mitochondrial	22 kDa	0.2986
407	PREDICTED: enoyl-CoA delta isomerase 1, mitochondrial	33 kDa	0.2981
408	L-lactate dehydrogenase A chain	39 kDa	0.2963
409	G-protein-coupled receptor kinase 7A	62 kDa	0.2953
410	cytochrome c-type heme lyase	34 kDa	0.2943
411	PREDICTED: 10 kDa heat shock protein, mitochondrial isoform X2	11 kDa	0.2923
412	uncharacterized protein LOC553782	11 kDa	0.2923
413	uncharacterized protein LOC100127828	34 kDa	0.288
414	solute carrier family 25 member 33	34 kDa	0.2868
415	mitochondrial ubiquitin ligase activator of nfkb 1-A	23 kDa	0.2854
416	PREDICTED: pyruvate dehydrogenase kinase, isozyme 3 isoform X2	46 kDa	0.2848
417	DDRGK domain-containing protein 1 precursor	35 kDa	0.2844
418	trifunctional enzyme subunit alpha, mitochondrial	47 kDa	0.2839
419	renalase	41 kDa	0.2816
420	phosphatidylglycerophosphatase and protein-tyrosine phosphatase 1	11 kDa	0.2813
421	40S ribosomal protein S7	17 kDa	0.2805
422	40S ribosomal protein S4, X isoform	29 kDa	0.2799
423	uncharacterized protein LOC449549	23 kDa	0.2784
424	phosphatidate cytidylyltransferase, mitochondrial precursor	11 kDa	0.2779
425	uncharacterized protein LOC556781	23 kDa	0.2767
426	PREDICTED: 60S ribosomal protein L9 isoform X2	17 kDa	0.2759
427	band 3 anion transport protein	11 kDa	0.2745
428	PREDICTED: pyruvate dehydrogenase protein X component, mitochondrial	54 kDa	0.2734
429	NAD-dependent protein deacetylase sirtuin-3, mitochondrial	18 kDa	0.2715
430	lipoamide acyltransferase component of branched-chain alpha-keto acid dehydrogenase complex, mitochondrial	54 kDa	0.2712
431	60S ribosomal protein L14	18 kDa	0.2694
432	40S ribosomal protein S19	18 kDa	0.2694
433	60S ribosomal protein L7a	30 kDa	0.2692
434	PREDICTED: glycerol kinase-like isoform X4	30 kDa	0.2692
435	glycerophosphodiester phosphodiesterase domain-containing protein 1	36 kDa	0.2686
436	mitofusin-2	80 kDa	0.268
437	PREDICTED: syntaxin-12 isoform X1	30 kDa	0.2679
438	60S ribosomal protein L4	43 kDa	0.2673
439	PREDICTED: ferrochelatase, mitochondrial isoform X1	43 kDa	0.2673
440	60S ribosomal protein L24	18 kDa	0.2672
441	protein THEM6 precursor	24 kDa	0.2668
442	solute carrier family 25 member 47-B	24 kDa	0.2668
443	PREDICTED: calnexin isoform X1	67 kDa	0.2661
444	protein THEM6 precursor	24 kDa	0.2653
445	60S ribosomal protein L27a	18 kDa	0.2651
446	guanine nucleotide binding protein (G protein), beta polypeptide 3b	37 kDa	0.2644
447	probable 28S ribosomal protein S10, mitochondrial	12 kDa	0.2617
448	electron transfer flavoprotein-ubiquinone oxidoreductase, mitochondrial	69 kDa	0.2605
449	G-protein-coupled receptor kinase 7A	62 kDa	0.26
450	phosphatidylglycerophosphatase and protein-tyrosine phosphatase 1	12 kDa	0.2587
451	PREDICTED: ATP-dependent Clp protease ATP-binding subunit clpX-like, mitochondrial	44 kDa	0.2577
452	40S ribosomal protein S18	18 kDa	0.257
453	40S ribosomal protein S5	25 kDa	0.2562
454	peptidyl-prolyl cis-trans isomerase FKBP8	44 kDa	0.256
455	eukaryotic translation elongation factor 1 alpha 1-like	50 kDa	0.2557
456	PREDICTED: epoxide hydrolase 1, partial	38 kDa	0.2554
457	PREDICTED: von Willebrand factor A domain-containing protein 1	25 kDa	0.2548
458	NADH dehydrogenase (ubiquinone) 1, alpha/beta subcomplex 1-like	19 kDa	0.2532
459	uncharacterized protein LOC723999	19 kDa	0.2532

460	PREDICTED: vesicle-associated membrane protein 2-like	12 kDa	0.2529
461	long-chain fatty acid transport protein 4	25 kDa	0.2519
462	dynamin-1-like protein	77 kDa	0.2503
463	PREDICTED: 60S ribosomal protein L36	12 kDa	0.2501
464	PREDICTED: 60 kDa heat shock protein, mitochondrial-like, partial	12 kDa	0.2501
465	2-methoxy-6-polyprenyl-1,4-benzoquinol methylase, mitochondrial precursor	38 kDa	0.2497
466	PREDICTED: lipoamide acyltransferase component of branched-chain alpha-keto acid dehydrogenase complex, mitochondrial-like	45 kDa	0.2495
467	NADH dehydrogenase	19 kDa	0.2494
468	PRELI domain containing 1b	25 kDa	0.2491
469	green-sensitive opsin-4	39 kDa	0.2479
470	mimitin, mitochondrial	19 kDa	0.2476
471	solute carrier family 25 member 40	39 kDa	0.247
472	PREDICTED: glutaminase kidney isoform, mitochondrial isoform X3	26 kDa	0.245
473	PREDICTED: NADPH:adrenodoxin oxidoreductase, mitochondrial	12 kDa	0.2446
474	erlin-1 precursor	39 kDa	0.2434
475	bcl-2-like protein 13	52 kDa	0.2432
476	isovaleryl-CoA dehydrogenase, mitochondrial	46 kDa	0.2426
477	uncharacterized protein LOC100127834	33 kDa	0.2425
478	PREDICTED: NCK-interacting protein with SH3 domain-like	19 kDa	0.2423
479	PREDICTED: UPF0562 protein C7orf55 homolog	13 kDa	0.242
480	pancreatic progenitor cell differentiation and proliferation factor A	13 kDa	0.242
481	PREDICTED: OCIA domain-containing protein 1 isoform X1	26 kDa	0.2398
482	PREDICTED: dnaJ homolog subfamily C member 30-like isoform X1	26 kDa	0.2385
483	probable glutamate--tRNA ligase, mitochondrial precursor	40 kDa	0.2374
484	PREDICTED: apoptosis-inducing factor 1, mitochondrial isoform X1	13 kDa	0.2369
485	PREDICTED: red-sensitive opsin-1 isoform X1	41 kDa	0.2325
486	PREDICTED: NADH dehydrogenase	20 kDa	0.2306
487	PREDICTED: protein phosphatase 1K, mitochondrial isoform X1	41 kDa	0.2293
488	PREDICTED: 40S ribosomal protein S2	20 kDa	0.2291
489	PREDICTED: cytochrome c oxidase assembly factor 1 homolog isoform X3	20 kDa	0.2291
490	PREDICTED: transmembrane emp24 domain-containing protein 9 isoform X1	27 kDa	0.2288
491	NADH dehydrogenase	48 kDa	0.2278
492	PREDICTED: epoxide hydrolase 1-like, partial	20 kDa	0.2245
493	40S ribosomal protein S10	20 kDa	0.2245
494	carnitine O-acetyltransferase b	70 kDa	0.2237
495	acyl-CoA dehydrogenase-like	49 kDa	0.2232
496	PREDICTED: proteoglycan 4-like isoform X1	13 kDa	0.2228
497	PREDICTED: phosphatidylserine decarboxylase proenzyme-like isoform X2	42 kDa	0.2225
498	mitochondrial folate transporter/carrier	35 kDa	0.2205
499	ras-related protein Rap-1b precursor	21 kDa	0.22
500	probable 2-oxoglutarate dehydrogenase E1 component DHKTD1, mitochondrial	107 kDa	0.2195
501	PREDICTED: T-cell activation inhibitor, mitochondrial	57 kDa	0.2183
502	ras-related protein Rap-1b-like	21 kDa	0.2172
503	60S ribosomal protein L7	29 kDa	0.2145
504	PREDICTED: coiled-coil domain containing 127a isoform X1	36 kDa	0.2138
505	uncharacterized protein LOC100302470 precursor	21 kDa	0.2131
506	NADH dehydrogenase	21 kDa	0.2131
507	60S ribosomal protein L30	14 kDa	0.2122
508	S-adenosylmethionine mitochondrial carrier protein	29 kDa	0.2095
509	protoporphyrinogen oxidase	52 kDa	0.2089
510	ADP-ribosylation factor-like 3, like 1	22 kDa	0.2078
511	PREDICTED: mitochondrial calcium uniporter regulator 1	22 kDa	0.2078
512	PREDICTED: choline dehydrogenase, mitochondrial	29 kDa	0.2066
513	recoverin-like	22 kDa	0.2065
514	28S ribosomal protein S23, mitochondrial	22 kDa	0.2065
515	28S ribosomal protein S5, mitochondrial precursor	45 kDa	0.2054
516	calcium-binding mitochondrial carrier protein SCaMC-2-A	52 kDa	0.2051
517	PREDICTED: uncharacterized aarF domain-containing protein kinase 2	14 kDa	0.2044
518	PREDICTED: cytochrome c oxidase subunit 5B, mitochondrial-like	14 kDa	0.2044

519	PREDICTED: transmembrane protein 70, mitochondrial	30 kDa	0.2028
520	PREDICTED: 60S ribosomal protein L6	30 kDa	0.2019
521	CAAX prenyl protease 1 homolog	53 kDa	0.2003
522	ras-related protein Rab-18	23 kDa	0.1979
523	synaptosomal-associated protein 25-B	23 kDa	0.1979
524	PREDICTED: transmembrane emp24 domain-containing protein 2	23 kDa	0.1968
525	uncharacterized protein LOC492355	23 kDa	0.1945
526	methyltransferase like 7A precursor	31 kDa	0.194
527	saccharopine dehydrogenase b	47 kDa	0.1935
528	opsin-1, short-wave-sensitive 2	39 kDa	0.1924
529	transcription factor A, mitochondrial	31 kDa	0.1907
530	dnaJ homolog subfamily C member 2	48 kDa	0.1902
531	78 kDa glucose-regulated protein precursor	72 kDa	0.1892
532	elongation factor 1-alpha	48 kDa	0.1891
533	rhodopsin	40 kDa	0.1891
534	HIG1 domain family member 2A, mitochondrial	15 kDa	0.1888
535	39S ribosomal protein L14, mitochondrial precursor	15 kDa	0.1888
536	guanine nucleotide-binding protein G(o) subunit alpha	40 kDa	0.1878
537	PREDICTED: required for meiotic nuclear division protein 1 homolog isoform X1	48 kDa	0.1875
538	60S ribosomal protein L31	16 kDa	0.1872
539	PREDICTED: multiple PDZ domain protein isoform X3	16 kDa	0.1872
540	ras-related protein Rab-14	24 kDa	0.1858
541	CDP-diacylglycerol--inositol 3-phosphatidyltransferase	24 kDa	0.1848
542	PREDICTED: peptidyl-prolyl cis-trans isomerase FKBP8 isoform X1	57 kDa	0.1842
543	PREDICTED: ADP-ribosylation factor 2	16 kDa	0.1841
544	mitochondrial folate transporter/carrier	33 kDa	0.1828
545	transmembrane emp24 domain-containing protein 10 precursor	24 kDa	0.1818
546	60S ribosomal protein L10	25 kDa	0.1798
547	uncharacterized protein LOC560648	16 kDa	0.1797
548	PREDICTED: protein CCSMST1	16 kDa	0.1797
549	60S ribosomal protein L10a	25 kDa	0.1769
550	UMP-CMP kinase	25 kDa	0.1769
551	PREDICTED: probable palmitoyltransferase ZDHHC14 isoform X1	16 kDa	0.1754
552	PREDICTED: protein CCSMST1	16 kDa	0.1754
553	ATP-dependent Clp protease ATP-binding subunit clpX-like, mitochondrial	68 kDa	0.175
554	enoyl-CoA delta isomerase 2, mitochondrial	42 kDa	0.1748
555	PREDICTED: carnitine palmitoyltransferase 1A isoform X2	69 kDa	0.174
556	PREDICTED: AFG3-like protein 1	60 kDa	0.1739
557	succinyl-CoA ligase	34 kDa	0.1729
558	polymerase delta-interacting protein 2	43 kDa	0.1721
559	60S acidic ribosomal protein P0	34 kDa	0.1715
560	ATP synthase subunit delta, mitochondrial	17 kDa	0.1714
561	solute carrier family 25 member 33	34 kDa	0.1709
562	mitochondrial dynamics protein MID49	52 kDa	0.1703
563	PREDICTED: mitochondrial fission 1 protein	17 kDa	0.17
564	Probable saccharopine dehydrogenase	26 kDa	0.1689
565	probable D-lactate dehydrogenase, mitochondrial	53 kDa	0.1681
566	probable asparagine--tRNA ligase, mitochondrial	26 kDa	0.168
567	PREDICTED: 40S ribosomal protein S24	17 kDa	0.1638
568	28S ribosomal protein S14, mitochondrial	17 kDa	0.1638
569	flavin containing monooxygenase 5	27 kDa	0.1631
570	microsomal glutathione S-transferase 1.1	18 kDa	0.1626
571	mitochondrial peptide methionine sulfoxide reductase	27 kDa	0.1623
572	PREDICTED: l-isoaspartyl protein carboxyl methyltransferase, like isoform X2	27 kDa	0.1623
573	mitochondrial folate transporter/carrier	36 kDa	0.161
574	protein SCO2 homolog, mitochondrial	36 kDa	0.1604
575	DnaJ (Hsp40) homolog, subfamily A, member 3B	36 kDa	0.1604
576	28S ribosomal protein S9, mitochondrial	46 kDa	0.1602
577	dihydroorotate dehydrogenase (quinone), mitochondrial	46 kDa	0.1593

578	39S ribosomal protein L20, mitochondrial	18 kDa	0.1591
579	PREDICTED: biotin--protein ligase isoform X1	93 kDa	0.1587
580	cytosol aminopeptidase	37 kDa	0.1575
581	PREDICTED: 60S ribosomal protein L27-like	18 kDa	0.1557
582	succinate dehydrogenase	18 kDa	0.1557
583	PREDICTED: dnaJ homolog subfamily C member 30	47 kDa	0.1557
584	aminomethyltransferase, mitochondrial	38 kDa	0.1542
585	40S ribosomal protein S16	18 kDa	0.1536
586	solute carrier family 3 (amino acid transporter heavy chain), member 2b	57 kDa	0.1534
587	PREDICTED: uncharacterized protein At5g50100, mitochondrial-like isoform X1	19 kDa	0.1525
588	ras-related protein Rab-3A	28 kDa	0.1519
589	uncharacterized protein LOC794398	28 kDa	0.1519
590	phosphatidylinositide phosphatase SAC1-B	67 kDa	0.1512
591	PREDICTED: 40S ribosomal protein S3-like isoform X1	28 kDa	0.1512
592	PREDICTED: sphingomyelin phosphodiesterase 2 isoform X1	48 kDa	0.1506
593	PREDICTED: serine/threonine-protein kinase DCLK1 isoform X1	38 kDa	0.1501
594	erlin-1 precursor	39 kDa	0.1481
595	mitoferrin-1	19 kDa	0.1464
596	dehydrogenase/reductase SDR family member 4	29 kDa	0.1453
597	dolichyl-diphosphooligosaccharide--protein glycosyltransferase subunit 2 precursor	70 kDa	0.1445
598	ras-related protein Rab-8B	20 kDa	0.1427
599	ATPase family AAA domain-containing protein 1-B	20 kDa	0.1427
600	peripherin-2	20 kDa	0.1418
601	complement component 1 Q subcomponent-binding protein, mitochondrial	30 kDa	0.1415
602	mitochondrial ubiquitin ligase activator of NFKB 1	40 kDa	0.1414
603	PREDICTED: NADH dehydrogenase	20 kDa	0.14
604	signal peptidase complex subunit 3	20 kDa	0.1391
605	actin-like protein 6A	31 kDa	0.138
606	PREDICTED: 60S ribosomal protein L11 isoform X2	20 kDa	0.1373
607	2-oxoisovalerate dehydrogenase subunit alpha, mitochondrial	52 kDa	0.1372
608	3-hydroxyisobutyryl-CoA hydrolase, mitochondrial	42 kDa	0.135
609	PREDICTED: protein TsetseEP-like	21 kDa	0.1348
610	ADP-ribosylation factor-like protein 2	21 kDa	0.134
611	oxoglutarate (alpha-ketoglutarate) dehydrogenase (lipoamide)	21 kDa	0.1332
612	39S ribosomal protein L12, mitochondrial	21 kDa	0.1316
613	solute carrier family 25 member 35	32 kDa	0.1309
614	PREDICTED: bcl10-interacting CARD protein isoform X2	21 kDa	0.1308
615	guanine nucleotide-binding protein subunit beta-5	43 kDa	0.1302
616	uncharacterized protein LOC393228	21 kDa	0.1301
617	PREDICTED: ras-related C3 botulinum toxin substrate 1	22 kDa	0.1286
618	PREDICTED: oxoglutarate (alpha-ketoglutarate) dehydrogenase (lipoamide) isoform X2	44 kDa	0.1279
619	dnaJ homolog subfamily A member 3, mitochondrial	44 kDa	0.1272
620	peroxiredoxin-2	22 kDa	0.1271
621	Bcl-2/adenovirus E1B 19kD interaction protein XR	22 kDa	0.1271
622	PREDICTED: uncharacterized aarF domain-containing protein kinase 1 isoform X1	22 kDa	0.1271
623	60S ribosomal protein L9	22 kDa	0.1264
624	abhydrolase domain-containing protein 4	22 kDa	0.1264
625	RPE-retinal G protein-coupled receptor	33 kDa	0.1259
626	trifunctional enzyme subunit alpha, mitochondrial	33 kDa	0.1255
627	elongation factor Ts, mitochondrial	34 kDa	0.125
628	transmembrane protein 14A	11 kDa	0.1247
629	PREDICTED: G-rich sequence factor 1 isoform X1	45 kDa	0.1247
630	ATP synthase mitochondrial F1 complex assembly factor 2	34 kDa	0.1245
631	PREDICTED: 2-oxoglutarate dehydrogenase-like, mitochondrial	115 kDa	0.1238
632	protein-S-isoprenylcysteine O-methyltransferase	34 kDa	0.1236
633	PREDICTED: aarF domain-containing protein kinase 4	11 kDa	0.1233
634	28S ribosomal protein S27, mitochondrial	46 kDa	0.1233
635	40S ribosomal protein SA	34 kDa	0.1231
636	succinyl-CoA:3-ketoacid coenzyme A transferase 1, mitochondrial	57 kDa	0.1231

637	PREDICTED: 2-oxoglutarate dehydrogenase, mitochondrial	93 kDa	0.1223
638	mitochondrial-processing peptidase subunit alpha	58 kDa	0.1223
639	PREDICTED: cytochrome b-c1 complex subunit 6, mitochondrial-like	23 kDa	0.1222
640	PREDICTED: potassium voltage-gated channel subfamily B member 2	93 kDa	0.1215
641	PREDICTED: glycerol-3-phosphate acyltransferase 1, mitochondrial	93 kDa	0.1215
642	electron transfer flavoprotein-ubiquinone oxidoreductase, mitochondrial	23 kDa	0.1202
643	PREDICTED: mitochondrial thiamine pyrophosphate carrier isoform X1	35 kDa	0.12
644	adipocyte plasma membrane-associated protein	47 kDa	0.1199
645	PREDICTED: glycerol kinase isoform X4	59 kDa	0.1196
646	flotillin 2	47 kDa	0.1193
647	ras-related protein Rab-18-B	23 kDa	0.1189
648	flotillin-2a	47 kDa	0.1183
649	surfeit gene 4, like	23 kDa	0.1176
650	long-chain fatty acid transport protein 1	72 kDa	0.1173
651	PREDICTED: heme oxygenase 2	36 kDa	0.1166
652	ras-related protein Rab-5B	24 kDa	0.1157
653	long-chain fatty acid transport protein 4	73 kDa	0.1157
654	lysine--tRNA ligase	60 kDa	0.1156
655	L-2-hydroxyglutarate dehydrogenase, mitochondrial	12 kDa	0.1144
656	coiled-coil domain containing 56-like	12 kDa	0.1144
657	PREDICTED: mitochondrial coenzyme A transporter SLC25A42 isoform X1	36 kDa	0.1138
658	PREDICTED: ER membrane protein complex subunit 1 isoform X1	111 kDa	0.1134
659	PREDICTED: 3-oxoacyl-[acyl-carrier-protein] synthase, mitochondrial-like isoform X2	12 kDa	0.1132
660	PREDICTED: regulator of G-protein signaling 9 isoform X2	62 kDa	0.113
661	PREDICTED: multiple PDZ domain protein isoform X1	37 kDa	0.1126
662	PREDICTED: sialic acid-binding Ig-like lectin 6	24 kDa	0.1122
663	protein FAM173A	24 kDa	0.1122
664	PREDICTED: patatin-like phospholipase domain-containing protein 4	12 kDa	0.112
665	adenylyl cyclase-associated protein 1	50 kDa	0.112
666	PREDICTED: peptidyl-prolyl cis-trans isomerase FKBP8 isoform X1	37 kDa	0.1111
667	PREDICTED: ammonium transporter Rh type A isoform X1	12 kDa	0.1109
668	ubiquitin-60S ribosomal protein L40	12 kDa	0.1109
669	PREDICTED: SRA stem-loop-interacting RNA-binding protein, mitochondrial	12 kDa	0.1098
670	PREDICTED: uncharacterized protein NCBP2-AS2-like	12 kDa	0.1098
671	superoxide dismutase	25 kDa	0.1094
672	guanylate kinase 1b	25 kDa	0.1094
673	GTP:AMP phosphotransferase AK3, mitochondrial	25 kDa	0.1089
674	PREDICTED: ER membrane protein complex subunit 6	12 kDa	0.1087
675	PREDICTED: 39S ribosomal protein L53, mitochondrial-like	12 kDa	0.1087
676	PREDICTED: glutaminase kidney isoform, mitochondrial isoform X2	38 kDa	0.1086
677	serine/threonine-protein kinase DCLK2 isoform 1	90 kDa	0.1084
678	cytochrome c oxidase assembly factor 3 homolog, mitochondrial	12 kDa	0.1077
679	dolichyl-diphosphooligosaccharide--protein glycosyltransferase 48 kDa subunit precursor	51 kDa	0.1074
680	28S ribosomal protein S34, mitochondrial	25 kDa	0.1073
681	apolipoprotein O	25 kDa	0.1063
682	propionyl-CoA carboxylase alpha chain, mitochondrial	79 kDa	0.1058
683	PREDICTED: 40S ribosomal protein S2	12 kDa	0.1056
684	PREDICTED: cytochrome c oxidase subunit 7A-related protein, mitochondrial-like	12 kDa	0.1056
685	60S ribosomal protein L35a	13 kDa	0.1046
686	NADH dehydrogenase (ubiquinone) iron-sulfur protein 5	13 kDa	0.1046
687	28S ribosomal protein S33, mitochondrial	13 kDa	0.1046
688	solute carrier family 2, facilitated glucose transporter member 1	53 kDa	0.1043
689	PREDICTED: fatty aldehyde dehydrogenase isoform X2	40 kDa	0.1038
690	oxoglutarate (alpha-ketoglutarate) dehydrogenase (lipoamide)	26 kDa	0.1038
691	PREDICTED: LOW QUALITY PROTEIN: lysosome membrane protein 2	26 kDa	0.1038
692	pyruvate dehydrogenase E1 component subunit alpha, somatic form, mitochondrial	13 kDa	0.1036
693	uncharacterized aarF domain-containing protein kinase 1	13 kDa	0.1036
694	PREDICTED: LOW QUALITY PROTEIN: lysosome membrane protein 2	53 kDa	0.1029

695	diablo, IAP-binding mitochondrial protein a	13 kDa	0.1026
696	bcl-2-like protein 1	26 kDa	0.1023
697	PREDICTED: oxoglutarate (alpha-ketoglutarate) dehydrogenase (lipoamide) isoform X3	26 kDa	0.1023
698	peroxisomal biogenesis factor 3	26 kDa	0.1023
699	BRI3-binding protein precursor	26 kDa	0.1023
700	coenzyme Q-binding protein COQ10 homolog, mitochondrial	27 kDa	0.1014
701	39S ribosomal protein L17, mitochondrial	13 kDa	0.1008
702	flotillin-1	41 kDa	0.1006
703	PREDICTED: sodium/potassium/calcium exchanger 2-like isoform X2	68 kDa	0.1006
704	PREDICTED: ceroid-lipofuscinosis, neuronal 6a isoform X1	19 kDa	0.0999
705	PREDICTED: fatty aldehyde dehydrogenase-like	55 kDa	0.0998
706	PREDICTED: aarF domain-containing protein kinase 4	70 kDa	0.0986
707	band 3 anion transport protein	27 kDa	0.0982
708	PREDICTED: transmembrane emp24 domain-containing protein 7 isoform X2	27 kDa	0.0982
709	PREDICTED: 40S ribosomal protein S20	13 kDa	0.0981
710	PREDICTED: transmembrane protein 186 isoform X1	13 kDa	0.0981
711	FAM82B	13 kDa	0.0981
712	cone cGMP-specific 3',5'-cyclic phosphodiesterase subunit alpha'	98 kDa	0.098
713	phosphofructokinase, muscle b	70 kDa	0.0978
714	PREDICTED: prostate stem cell antigen-like	13 kDa	0.0972
715	PREDICTED: small integral membrane protein 8 isoform X2	13 kDa	0.0972
716	UPF0545 protein C22orf39 homolog	14 kDa	0.0964
717	PREDICTED: sideroflexin-2 isoform X3	14 kDa	0.0964
718	PREDICTED: renin receptor isoform X1	42 kDa	0.096
719	60S ribosomal protein L8	28 kDa	0.0956
720	PREDICTED: ubiquitin carboxyl-terminal hydrolase 30 isoform X1	57 kDa	0.0953
721	2-oxoisovalerate dehydrogenase subunit beta, mitochondrial	43 kDa	0.0951
722	PREDICTED: cytochrome c oxidase subunit 5B, mitochondrial-like	14 kDa	0.0947
723	transmembrane protein 33	29 kDa	0.094
724	heat shock cognate 71 kDa protein	29 kDa	0.094
725	PREDICTED: 60S ribosomal protein L36a-like	14 kDa	0.0939
726	probable carboxypeptidase PM20D1.2 precursor	58 kDa	0.0935
727	monoacylglycerol lipase ABHD12	44 kDa	0.0932
728	tyrosine--tRNA ligase, mitochondrial	14 kDa	0.0931
729	acyl-coenzyme A thioesterase THEM4	29 kDa	0.0928
730	ATPase family AAA domain-containing protein 1-A isoform 1	44 kDa	0.0922
731	ATP-dependent Clp protease proteolytic subunit, mitochondrial	29 kDa	0.092
732	peroxisomal biogenesis factor 3	14 kDa	0.0915
733	28S ribosomal protein S2, mitochondrial	29 kDa	0.0909
734	PREDICTED: anoctamin-8	14 kDa	0.0908
735	PREDICTED: 40S ribosomal protein S26-like	14 kDa	0.0908
736	protein kinase, cAMP-dependent, regulatory, type II, alpha A	45 kDa	0.0904
737	glutamate dehydrogenase 1b	60 kDa	0.0902
738	ER membrane protein complex subunit 3	30 kDa	0.0898
739	methylmalonic aciduria type A protein, mitochondrial	30 kDa	0.0898
740	1-acyl-sn-glycerol-3-phosphate acyltransferase gamma	45 kDa	0.0895
741	alpha-1,3/1,6-mannosyltransferase ALG2	46 kDa	0.0887
742	PREDICTED: ATP synthase-coupling factor 6, mitochondrial isoform X1	15 kDa	0.0886
743	uncharacterized protein C19orf52 homolog	30 kDa	0.0883
744	putative hexokinase HKDC1	61 kDa	0.0882
745	F-box/LRR-repeat protein 2	46 kDa	0.088
746	membrane magnesium transporter 1 precursor	15 kDa	0.0879
747	28S ribosomal protein S6, mitochondrial	15 kDa	0.0879
748	cytochrome P450, family 27, subfamily C, polypeptide 1	62 kDa	0.0877
749	40S ribosomal protein S6	31 kDa	0.0866
750	39S ribosomal protein L51, mitochondrial precursor	15 kDa	0.0858
751	PREDICTED: neuronal migration protein doublecortin isoform X2	31 kDa	0.0849
752	holocytochrome c synthetase a	32 kDa	0.084
753	iron-sulfur cluster assembly 1 homolog, mitochondrial precursor	15 kDa	0.0839

754	uncharacterized protein LOC541327	15 kDa	0.0839
755	PREDICTED: histidine triad nucleotide-binding protein 2, mitochondrial-like isoform X1	15 kDa	0.0839
756	PREDICTED: serine/threonine-protein phosphatase PGAM5, mitochondrial isoform X1	32 kDa	0.0833
757	PREDICTED: carnitine O-palmitoyltransferase 1, liver isoform isoform X2	48 kDa	0.0827
758	protein phosphatase PTC7 homolog	32 kDa	0.0821
759	3-hydroxyisobutyrate dehydrogenase, mitochondrial	33 kDa	0.0815
760	60S ribosomal protein L28	16 kDa	0.0814
761	40S ribosomal protein S3a	33 kDa	0.0812
762	c3orf33 homolog	16 kDa	0.0808
763	PREDICTED: aspartate beta-hydroxylase isoform X2	33 kDa	0.0806
764	citrate lyase subunit beta-like protein, mitochondrial	33 kDa	0.0803
765	PREDICTED: mitofusin-1-like	84 kDa	0.08
766	PREDICTED: uncharacterized protein LOC323326 isoform X1	33 kDa	0.0797
767	transmembrane protein 177	34 kDa	0.0786
768	ubiquitin-conjugating enzyme E2Nb	16 kDa	0.0785
769	glutathione peroxidase 1	16 kDa	0.0785
770	PREDICTED: kynurenine--oxoglutarate transaminase 1 isoform X1	51 kDa	0.078
771	exonuclease 3'-5' domain-containing protein 2	69 kDa	0.0775
772	cytochrome P450, family 20, subfamily A, polypeptide 1	52 kDa	0.0773
773	PREDICTED: phosphatidate cytidylyltransferase 1	52 kDa	0.0768
774	evolutionarily conserved signaling intermediate in Toll pathway, mitochondrial	52 kDa	0.0768
775	band 3 anion transport protein	35 kDa	0.0761
776	PREDICTED: metal transporter CNNM4	35 kDa	0.0751
777	uncharacterized protein C15orf61 homolog	17 kDa	0.0747
778	2,4-dienoyl-CoA reductase, mitochondrial	35 kDa	0.0746
779	transitional endoplasmic reticulum ATPase	89 kDa	0.0745
780	PREDICTED: 40S ribosomal protein S23-like	17 kDa	0.0742
781	dnaJ homolog subfamily C member 15	17 kDa	0.0742
782	PREDICTED: 60S ribosomal protein L23	17 kDa	0.0737
783	microsomal glutathione S-transferase 1.2	17 kDa	0.0737
784	PREDICTED: creatine kinase S-type, mitochondrial isoform X2	17 kDa	0.0737
785	glutaredoxin-related protein 5, mitochondrial	17 kDa	0.0737
786	PREDICTED: syntaxin-18 isoform X1	36 kDa	0.0736
787	3-hydroxyisobutyrate dehydrogenase a	36 kDa	0.0733
788	hexaprenyldihydroxybenzoate methyltransferase, mitochondrial	36 kDa	0.0733
789	putative Ras-related protein Rab-42	18 kDa	0.0732
790	ragulator complex protein LAMTOR1	18 kDa	0.0732
791	PREDICTED: 60S ribosomal protein L12	18 kDa	0.0728
792	PREDICTED: tubulin beta-1 chain	18 kDa	0.0728
793	glycerophosphodiester phosphodiesterase 1	36 kDa	0.0726
794	PREDICTED: acyl carrier protein, mitochondrial	18 kDa	0.0723
795	mitoferrin-1	18 kDa	0.0723
796	fumarate hydratase, mitochondrial precursor	55 kDa	0.0721
797	mitochondria-eating protein	55 kDa	0.0719
798	uncharacterized protein LOC768289	37 kDa	0.0709
799	PREDICTED: calcium-binding mitochondrial carrier protein SCaMC-1 isoform X1	37 kDa	0.0707
800	protein SERAC1	18 kDa	0.0704
801	PREDICTED: protein SERAC1 isoform X2	18 kDa	0.0704
802	PREDICTED: histidine triad nucleotide-binding protein 3 isoform X1	18 kDa	0.07
803	PREDICTED: ATP-binding cassette sub-family B member 10, mitochondrial	18 kDa	0.07
804	arginase-2, mitochondrial	38 kDa	0.0694
805	PREDICTED: sodium/potassium-transporting ATPase subunit beta-2-like	19 kDa	0.0691
806	PREDICTED: 60S ribosomal protein L21	19 kDa	0.0691
807	complexin 4b	19 kDa	0.0682
808	eukaryotic translation initiation factor 4A, isoform 1A	39 kDa	0.0668
809	PREDICTED: DNA polymerase subunit gamma-2, mitochondrial	39 kDa	0.0668
810	fatty-acid amide hydrolase 2-A	59 kDa	0.0664
811	polyribonucleotide nucleotidyltransferase 1, mitochondrial	60 kDa	0.0653
812	28S ribosomal protein S22, mitochondrial	40 kDa	0.0652

813	keratin, type II cytoskeletal 8	61 kDa	0.0651
814	PREDICTED: armadillo repeat-containing protein 10	20 kDa	0.0646
815	cAMP-dependent protein kinase catalytic subunit beta	41 kDa	0.0643
816	abhydrolase domain-containing protein 16A	20 kDa	0.0642
817	methylcrotonoyl-CoA carboxylase beta chain, mitochondrial	62 kDa	0.0638
818	PREDICTED: clathrin coat assembly protein AP180 isoform X5	41 kDa	0.0637
819	reticulon-1 isoform 1	83 kDa	0.0633
820	synembryn-A	62 kDa	0.0632
821	PREDICTED: coenzyme Q-binding protein COQ10 homolog A, mitochondrial	20 kDa	0.0631
822	minor histocompatibility antigen H13	20 kDa	0.0631
823	ADP-ribosylation factor-like protein 1	20 kDa	0.0628
824	PREDICTED: synembryn-A isoform X1	63 kDa	0.0625
825	L-threonine 3-dehydrogenase, mitochondrial	42 kDa	0.0625
826	PREDICTED: signal peptidase complex catalytic subunit SEC11A-like	21 kDa	0.0621
827	leucine-rich repeat, immunoglobulin-like and transmembrane domains 1 precursor	64 kDa	0.0613
828	brain creatine kinase b	43 kDa	0.0606
829	PREDICTED: translocon-associated protein subunit gamma	21 kDa	0.0603
830	valacyclovir hydrolase	21 kDa	0.0597
831	reticulon-1 isoform 1	88 kDa	0.0596
832	PREDICTED: neuroplastin-like	44 kDa	0.0596
833	PREDICTED: transmembrane and coiled-coil domains protein 1-like	21 kDa	0.0594
834	phosphatidylserine synthase 1	21 kDa	0.0594
835	isochorismatase domain-containing protein 2, mitochondrial	21 kDa	0.0594
836	heat shock protein HSP 90-beta	89 kDa	0.0591
837	ADP-ribosylation-like factor 6 interacting protein 5	22 kDa	0.059
838	ADP-ribosylation factor-like 7	22 kDa	0.059
839	PREDICTED: BCL2/adenovirus E1B 19 kDa protein-interacting protein 3 isoform X1	22 kDa	0.0587
840	lactation elevated protein 1 homolog B	22 kDa	0.0581
841	immunoglobulin superfamily member 8 precursor	68 kDa	0.0578
842	tyrosine-protein phosphatase non-receptor type 1	22 kDa	0.0572
843	PREDICTED: branched-chain-amino-acid aminotransferase, mitochondrial isoform X2	45 kDa	0.0569
844	PREDICTED: probable arginine--tRNA ligase, mitochondrial isoform X1	22 kDa	0.0569
845	PREDICTED: GTP-binding protein SAR1b	22 kDa	0.0569
846	28S ribosomal protein S29, mitochondrial	46 kDa	0.0567
847	PREDICTED: peptide chain release factor 1, mitochondrial-like	46 kDa	0.0565
848	PREDICTED: 60S ribosomal protein L18a-like	23 kDa	0.0563
849	solute carrier family 7, member 3	70 kDa	0.056
850	bcl2-associated X protein, b	23 kDa	0.0557
851	transmembrane protein 160	23 kDa	0.0557
852	synaptotagmin II	47 kDa	0.0555
853	ras-related protein Rab-24	23 kDa	0.0555
854	PREDICTED: short/branched chain specific acyl-CoA dehydrogenase, mitochondrial	47 kDa	0.0552
855	lamin-B2	23 kDa	0.0549
856	PREDICTED: carbonyl reductase family member 4 isoform X1	23 kDa	0.0544
857	PREDICTED: acyl-CoA thioesterase 9, tandem duplicate 1 isoform X1	23 kDa	0.0541
858	60S ribosomal protein L3	48 kDa	0.054
859	methylcrotonoyl-CoA carboxylase subunit alpha, mitochondrial	72 kDa	0.0538
860	PREDICTED: NADPH--cytochrome P450 reductase isoform X1	48 kDa	0.0536
861	uncharacterized protein LOC100005854	24 kDa	0.0536
862	acylpyruvase FAHD1, mitochondrial	24 kDa	0.0536
863	keratin, type I cytoskeletal 18	49 kDa	0.0528
864	PREDICTED: neural cell adhesion molecule 1 isoform X1	74 kDa	0.0527
865	PREDICTED: protein TBRG4	74 kDa	0.0526
866	28S ribosomal protein S18a, mitochondrial	24 kDa	0.0525
867	PREDICTED: probable protein-cysteine N-palmitoyltransferase porcupine isoform X1	25 kDa	0.0513
868	PREDICTED: 60S ribosomal protein L13a, partial	25 kDa	0.0506
869	PREDICTED: phosphofructokinase, muscle b isoform X1	25 kDa	0.0506
870	oxidase (cytochrome c) assembly 1-like	51 kDa	0.0503
871	PREDICTED: V-type proton ATPase subunit S1	52 kDa	0.0497

872	60S ribosomal protein L17	25 kDa	0.0497
873	adenylate kinase 4, mitochondrial	25 kDa	0.0497
874	PREDICTED: mitochondrial antiviral-signaling protein isoform X1	52 kDa	0.0496
875	epoxide hydrolase 1	52 kDa	0.0493
876	PREDICTED: protein transport protein Sec61 subunit alpha-like 1	52 kDa	0.0491
877	cardiolipin synthase	26 kDa	0.049
878	squalene synthase	53 kDa	0.0486
879	glutathione S-transferase kappa 1	26 kDa	0.0486
880	bifunctional coenzyme A synthase precursor	53 kDa	0.0484
881	putative hexokinase HKDC1	26 kDa	0.0484
882	PREDICTED: phospholipid scramblase 2	26 kDa	0.0477
883	BRI3-binding protein precursor	26 kDa	0.0477
884	PREDICTED: ATP synthase subunit s-like protein isoform X1	27 kDa	0.0473
885	PREDICTED: translation initiation factor IF-3, mitochondrial	27 kDa	0.0473
886	phosducin	27 kDa	0.0467
887	PREDICTED: succinate-semialdehyde dehydrogenase, mitochondrial isoform X1	55 kDa	0.0464
888	von Willebrand factor A domain-containing protein 8	27 kDa	0.0463
889	PREDICTED: tudor and KH domain-containing protein isoform X1	56 kDa	0.046
890	diablo homolog, mitochondrial	27 kDa	0.0459
891	4-aminobutyrate aminotransferase, mitochondrial	56 kDa	0.0457
892	PREDICTED: reticulon-1 isoform X1	85 kDa	0.0455
893	HCLS1-associated protein X-1	28 kDa	0.0454
894	dol-P-Man:Man(7)GlcNAc(2)-PP-Dol alpha-1,6-mannosyltransferase precursor	56 kDa	0.0453
895	syntaxin binding protein 1b	57 kDa	0.045
896	dolichol-phosphate mannosyltransferase subunit 1	28 kDa	0.045
897	apoptogenic protein 1, mitochondrial	28 kDa	0.045
898	melanoregulin	28 kDa	0.045
899	PREDICTED: vesicle-fusing ATPase isoform X1	86 kDa	0.0447
900	acyl-CoA thioesterase 9, tandem duplicate 1	28 kDa	0.0446
901	PREDICTED: Golgi SNAP receptor complex member 1 isoform X1	28 kDa	0.0444
902	39S ribosomal protein L9, mitochondrial	28 kDa	0.0443
903	phosducin 2	29 kDa	0.0441
904	PREDICTED: alpha-aminoadipic semialdehyde dehydrogenase	59 kDa	0.0432
905	internexin neuronal intermediate filament protein, alpha	59 kDa	0.0432
906	glutamyl-tRNA(Gln) amidotransferase subunit A, mitochondrial	59 kDa	0.0432
907	PREDICTED: ATP-binding cassette sub-family B member 10, mitochondrial	29 kDa	0.043
908	probable tRNA N6-adenosine threonylcarbamoyltransferase, mitochondrial	29 kDa	0.043
909	39S ribosomal protein L10, mitochondrial precursor	29 kDa	0.043
910	ES1 protein homolog, mitochondrial	29 kDa	0.0429
911	PREDICTED: E3 ubiquitin-protein ligase RNF170-like	29 kDa	0.0427
912	PREDICTED: mitochondrial 10-formyltetrahydrofolate dehydrogenase	90 kDa	0.0426
913	uncharacterized protein LOC619266 precursor	60 kDa	0.0426
914	catechol O-methyltransferase	30 kDa	0.0424
915	PREDICTED: calcium signal-modulating cyclophilin ligand isoform X1	30 kDa	0.0419
916	uncharacterized protein LOC447917 precursor	30 kDa	0.0416
917	28S ribosomal protein S15, mitochondrial	30 kDa	0.0414
918	PREDICTED: outer dense fiber protein 2	96 kDa	0.04
919	phosphatidylinositol transfer protein beta isoform	32 kDa	0.0396
920	thioredoxin-related transmembrane protein 1 precursor	32 kDa	0.0392
921	PREDICTED: ethylmalonyl-CoA decarboxylase isoform X1	32 kDa	0.0392
922	PREDICTED: enoyl-CoA hydratase domain-containing protein 3, mitochondrial-like	32 kDa	0.0392
923	PREDICTED: trimethyllysine dioxygenase, mitochondrial	32 kDa	0.0392
924	PREDICTED: proline-rich protein 18-like	32 kDa	0.0388
925	PREDICTED: serine/threonine-protein phosphatase 2A 65 kDa regulatory subunit A beta isoform isoform X1	65 kDa	0.0387
926	PREDICTED: ATP-dependent (S)-NAD(P)H-hydrate dehydratase isoform X10	32 kDa	0.0386
927	PREDICTED: pyruvate dehydrogenase phosphatase regulatory subunit, mitochondrial-like	99 kDa	0.0385
928	PREDICTED: translocon-associated protein subunit alpha-like isoform X3	32 kDa	0.0385
929	synaptophysin b isoform 1	33 kDa	0.0384

930	PREDICTED: ATP-binding cassette sub-family B member 10, mitochondrial	33 kDa	0.0382
931	PREDICTED: LOW QUALITY PROTEIN: metalloendopeptidase OMA1, mitochondrial	33 kDa	0.038
932	3-mercaptopyruvate sulfurtransferase	33 kDa	0.0378
933	PREDICTED: translation factor GUF1, mitochondrial	33 kDa	0.0378
934	PREDICTED: protein FAM134C	33 kDa	0.0377
935	carnitine O-acetyltransferase	68 kDa	0.0375
936	PREDICTED: protein CLN8 isoform X1	34 kDa	0.0372
937	NAD-dependent protein deacylase sirtuin-5, mitochondrial	34 kDa	0.0372
938	acyl-CoA synthetase long-chain family member 1	68 kDa	0.037
939	malonyl-CoA decarboxylase, mitochondrial	34 kDa	0.0368
940	uncharacterized protein LOC100001340	34 kDa	0.0366
941	enoyl-CoA hydratase domain-containing protein 2, mitochondrial	35 kDa	0.0361
942	heme oxygenase 2	35 kDa	0.036
943	PREDICTED: CAAX prenyl protease 2	35 kDa	0.0354
944	PREDICTED: stromal interaction molecule 1 isoform X3	72 kDa	0.0352
945	PREDICTED: lon protease homolog, mitochondrial	108 kDa	0.0352
946	iron-sulfur protein NUBPL	36 kDa	0.0351
947	thioredoxin-related transmembrane protein 2-B precursor	36 kDa	0.0349
948	isoleucine--tRNA ligase, mitochondrial	110 kDa	0.0345
949	PREDICTED: transmembrane protein 43-like	37 kDa	0.0341
950	glutamyl-tRNA(Gln) amidotransferase subunit B, mitochondrial	37 kDa	0.0339
951	PREDICTED: protein angel homolog 1	37 kDa	0.0337
952	UDP-xylose and UDP-N-acetylglucosamine transporter	37 kDa	0.0335
953	uncharacterized protein LOC559844	37 kDa	0.0334
954	casein kinase 1, alpha 1	38 kDa	0.0332
955	39S ribosomal protein L39, mitochondrial	38 kDa	0.0332
956	neurotrimin isoform 1 precursor	38 kDa	0.0328
957	PREDICTED: monoacylglycerol lipase abhd6-A	38 kDa	0.0327
958	ATPase asna1	38 kDa	0.0326
959	threonine aldolase 1	38 kDa	0.0325
960	protein phosphatase methylesterase 1	39 kDa	0.0324
961	PREDICTED: tectonic-2	39 kDa	0.0322
962	peripherin 2b (retinal degeneration, slow)	39 kDa	0.0321
963	heterogeneous nuclear ribonucleoprotein K	39 kDa	0.0321
964	PREDICTED: nucleolin isoform X5	79 kDa	0.032
965	PREDICTED: striatin-3 isoform X1	79 kDa	0.032
966	PREDICTED: ATP-dependent RNA helicase DDX3X isoform X6	79 kDa	0.0319
967	opsin-1, short-wave-sensitive 1	39 kDa	0.0318
968	protein phosphatase 1 regulatory subunit 7 isoform 1	40 kDa	0.0315
969	lectin, mannose-binding 2-like b precursor	40 kDa	0.0314
970	PREDICTED: ATP-dependent zinc metalloprotease YME1L1-like isoform X1	81 kDa	0.0312
971	PREDICTED: KH domain containing, RNA binding, signal transduction associated 1a isoform X2	41 kDa	0.0307
972	UPF0554 protein C2orf43 homolog	41 kDa	0.0303
973	elongation factor G, mitochondrial	83 kDa	0.0302
974	major histocompatibility complex class I UEA precursor	41 kDa	0.0301
975	PREDICTED: 11-cis retinol dehydrogenase isoform X1	41 kDa	0.0301
976	cytosolic 5'-nucleotidase 1A	41 kDa	0.0301
977	methylmalonyl-CoA mutase, mitochondrial	84 kDa	0.03
978	eukaryotic translation initiation factor 3 subunit H-A	42 kDa	0.0298
979	immunity-related GTPase family, q2	42 kDa	0.0297
980	LETM1 domain-containing protein 1	42 kDa	0.0296
981	PREDICTED: protein XRP2 isoform X1	42 kDa	0.0296
982	NAD-dependent protein deacetylase sirtuin-2	42 kDa	0.0296
983	ATP-dependent RNA helicase SUPV3L1, mitochondrial precursor	86 kDa	0.0293
984	nucleoside diphosphate-linked moiety X motif 8, mitochondrial	43 kDa	0.0292
985	4-hydroxybenzoate polyprenyltransferase, mitochondrial	43 kDa	0.029
986	solute carrier family 43 member 3	43 kDa	0.0287
987	acyl-CoA:lysophosphatidylglycerol acyltransferase 1	43 kDa	0.0287
988	mitoferrin-2	44 kDa	0.0283

989	abhydrolase domain-containing protein 4	44 kDa	0.0283
990	basigin precursor	45 kDa	0.0278
991	isobutyryl-CoA dehydrogenase, mitochondrial	45 kDa	0.0274
992	PREDICTED: ATPase, Ca++ transporting, cardiac muscle, slow twitch 2b isoform X1	92 kDa	0.0274
993	endoplasmin precursor	92 kDa	0.0274
994	striatin	92 kDa	0.0273
995	casein kinase 2, alpha 1 polypeptide	46 kDa	0.0272
996	PREDICTED: vacuole membrane protein 1 isoform X1	46 kDa	0.0271
997	glycosaminoglycan xylosylkinase	46 kDa	0.0271
998	PREDICTED: protein NDRG3 isoform X1	46 kDa	0.0269
999	prominin-1 precursor	94 kDa	0.0268
1000	cytochrome P450 2U1	46 kDa	0.0268
1001	PREDICTED: heterogeneous nuclear ribonucleoprotein Q isoform X2	47 kDa	0.0265
1002	PREDICTED: serine protease HTRA2, mitochondrial	47 kDa	0.0263
1003	tapasin-like	48 kDa	0.0261
1004	PREDICTED: ubiquitin associated protein 2b isoform X1	48 kDa	0.0261
1005	26S proteasome non-ATPase regulatory subunit 3	48 kDa	0.026
1006	PREDICTED: poly(A) RNA polymerase, mitochondrial	48 kDa	0.026
1007	uncharacterized protein C6orf136 homolog	48 kDa	0.0258
1008	sarcolemma associated protein b	48 kDa	0.0257
1009	uncharacterized protein LOC405817	49 kDa	0.0254
1010	PREDICTED: uncharacterized protein LOC557028 isoform X2	49 kDa	0.0253
1011	PREDICTED: ATP-binding cassette sub-family B member 6, mitochondrial isoform X1	99 kDa	0.0253
1012	PREDICTED: mannosyl-oligosaccharide glucosidase isoform X1	49 kDa	0.0253
1013	39S ribosomal protein L37, mitochondrial	49 kDa	0.0252
1014	TLD domain-containing protein 1	50 kDa	0.025
1015	PREDICTED: uncharacterized aarF domain-containing protein kinase 2	50 kDa	0.0248
1016	PREDICTED: drebrin isoform X2	50 kDa	0.0248
1017	PREDICTED: uncharacterized protein si:ch211-11k18.4	51 kDa	0.0245
1018	elongation factor 1-gamma	51 kDa	0.0244
1019	UBX domain-containing protein 4	52 kDa	0.024
1020	protein disulfide-isomerase TMX3 precursor	52 kDa	0.0238
1021	N-acylneuraminate cytidylyltransferase	53 kDa	0.0236
1022	F-box/LRR-repeat protein 4	53 kDa	0.0232
1023	PREDICTED: protein LYRIC isoform X1	53 kDa	0.0231
1024	protein crumbs homolog 2	54 kDa	0.0229
1025	phosphatidate cytidylyltransferase 2	54 kDa	0.0227
1026	coiled-coil domain-containing protein 47 precursor	56 kDa	0.0221
1027	aldehyde dehydrogenase 2 family (mitochondrial), tandem duplicate 2	57 kDa	0.0218
1028	pyruvate dehyrogenase phosphatase catalytic subunit 1	57 kDa	0.0218
1029	PREDICTED: lysophosphatidylcholine acyltransferase 1-like	59 kDa	0.021
1030	amine oxidase	59 kDa	0.021
1031	prenylcysteine oxidase 1 precursor	59 kDa	0.0208
1032	PREDICTED: pyruvate kinase PKM isoform X1	60 kDa	0.0206
1033	retinoid isomerohydrolase	61 kDa	0.0203
1034	PREDICTED: GPI transamidase component PIG-S isoform X2	63 kDa	0.0197
1035	T-complex protein 1 subunit gamma	63 kDa	0.0196
1036	PREDICTED: serine beta-lactamase-like protein LACTB, mitochondrial isoform X1	64 kDa	0.0192
1037	transmembrane anterior posterior transformation protein 1 homolog	66 kDa	0.0188
1038	protein FAM73B	66 kDa	0.0188
1039	PREDICTED: long-chain fatty acid transport protein 6-like isoform X1	69 kDa	0.0179
1040	PREDICTED: proline dehydrogenase 1, mitochondrial-like	69 kDa	0.0178
1041	polyadenylate-binding protein 1	71 kDa	0.0174
1042	membrane protein, palmitoylated 5b (MAGUK p55 subfamily member 5)	71 kDa	0.0174
1043	PREDICTED: gamma-glutamyltransferase 7 isoform X1	72 kDa	0.0171
1044	PREDICTED: TBC1 domain family member 17 isoform X1	73 kDa	0.0168
1045	PREDICTED: caseinolytic peptidase B protein homolog	74 kDa	0.0166
1046	lipase maturation factor 2	76 kDa	0.0161
1047	transferrin receptor protein 1	77 kDa	0.0161

1048	PREDICTED: probable threonine--tRNA ligase 2, cytoplasmic-like	78 kDa	0.0158
1049	PREDICTED: reticulon-4 isoform X1	80 kDa	0.0153
1050	protein crumbs homolog 2	162 kDa	0.0153
1051	PREDICTED: cyclic nucleotide-gated channel rod photoreceptor subunit alpha-like	82 kDa	0.0151
1052	PREDICTED: amyloid beta A4 protein-like isoform X2	84 kDa	0.0146
1053	PREDICTED: dolichyl-diphosphooligosaccharide--protein glycosyltransferase subunit STT3B	91 kDa	0.0135
1054	importin subunit beta-1	97 kDa	0.0126
1055	PREDICTED: dynamin 1a isoform X5	99 kDa	0.0124
1056	PREDICTED: bifunctional heparan sulfate N-deacetylase/N-sulfotransferase 1	102 kDa	0.0121
1057	alpha-aminoadipic semialdehyde synthase, mitochondrial	105 kDa	0.0117
1058	PREDICTED: sarcoplasmic/endoplasmic reticulum calcium ATPase 2	116 kDa	0.0106
1059	von Willebrand factor A domain-containing protein 8	126 kDa	0.0097
1060	PREDICTED: pyruvate carboxylase, mitochondrial-like	130 kDa	0.0094
1061	insulin-like growth factor 1b receptor precursor	158 kDa	0.0078
1062	PREDICTED: crumbs family member 2b isoform X1	160 kDa	0.0076
1063	PREDICTED: rootletin isoform X1	226 kDa	0.0054
1064	PREDICTED: midasin	512 kDa	0.0024
